# Ribosomal background of the *Bacillus cereus* group thermotypes

**DOI:** 10.1038/srep46430

**Published:** 2017-04-13

**Authors:** Krzysztof Fiedoruk, Justyna M. Drewnowska, Tamara Daniluk, Katarzyna Leszczynska, Piotr Iwaniuk, Izabela Swiecicka

**Affiliations:** 1 Department of Microbiology, Medical University of Bialystok, Bialystok, Poland; 2 Department of Microbiology, Institute of Biology, University of Bialystok, Bialystok, Poland; 3 Laboratory of Applied Microbiology, University of Bialystok, Bialystok, Poland

## Abstract

In this study we reconstructed the architecture of *Bacillus cereus sensu lato* population based on ribosomal proteins, and identified a link between the ribosomal proteins’ variants and thermal groups (thermotypes) of the bacilli. The *in silico* phyloproteomic analysis of 55 ribosomal proteins (34 large and 21 small subunit r-proteins) of 421 strains, representing 14 well-established or plausible *B. cereus sensu lato* species, revealed several ribosomal clusters (r-clusters), which in general were well correlated with the strains’ affiliation to phylogenetic/thermal groups I–VII. However, a conformity and possibly a thermal characteristic of certain phylogenetic groups, e.g. the group IV, were not supported by a distribution of the corresponding r-clusters, and consequently neither by the analysis of cold-shock proteins (CSPs) nor by a content of heat shock proteins (HSPs). Furthermore, a preference for isoleucine and serine over valine and alanine in r-proteins along with a lack of HSP16.4 were recognized in non-mesophilic thermotypes. In conclusion, we suggest that the observed divergence in ribosomal proteins may be connected with an adaptation of *B. cereus sensu lato* members to various thermal niches.

*Bacillus cereus sensu lato*, also known as the *B. cereus* group, includes closely related Gram-positive, spore-forming and aerobic bacilli, commonly present in various natural environments^1^ and food matrices^2^. *Bacillus cereus sensu stricto* (hereinafter *B. cereus*), an opportunistic pathogen, *Bacillus anthracis*, the etiological agent of anthrax, and *Bacillus thuringiensis*, an entomopathogen widely used as a biopesticide, are founders of the group. The *B. cereus s.l.* also comprises bacteria of minor medical and/or economic significance, such as *Bacillus mycoides* and *Bacillus pseudomycoides*^3^, *Bacillus weihenstephanensis*^4^, *Bacillus toyonensis*^5^, and *Bacillus cytotoxicus*^6^. Recently, *Bacillus manliponensis*^7^, *Bacillus gaemokensis*^8^, *Bacillus bombysepticus*^9^, *Bacillus bingmayongensis*^10^, *Bacillus* sp. 7_6_55CFAA_CT2^11^, and *Bacillus wiedmannii*^12^, have been recognized as plausible members of the group. Nevertheless, such a view on *B. cereus s.l.* species is a very simplified one, since their classification rely mainly on distinctive phenotypic traits, such as pathogenic potential to mammals (*B. anthracis* and *B. cereus* emetic or diarrheal strains) and insects (*B. thuringiensis*), physiology, e.g. psychro- (*B. weihenstephanensis*) or thermotolerance (*B. cytotoxicus*), as well as colony morphology (*B. mycoides* and *B. pseudomycoides*). Thus, although such approach is practical, it is not necessarily consistent with the group’s phylogenetic classification^13^,^14^,^15^. In addition, a number of species-specific features, e.g., anthrax, insecticidal and emetic toxins, are of plasmid origin^15^,^16^, and the discovery of *B. anthracis* plasmids in other *B. cereus s.l.* species makes their classification troublesome^17^. Therefore, several studies suggested that the *B. cereus* group should be considered as a single evolutionary unit characterized by clonal expansion and adaptation to various hosts and/or environments that possibly led to the formation of distinct phenotypes, also called ecotypes, within major phylogenetic lineages^14^,^18^,^19^.

Adaptation to various growth temperatures seems to be an important factor of the *B. cereus s.l.* diversification, as several thermotypes concurrent with phylogenetic groups I–VII were recognized^20^. The thermal growth limits of these bacilli are ranged from 5 to 50 °C. The most thermotolerant group VII (20–50 °C) is basal to the mesophilic group I (10–43 °C) from which the highly mesophilic group III (15–45 °C), mesophilic group IV (10–45 °C), mesophilic-psychrotolerant intermediary group V (8–40 °C), as well as two psychrotolerant groups, II (7–40 °C) and VI (5–37 °C), have emerged^20^,^21^. From this perspective, the evolution of *B. cereus s.l.* appears to shift from a thermotolerant to a mesophilic status, and to psychrotolerance^20^. Indeed, so-called psychrotolerance motifs were identified in chromosomes of psychrotolerant bacilli, i.e. in the *cspA* and *16S rRNA* genes^4^,^22^. Moreover, a link between the ∆5 desaturase (DesA)-dependent composition of fatty acids in a cell membrane and the ability to grow at low temperatures, has recently been established in certain members of the *B. cereus* group^21^.

Ribosomal proteins (r-proteins), being essential and conservative components of bacterial ribosomes, are an ideal target for bacteria identification and research on phylogenetic relationships. Hence, the multi-locus sequence typing based on genes encoding r-proteins (rMLST) was proposed as a universal method to characterize the phylogenetic position, from a domain to a strain, of any bacteria^23^. Accordingly, Tamura and co-workers^24^ developed a bacteria typing method based on r-proteins mass spectra^25^,^26^. In line with this, we previously showed that the mass variation in certain r-proteins enables differentiation of emetic *B. cereus* strains from other *B. cereus s.l.*^27^.

On the other hand, ribosomes of thermophilic, mesophilic and psychrophilic bacteria show specificity with regard to the temperature stability or amino acid composition^28^. Additionally, Pedone *et al*.^29^ demonstrated that thermophilic ribosomes are generally nonfunctional at low temperatures as the result of an enhanced conformational rigidity. However, what is even more intriguing, a thermosensory function of ribosomes for both the heat and cold shock response, was postulated^30^,^31^. Indeed, the relationship between r-proteins and temperature was indicated in several studies^32^,^33^,^34^. Hence, we assumed that differences between the *B. cereus* group thermotypes are reflected at the r-proteins level. Thus, the aim of the study was to reconstruct the population architecture of the *B. cereus* group based on a bioinformatical comparative study of r-proteins, in order to find a potential link with the thermotypes. The results were supported by the analysis of cold-shock proteins (CSPs) as well as screening of genomes for the presence of other elements involved in the temperature stress response, such as heat-shock proteins or desaturases.

## Results

### Ribosomal clusters (r-clusters) and their relation with the phylogenetic/thermal groups I–VII

**Figure 1:**
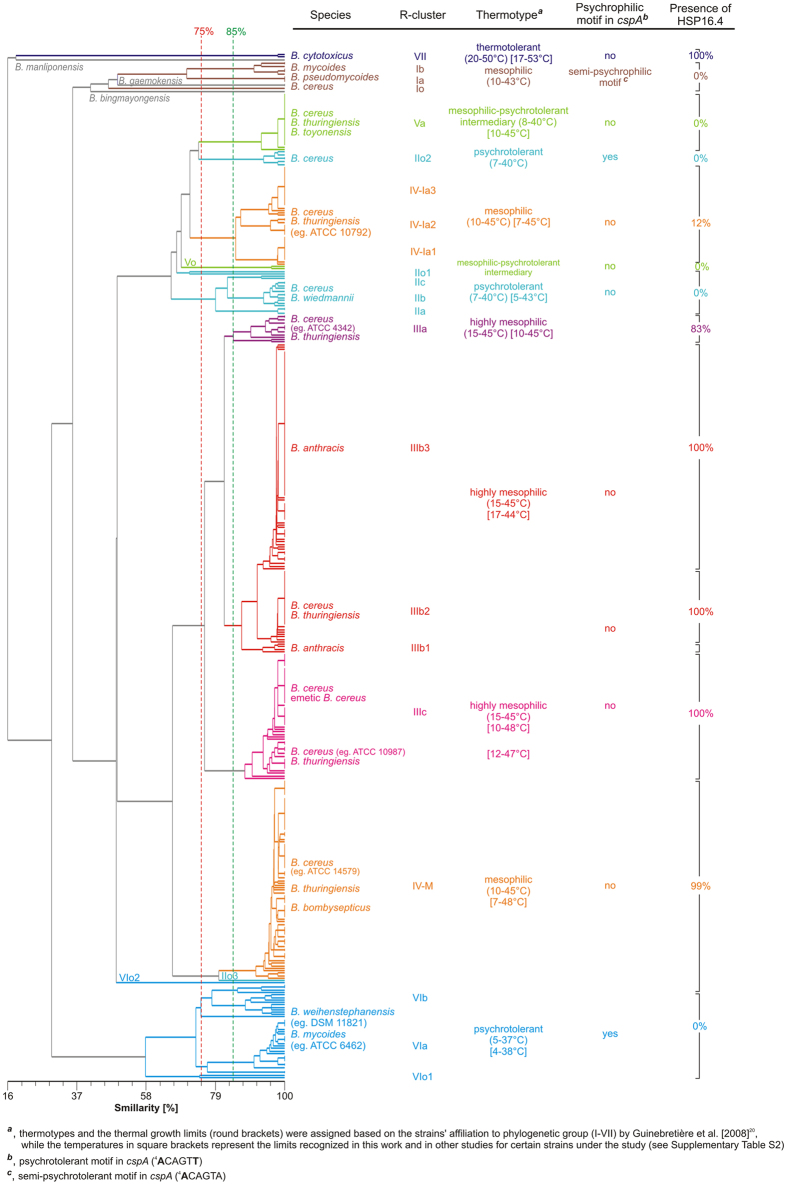
Phyloproteomic tree of the *B. cereus* group members constructed based on the r-protein variants.

In general, clustering of the bacilli based on their r-proteins variants (Fig. 1 and Supplementary Fig. S1) as well as amino acids sequences (Supplementary Fig. S2) created r-clusters that clearly correspond with their phylogenetic affiliation to the group from I to VII according to Guinebretière *et al*.^20^ as well as sequence types (STs) assigned based on the sequences of seven house-keeping genes (Supplementary Table S1). Hence, we adopted the phylogenetic groups denotations I–VII for the r-clusters, based on the Fig. 1 as the reference, by adding a lowercase letter suffix (a–c) and a digit (1–3) for r-subclusters with similarity of 75–85% and >85%, respectively. The ‘o’ suffix (that stands for outstanding) was reserved for strains outstanding from their native r-clusters (Fig. 1 and Supplementary Fig. S1). The detailed strains’ characteristics was included in Supplementary Table S1.

Three remarkable observations were made. Firstly, a high number of unique r-protein variants in the most psychrotolerant r-cluster VI resulted in its location on the tree immediately after the basal thermotolerant r-cluster VII (Fig. 1 and Supplementary Fig. S1) instead clustering it with the remaining psychrotolerant bacilli, as in the case of the tree built based on identity of r-proteins amino acid sequences (Supplementary Fig. S2). Secondly, the bacilli from the mesophilic group IV were split into two separate r-clusters, denoted as IV-M (M, presumed as mesophilic) and IV-I (I, presumed as intermediate) (Fig. 1, Supplementary Fig. S1 and Fig. S2). While the former r-cluster showed a closer relation with the highly mesophilic r-cluster III, the latter was intermixed with the r-clusters representing the mesophilic-psychrotolerant intermediary group V and the psychrotolerant group II. Thus, we presumed its thermal status as intermediate, that was partly supported by analysis of their thermal growth limits and available literature data (Supplementary Table S2). Similarly, certain strains affiliated to the psychrotolerant group II and the intermediate group V formed outstanding clusters, that could be distinguished from their native r-clusters, respectively, by the presence of the psychrotolerant motif in *cspA* and a lack of L33-2 r-protein. The remaining outstanding r-clusters were in general composed of single or two isolates without a common characteristic, and they were omitted in further comparative analyses. Thus, beside r-clusters VII, VI and I, the tree was composed of two major branches, the first one occupied by the psychrotolerant r-clusters II, V, and IV-I, and the second one with the mesophilic r-clusters III and IV-M (Fig. 1, Supplementary Fig. S1).

In addition, *B. manliponensis* and the group VII were basal to all r-clusters, whereas *B. bingmayongensis* and *B. gaemokensis* showed the closest relatedness to the group I. However, these species could not be affiliated to any phylogenetic group. On the other hand, *B. bombysepticus* and *Bacillus* sp. 7_6_55CFAA_CT2, both assigned to the r-cluster IV-I, and *B. toyonensis* (r-cluster V) were in fact indistinguishable from the other strains with the same r-clusters (Supplementary Fig. S1). Finally, *B. wiedmanii* was intermixed with strains from the r-cluster IIb.

### Features of the r-clusters

**Figure 2:**
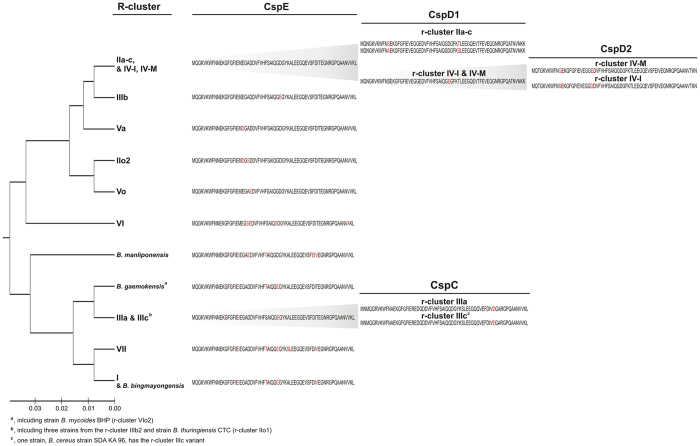
Application of cold shock proteins (CSPs) to discriminate the r-clusters of the *B. cereus* group thermotypes. The tree was built using the UPGMA method with MEGA7 software. The variable sites in CSPs were highlighted in red.

A distinctiveness of the r-clusters was generally confirmed by differences in their CSPs patterns (Supplementary Table S3, Supplementary Fig. S3 and Fig. S4) or even in the amino sequence of one of them, CspE (Fig. 2 and Supplementary Fig. S5). In addition, our analysis revealed a lack of L33-2 (Supplementary Table S1 and Fig. S6), HSP16.4 (Fig. 1, Supplementary Table S1 and Fig. S7) and DesA (Supplementary Table S1) proteins in certain r-clusters. Among the other heat-shock induced proteins under study only a lack of RNA polymerase sigma-B and anti-sigma-B factors (encoded by one operon) was observed in the r-clusters VII and I as well as a subset of psychrotolerant bacilli from r-clusters II, IV-I and VI (Supplementary Table S1 and Fig. S8). The characteristic of the basal and thermotolerant r-cluster VII involved the presence of (i) the semi-psychrotolerant motif (^4^**A**CAGTA) in *cspA,* (ii) the mesophilic motif in *16S rRNA* as well as (iii) L33-2, (iv) HSP16.4, and (v) a lack of DesA. In contrast, a lack of HSP16.4 was observed in strains from (i) the mesophilic r-cluster I, (ii) the intermediary and the psychrotolerant r-clusters (II, V) as well as (iii) the majority (88%) of the strains from the r-cluster IV-I. In addition, (i) the presence of DesA and/or (ii) the psychrotolerant/mesophilic motifs in *cspA* and *16S rRNA* as well as (iii) a lack of L33-2 were recognized in the r-clusters II–VI. In detail, the presence of the psychrotolerant motifs in *cspA* and/or *16S rRNA* along with a lack of L33-2 are characteristic for the highly psychrotolerant r-cluster VI, and with the exception of the psychrotolerant *16S rRNA* signature, also for the r-cluster IIo1. Finally, we noted that DesA in the predominant number (92%) of *B. anthracis* strains is 25 or 92 amino acids shorter (depending on the annotated next start codon) as the result of frameshift mutation, i.e. adenine deletion at nucleotide 27 of *desA*, that is rarely present in the remaining bacilli (Supplementary Fig. S9).

Furthermore, based on the observation that L33-2 r-protein is in general absent only in the psychrotolerant bacilli equipped with the ‘*cspA*’ motif (Supplementary Fig. S6) we developed a PCR reaction for their rapid identification that was successfully tested *in vitro* on 95 *B. cereus s.l.* isolates (Supplementary Table S4 and S5).

### R-proteins and amino acid composition

**Figure 3:**
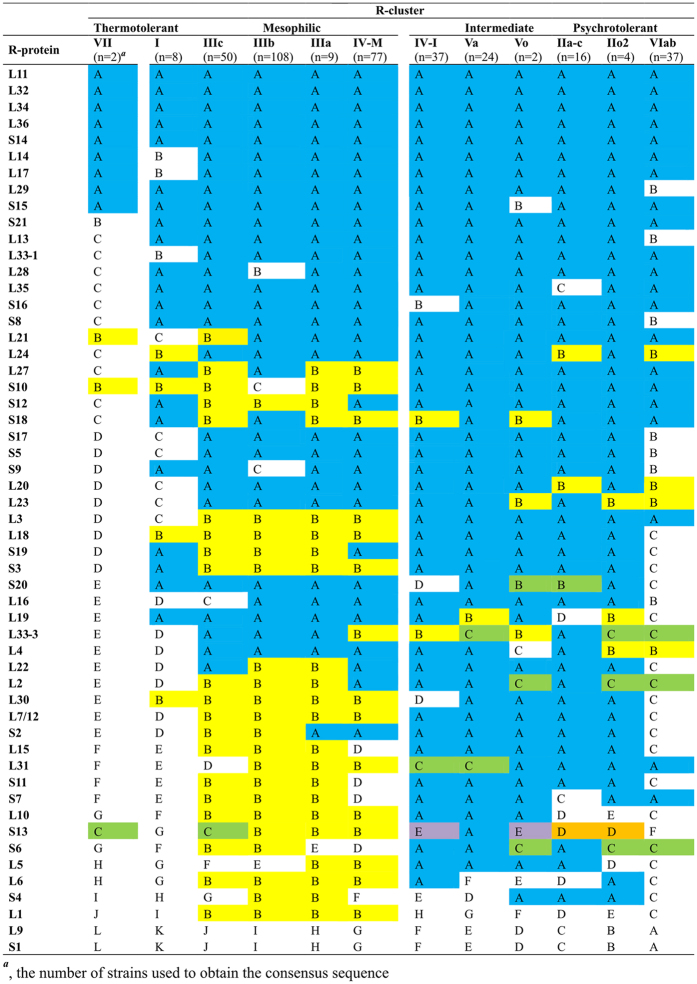
Comparison of the r-protein variants among the r-clusters of the *B. cereus* group thermotypes. The r-proteins variants were estimated based on alignment of the r-clusters’ consensus sequences. A combination of letter (A–L) and colour represents a r-protein variant shared by at least two r-clusters, white colour was reserved for single variants of the r-proteins.

In total, we recorded 603 variants among the 55 r-proteins under study (Supplementary Table S6). However, it must be pointed out that the majority of this enormous diversity may be ascribed to single-alleles (38% of all alleles). A variation ranged from generally invariant r-proteins, for example L11, L32, L34, S14, L32 and L36, to highly variable ones, such as S1, L1, L9, L5, L6 or S4, possessing at least 20 alleles (Fig. 3, Supplementary Table S6 and Fig. S10).

**Figure 4:**
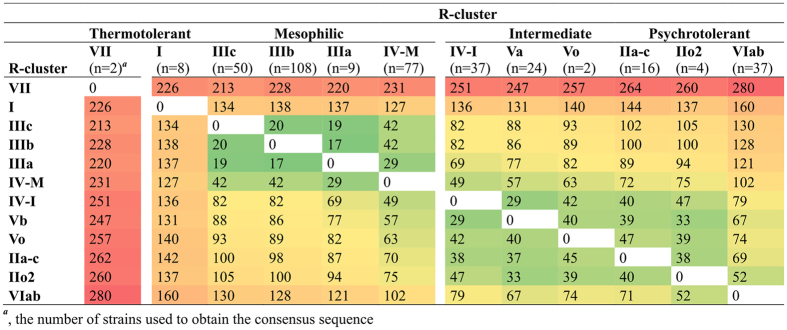
Number of differences in consensus sequences of concatenated 54 r-proteins (6808 amino acids) among the r-clusters of the *B. cereus* group thermotypes.

As aforementioned, only L33-2 was not present in all bacilli (Supplementary Table S1 and Fig. S6). Noteworthy is also the presence of the common alleles of L31, L33-3, L20 among the psychrotolerant r-clusters VI and II, which share also L3, S10, S7 variants with the intermediate r-cluster V as well as the r-cluster IV-I (Fig. 3 and Supplementary Table S6). Likewise, the thermotolerant r-cluster VII and the highly mesophilic r-cluster IIIc, predominantly occupied by emetic *B. cereus* strains, shared L21 and S13 alleles as well as the smallest number of amino acids differences in r-proteins (Fig. 4).

In general, a number of amino acid replacements in the concatenated sequences of 54 r-proteins (6808 amino acids) was well correlated with thermal characteristics of the r-clusters (Fig. 4), and reached 280 in between the extreme thermotypes. For example, the mesophilic branch, i.e. the r-clusters III and IV-M, was separated from the strains classified as intermediary psychrotrophic-mesophilic or psychrotolerant (even excluding the highly psychrotolerant r-cluster VI) by average 52 amino acid substitutions. However, surprisingly, the mesophilic r-cluster I showed almost equal number of differences, 134 *vs* 137 amino acid replacements, in comparison to these two groups. Furthermore, although an average number of amino acids differences between the highly psychrotolerant r-cluster VI and the remaining 11 r-clusters in comparison to this value for the r-cluster I (115 *vs* 146) is significantly smaller (p < 0.05, Mann-Whitney U two-tailed test). The amino acids substitutions are dispersed over relatively larger number of r-proteins in the r-cluster VI, which explains its unexpected location in the Fig. 1.

Finally, we noted a decrease of Alanine (A) and Valine (V) related with an increase of Serine (S) and Isoleucine (I) in the psychrotolerant r-clusters (Supplementary Table S7), that largely resulted from substitutions S → A and I → V in the mesophilic ones (Supplementary Table S8–S10). When expressed as the ratio (A + V)/(I + S), it ranged from 1.82 in the thermotolerant r-cluster VII, through, on average 1.80 in the mesophilic to 1.75 in the intermediate/psychrotolerant branches, and 1.73 in the highly psychrotolerant r-cluster VI.

## Discussion

Taking into consideration multiple relationships between temperature and ribosomes^28^,^31^,^34^, we performed a comparative bioinformatical study of the *B. cereus s.l.* r-proteins to establish a link between thermotolerant, mesophilic and psychrotolerant characteristics of the bacilli. Indeed, our results revealed a an association between alterations in the r-proteins and the *B. cereus s.l.* thermotypes that may be connected with a temperature-related adaptive trait, regardless of the phylogenetic position of the strains. For example, a subset of strains from the mesophilic phylogenetic group IV showed a closer relation with the intermediate/psychrotolerant thermotypes than with the mesophilic ones. In addition, we recognized differences in amino acids composition in r-proteins of mesophilic and psychrotolerant bacilli. Furthermore, surprisingly high number of unique r-protein variants as well as a lack of L33-2 r-protein were identified in the most psychrotolerant group VI. Finally, we suggested that a transition from the thermotolerance to a mesophilic status occurred independently twice in the evolutionary history of *B. cereus s.l.* These observations were further supported by the analysis of various proteins implicated with a response to temperature stress such as CSPs, HSP16.4 or DesA. On this ground, we proposed a scenario of the genetic events associated with an emergence of particular *B. cereu*s *s.l.* thermotypes (Fig. S11). Finally, it should be stated that due to limited availability of experimental data, the thermal growth limits were automatically assumed based on strains’ affiliation to the phylogenetic/thermal group^20^.

A connection between temperature and r-proteins was indicated by several studies. For example, r-proteins S6, L7/L12 and L10, are, among others, cold shock-induced proteins in *Bacillus subtilis*, and probably are implicated in the adaptation of ribosome function to low temperature^35^. Certainly, some r-proteins, in particular L22, L23 and L34, might play an important role in ribosomal assembly at lower temperatures in this bacterium^34^. Whereas, the deletion of genes encoding any of L1, L22, L23, L34, L36, S6 or S21 r-proteins in *B. subtilis* results in slower growth at 32 °C, without significant depression at 45 °C. Likewise, the deletion of S9, S11, S21, L4, L15, L27 or L2, L3, L5, L24, L28, L33, S13, S17, S20 in *Escherichia coli* generates cold-sensitive or temperature-sensitive mutants^36^. In addition, even a single amino acid substitution in L22^37^, L24^38^ or S5^33^ causes the high temperature- (42 °C) or cold-sensitivity (20 °C) in *E. coli*. Finally, ribosomal changes resulting from the cold shock were indicated as responsible for the decrease of the thermal tolerance in *Listeria monocytogenes*^32^. As the asymmetrical preference for certain amino acids over others in microorganisms growing at different temperatures is considered as an example of adaptive evolution^39^, one can imagine that the exposition to lower temperatures may ultimately force ‘cold-adaptive’ changes in r-proteins and other proteins. In fact, we noted that in the psychrotolerant *B. cereus s.l.* thermotypes Isoleucine and Serine are favored over Valine and Alanine, respectively. A preference for Isoleucine and an avoidance of Alanine was also observed in membrane proteins of psychrophilic Vibrionaceae, possibly as modification that increases their flexibility^40^. Thus, an advanced adaptation to cold in the most psychrotolerant thermotype VI appears to explain the observed discrepancy between unproportionally high number of unique r-proteins variants to their overall sequences identity in comparison to less psychrotolerant bacilli. Furthermore, the common variants of certain r-proteins, eg., L31, L33-3 or L20 shared by phylogenetically separated psychrotolerant bacilli as well as the detection of the thermotolerant-specific alleles of L21 and S13 r-proteins among the highly mesophilic emetic strains, could be interpreted as a positive temperature-based natural selection.

However, this mechanism does not explain why the *B. cereus s.l.* r-proteins of the mesophilic r-cluster I share a similar number of amino acid differences with those from the other mesophilic bacilli (r-cluster III and IV-M) as well as the intermediate or psychrotolerant ones (Fig. 4). While the former and the latter group are clearly distinct in this matter, in our opinion, such discrepancy imposes an independent evolution of two mesophilic lineages, possibly by mechanism known as a relaxation constraint, i.e. in this case relaxation from the selection to grow at high temperatures of their thermotolerant ancestor. According to this phenomenon, sites in proteins are constrained to have one amino acid in the ancestral species but could have more than one adaptively equivalent amino acid in descendant lineages^39^. Thus, it is reasonable that the ancestors of the r-cluster I and the remaining r-clusters, that in turn diversified into mesophilic and psychrotolerant lineages, ‘chosen’ different equivalent amino acids. Indeed, an alignment of the r-proteins sequences from those r-clusters revealed distinct amino acids at the same sites in comparison to the ancestral r-cluster VII (Supplementary Fig. S12). Certainly other evolutionary mechanisms also contributed to this process, such as recently described a regulatory divergence that shapes the expression of both the unique and shared proteins, resulting in phenotypic differentiation among the *B. cereus* group members^41^.

So far, an association with psychrotolerance in the *B. cereus* group was established for specific sequence motifs in *16S rRNA* and *cspA* of *B. weihenstephanensis*^4^,^22^. The authors suggested that *16S rRNA* exists in two different states and it may alter the properties of the 30S subunit translation depending on a temperature^22^. On the other hand, the role of the ‘*cspA*’ motif was not indicated. Previously, we noted that the psychrotolerant motif in *cspA* (^4^**A**CAGT**T**), considered as specific for the group VI^20^, is also present in certain strains affiliated to the group II^27^, denoted here as the r-cluster IIo1. However, the *16S rRNA* psychrotolerance motifs are not common trait in these bacilli (Supplementary Table S1). According to Liu and colleagues^42^, these strains show greater genetic similarity with the group VI than with other strains of the group II. Furthermore, in the basal thermotolerant group VII and the mesophilic group I we recognized the semi-psychrotolerant *cspA* motif (^4^**A**CAGT**A**), an equivalent of the psychrotolerant one at amino acid level. Thus, it is conceivable that the psychrotolerant signature in *cspA* may be simply a derivative of closer phylogenetic relation between these four r-clusters^42^ rather than the true adaptation to cold. On the contrary, a lack of L33-2 r-protein, restricted to the r-clusters VI and IIo1, appears to be a psychrotolerance-related trait, but certainly it must be verified experimentally.

Nevertheless, we noted that the patterns of six CSPs clearly correspond with the r-clusters, except the r-cluster IV-I. In the latter only a minor fraction of strains clustered consistently with their r-proteins background, i.e. together with the intermediate and psychrotolerant bacilli, while the remaining were adjacent to the r-cluster IV-M. Noteworthy, the growth limits of one of such strains, *B. cereus* RIVM BC 964 (7–42 °C)^43^, as well as certain other isolates support the thesis that the thermal tolerance of bacilli from the r-cluster IV-I is lower than those from the r-cluster IV-M.

In line with this, we recognized another common denominator for the r-cluster IV-I and all psychrotolerant or intermediate bacilli, namely a lack of the heat-shock induced protein HSP16.4. Surprisingly, the *hsp16.4* gene is adjacent to the operon encoding two-component system CasK/R which plays a role in the *B. cereus* group cold adaptation^44^. The substantial depression of the major GroEL and DnaK heat shock proteins’ synthesis was observed in *E. coli* after the growth under lower temperature^45^. Similar effect was exerted by antibiotics that mimic the cold shock^30^. Proceeding from this assumption, HSP16.4 may be redundant for psychrotolerant bacilli. Along with this suggestion comes an observation that 35% strains from the most psychrotolerant r-cluster VI as well as certain isolates from the r-clusters IIo1 and IV-I are devoid of two other heat-shock induced proteins encoded by one operon, namely RNA polymerase sigma-B and anti-sigma-B factors (Supplementary Fig. S8). However, it should be noted that this operon is also absent in the thermotolerant r-cluster VII and the mesophilic r-cluster I.

Beside of bacilli affiliated to the group IV, a variation in thermal growth limits may concern also the highly mesophilic group III (r-cluster III). *B. anthracis* (r-subcluster IIIb1 and IIIb3) generally cannot grow above 43–44 °C^46^,^47^, whereas emetic *B. cereus* strains (r-cluster IIIc) are thermophilic and have the ability to grow at 48 °C^43^. Partially it may be attributed to unique r-proteins, such as S13 and L21 or CSPs, eg., CspE, that is also induced in response to the heat-shock^48^. In addition, we observed that in the majority (92%) of *B. anthracis* strains DesA (∆5 desaturase) is truncated as the result of the frameshift mutation. Recently, the presence of DesA and differences in the amount of C16:1(5) unsaturated fatty acids at position ∆5 (∆5 UFAs), were suggested as an advanced mechanism of adaptation resulting in transition from mesophilic status to psychrotolerance in the groups II–VI^21^,^49^. DesA seems to play a crucial role in this process, since it is absent in groups I and VII which have only ∆10 desaturase (DesB)^21^,^49^ that is active regardless of the growth temperature, while the ∆5 desaturation system is upregulated at lower temperature^50^. Nevertheless, in contrast to the group I or VII, the amount of ∆5 UFAs, at least i:17(5) in *B. anthracis* is similar to that present in *B. cereus* or *B. thuringiensis*^51^. On the other hand, *B. anthracis* does not produce i17:1(10)^51^,^52^, the product of DesB, despite the fact that the *desB* gene is complete.

Another noteworthy observation regarding a majority of *B. anthracis* strains (r-cluster IIIb3) is a greater similarity of their r-protein patterns with those from certain *B. cereus* and *B. thuringiensis* isolates (r-cluster IIIb2), instead of *B. anthracis* belonging to ST933 (r-cluster IIIb1), i.e. representing the divergent C branch in *B. anthracis* phylogeny^53^. In detail, the difference between the r-clusters IIIb2 and IIIb3 concerns four r-proteins (L5, S10, L28, and S9). Interestingly, the pairs L5-S10 and L28-S9 are common for the r-cluster IIIb1 and the r-cluster IIIb2 and IIIb3, respectively. This supports the thesis that *B. anthracis* has recently diverged from a cluster of *B. cereus* and *B. thuringiensis* strains^13^,^14^,^54^.

Summing up, several observations support our hypothesis that an adaptation of *B. cereus s.l.* to various thermal niches is linked with adaptive alterations in r-proteins. In addition, we propose two major mechanisms behind this process, namely a relaxation constraint and a positive temperature-related natural selection. The former was responsible for an emergence of two distinct mesophilic lineages. While the latter resulted in a gradual increase in certain amino acids in r-proteins toward growing psychrotolerance as well as a selection of thermotype-specific r-proteins variants. Finally, we suggest that the observed divergence in r-proteins among strains from certain phylogenetic groups, e.g. the group IV, may be interpreted as the result of their diversification to various growth requirements and deserves on further investigations.

## Materials and Methods

### Characteristic of strains

A total of 421 non-repeatable complete and draft genomes of *B. anthracis* (n = 98), *B. cereus* (n = 232), *B. thuringiensis* (n = 64), *B. weihenstephanensis* (n = 6), *B. mycoides* (n = 10), *B. pseudomycoides* (n = 1), *B. toyonensis* (n = 1), *B. cytotoxicus* (n = 2), *B. manliponensis* (n = 1), *B. gaemokensis* (n = 2), *B. bombysepticus* (n = 1), *B. bingmayongensis* (n = 1), *B. wiedmannii* (n = 1) and *Bacillus* sp. 7_6_55CFAA_CT2 (n = 1), deposited in the Pathosystems Resource Integration Center (PATRIC) database (https://www.patricbrc.org; status from February, 2016), were used for *in silico* genomic and proteomic assays (Supplementary Table S1).

All strains were classified into seven phylogenetic groups I–VII, based on data collected in the *Bacillus cereus* group Typing Database (mlstoslo.uio.no) or using the web-tool (https://www.tools.symprevius.org/Bcereus/)^55^. Strains were also characterized in terms of ST types using MLST 1.8 web-based tool^56^, and categorized into groups based on the similarity in allelic profiles (defined here as the groups sharing at least three of the seven alleles) with eBURST software^57^, and denoted as eBURST groups.

In addition, the bacilli were screened for psychrotolerant signatures in *cspA* (^4^**A**CAGT**T**) and *16S rRNA* (^180^AA**T**ATTTTGAAC**T**GCAT**A**GTTC and ^1008^**T**CTAGAGATAG**A**) genes^4^,^22^. Furthermore, emetic *B. cereus* strains were identified by searching the cereulide synthetase genes (*cesB* and *cesD*). The analyses were performed with the CLC Genomics software (CLC Bio).

### Phyloproteomic analysis of ribosomal and cold shock proteins

The BLAST analysis (blastp algorithm, word size 3, e-value 1) was performed to determine polymorphisms in 55 r-proteins, including 34 large and 21 small subunit ones, with the CLC Genomics software (CLC Bio) (Supplementary Table S6). Subsequently, the r-proteins alleles were recorded and combined for all strains in binary form as 1 (present) and 0 (absent). The phyloproteomic tree was built using Dice coefficient and UPGMA clustering with NTSYSpc software ver. 2.1 (Exeter Software). The clusters of bacilli with similar patterns of r-proteins were denoted as ribosomal clusters (r-clusters). Concurrently, we compared amino acids sequences of the r-proteins using MEGA7 software^58^. Compatibility of the strains’ affiliation to particular r-clusters with their genetic relatedness, i.e. according to the digital DND-DNA hybridization (dDDH) method, was confirmed by comparison of *pycA* gene^42^ (Supplementary Fig. S13).

The entire procedure was reproduced for six cold shock proteins (CSPs), CspA, CspB, CspE, CspC, CspD1 and CspD2 (Supplementary Table S3).

### Detection of heat-shock induced proteins and desaturases

All strains were screened using BLAST for the presence of 18 heat-shock induced proteins (DnaK, GroEL, YbbT, AldA, MreB, FolD, Dra, SodA, ClpP, HSP16.4, PpiB, YflT, RsbV, SpoVG, TrxA, YloH, CspE, CspB) recognized in *B. cereus* ATCC 14579 by Periago *et al*.^48^ as well as two desaturases (DesA and DesB)^50^.

### PCRs

Two PCR reactions were used to discriminate the highly psychrotolerant bacilli from the r-cluster VIab from the other bacilli. To that end, primers which amplify (i) a 106 bp fragment of gene encoding for L33-2 r-protein, which is absent in the former bacilli (5′-GTTACTCGCTTTAATCGTGGAC-3′ and 5′-CTTGTACRGAGTGCGGNRAT-3′), and (ii) specific for these bacilli a 198 bp segment of DNA from the region where this gene is deleted (5′-GCAAGCAGCAAATGATGAGA-3′ and 5′-CAAAACCAAAAVYCATTADCAA-3′). The primers were designed using Primer3web^59^. PCR was performed in 50 μL volumes containing: 1x PCR buffer, 200 mM dNTP, 1 U Taq polymerase (Thermo Scientific), 20 pmol of each primer, and 5 μL of bacterial DNA. The PCR cycle conditions for the first reaction were as follows: 95 °C for 5 min, followed by 30 cycles of 95 °C for 30 s, 58 °C for 30 s and 72 °C for 30 s, and a final extension at 72 °C for 7 min. The second reactions was performed under identical PCR conditions except for the annealing temperature (56 °C). Amplification was conducted in a Veriti Thermal Cycler (Life Technologies). PCR products were visualized by electrophoresis on a 2% agarose gel stained with Midori Green Advance DNA Stain (Nippon Genetics) and documented by Gel Doc XR + System (Bio-Rad). References to additional analyses included in the Supplementary Information^60^,^61^,^62^.

## Additional Information

**How to cite this article:** Fiedoruk, K. *et al*. Ribosomal background of the *Bacillus cereus* group thermotypes. *Sci. Rep.* **7**, 46430; doi: 10.1038/srep46430 (2017).

**Publisher's note:** Springer Nature remains neutral with regard to jurisdictional claims in published maps and institutional affiliations.

## Supplementary Material

Supplementary Information
