# Supplementary material for: Ribosomal background of the Bacillus cereus group thermotypes
Source: Sci Rep. 2017 Apr 13;7:46430. doi: 10.1038/srep46430 (PMC5390287; doi:10.1038/srep46430)
Supplement: Supplementary Information [file srep46430-s1.pdf]

Ribosomal background of the *Bacillus cereus* group thermotypes

Krzysztof Fiedoruk[1,*], Justyna M. Drewnowska[2], Tamara Daniluk[1], Katarzyna Leszczynska[1], Piotr Iwaniuk[2] & Izabela Swiecicka[2, 3]

[1]Department of Microbiology, Medical University of Bialystok, Bialystok, Poland.
[2]Department of Microbiology, Institute of Biology, University of Bialystok, Bialystok, Poland.
[3]Laboratory of Applied Microbiology, University of Bialystok, Bialystok, Poland.
*Corresponding author, email: krzysztof.fiedoruk@umb.edu.pl

Supplementary Information

**Table S1**	The *B. cereus* group strains used in this study.
**Table S2**	Temperature growth limits for the *B. cereus s.l.* phylogenetic groups (I-VII) by Guinebretière et al. [2008][20] and their values experimentally estimated in this work or available in literature for certain strains under the study.
**Table S3**	The cold-shock protein (CSPs) variants in the *B. cereus* group.
**Table S4**	In silico PCR results for the *B. cereus* group members with the primers specific for (i) the gene encoding L33-2 r-protein and (ii) strains from the r-cluster VIab. The in silico PCR was performed with Simulate_pcr v1.2 software[62].
**Table S5**	In vitro PCR results for the *B. cereus* group members with the primers specific for (i) the gene encoding L33-2 r-protein and (ii) strains from the r-cluster VIab.
**Table S6**	The r-protein variants in the *B. cereus* group.
**Table S7**	Distribution of amino acids in the 54 r-proteins (6808 amino acids) among the r-clusters of the *B. cereus* group thermotypes.
**Table S8**	Prevalence of substitution of Isoleucine (I) for Valine (V) in r-proteins among the *B. cereus s.l.* r-clusters I, II, III, IV, V and VII in comparison to the most psychrotolerant r-cluster VI.
**Table S9**	Prevalence of substitution of Serine (S) for Adenine (A) in r-proteins among the *B. cereus s.l.* r-clusters I, II, III, IV, V and VII in comparison to the most psychrotolerant r-cluster VI.
**Table S10**	Prevalence of substitution of Serine (S) for Threonine (T) in r-proteins among the *B. cereus s.l.* r-clusters I, II, III, IV, V and VII in comparison to the most psychrotolerant r-cluster VI.
**Figure S1**	Phyloproteomic tree of the *B. cereus* group constructed based on the comparison of the 55 r-proteins using the Dice coefficient and UPGMA clustering with NTSYSpc 2.1 software. The analysis involved 421 strains.
**Figure S2**	Phyloproteomic tree of the *B. cereus* group prepared based on concatenated sequences of the 54 r-proteins (6808 amino acids) using the Neighbor-Joining with MEGA7 software. The analysis involved 383 strains, i.e. all strains with the complete amino acid sequences for the 54 r-proteins. The evolutionary distances were computed using the Poisson correction method.
**Figure S3**	Phyloproteomic tree of the *B. cereus* group built based on the comparison of the six CSPs using the Dice coefficient and UPGMA clustering with NTSYSpc 2.1 software. The analysis involved 421 strains.
**Figure S4**	Phyloproteomic tree of the *B. cereus* group prepared based on concatenated sequences of the six CSPs (398 amino acids) using the Neighbor-Joining with MEGA7 software. The analysis involved 407 strains, i.e. all strains with the complete amino acid sequences for the six CSPs. The evolutionary distances were computed using the Poisson correction method.
**Figure S5**	Phyloproteomic tree of the *B. cereus* group prepared based on sequence of CspE using the Neighbor-Joining with MEGA7 software. The analysis involved 420 strains, i.e. all strains with the complete amino acid sequence for the CspE. The evolutionary distances were computed using the Poisson correction method.
**Figure S6**	Genomic environment and prevalence of gene encoding for L33-2 r-protein among the r-clusters of the *B. cereus* group. The figure was prepared using Easyfig software[59].
**Figure S7**	Genomic environment and prevalence of gene encoding for HSP16.4 protein among the r-clusters of the *B. cereus* group. The figure was prepared using Easyfig software[59].
**Figure S8**	Genomic environment and prevalence of the operon encoding for RNA polymerase sigma-B and anti-sigma-B factors among the r-clusters of the *B. cereus* group. The figure was prepared using Easyfig software[59].
**Figure S9**	Frameshift mutation in *desA* among *B. anthracis* strains and the remaining species of the *B. cereus* group.
**Figure S10**	Number of large (A) and small (B) subunit r-protein variants among the *B. cereus* group. Black and gray colours represent, respectively, essential and non-essential r-proteins as designated by Akanuma et al. (2012)[34].
**Figure S11**	Comparison of genomic and r-proteins relatedness between the *B. cereus* group thermotypes and plausible genetic events in their evolution. The r-clusters with inconsistent genomic and r-proteins relatedness were highlighted in gray circles.
**Figure S12**	Number of amino acids replacements in the 54 r-proteins of the *B. cereus* group between the r-cluster VII (denoted as ancestor) and the r-clusters I (denoted as mesophilic lineage 1) as well as the r-clusters III (representing mesophilic branch) and Va (representing intermediary mesophilic-psychrotolerant branch) and collectively denoted as mesophilic lineage 2.<br>Replacements were identified based on an alignment of concatenated consensus sequences of 54 r-proteins (6808 amino acids) from the r-clusters.
**Figure S13**	Typing of the *B. cereus* group strains based on comparison of *pycA* gene. The analysis involved 420 strains, i.e. all strains with the complete *pycA* sequence, and was performed using the Neighbor-Joining with MEGA7 software. The evolutionary distances were computed using the Jukes-Cantor method.

Table S1. *B. cereus* group strains used in this study.

No.	Strain	R-cluster	Phylogenetic group	eBURST group	ST[a]	MLST alleles: glp	gmk	ilv	pta	pur	pyc	tpi	Psychrotrophic motif in cspA (ACAGTA)	Psychrotrophic motif in 16S rRNA (AATATTTTGAACTGCATAGTTC)	Psychrotrophic motif in 16S rRNA (TCTAGAGATAGA)	HSP16.4	DesA	L33-2	Anti-sigma b & RNA polymerase sigma-B factors	Isolation country and/or date	Source
1	*B. cereus* Rock3-44 | 526986.3	Ib	I	Singleton	Nd11	142	83	247	141	31	125	106	no	no	no	absent	absent	present	absent	USA, Maryland, Rockville	Soil
2	*B. cereus* VD136 | 1053234.3	Ia	I	13	940	109	18	120	24	20	30	173	semi-psychrotrophic motif (ACAGTA)	no	no	absent	absent	present	absent	UK, Scotland	Soil
3	*B. cereus* VDM006 | 1085379.3	Ia	I	13	Nd22	109	18	120	24	31	30	173	semi-psychrotrophic motif (ACAGTA)	no	no	absent	absent	present	absent	Spain	Soil
4	*B. cereus* VDM021 | 1085386.3	Ia	I	13	Nd23	109	18	120	24	31	30	175	semi-psychrotrophic motif (ACAGTA)	no	no	absent	absent	present	absent	Greenland	Soil
5	*B. cereus* Al Hakam | 1396.149	Ib	I	Singleton	260	106	59	58	88	99	78	66	semi-psychrotrophic motif (ACAGTA)	no	no	absent	absent	present	absent	Iraq	Other, suspected bioweapons facility
6	*B. mycoides* Flugge 10206 | 1405.9	Ib	I	15	259	98	13	84	84	84	73	74	semi-psychrotrophic motif (ACAGTA)	no	no	absent	absent	present	absent	No data	No data
7	*B. mycoides* Rock1-4 | 526998.3	Ib	I	15	969	134	13	84	84	84	73	74	semi-psychrotrophic motif (ACAGTA)	no	no	absent	absent	present	absent	USA, Maryland, Rockville	Soil
8	*B. mycoides* Rock3-17 | 526999.3	Ib	I	15	259	98	13	84	84	84	73	74	semi-psychrotrophic motif (ACAGTA)	no	no	absent	absent	present	absent	USA, Maryland, Rockville	Soil
9	*B. mycoides* strain 219298 | 1405.13	Ib	I	Singleton	260	106	59	58	88	99	78	66	semi-psychrotrophic motif (ACAGTA)	no	no	absent	absent	present	absent	No data	No data
10	*B. pseudomycoides* DSM 12442 | 527000.3	Ib	I	Singleton	83	63	13	58	23	25	44	35	semi-psychrotrophic motif (ACAGTA)	no	no	absent	absent	present	absent	Ghana or Sweden or USA	Soil
11	*B. cereus* BS-2 | 1085389.3	IIa	II	12	978	81	53	117	193	113	93	80	no	no	no	absent	present	present	present	China	Soil
12	*B. cereus* BAG2O-3 | 1053178.3	IIa	II	12	746	81	53	117	193	113	93	145	no	no	no	absent	present	present	present	USA, Massachusetts, Boston	Soil
13	*B. cereus* BAG3O-1 | 1085388.3	IIa	II	12	946	81	53	117	71	209	93	80	no	no	no	absent	present	present	present	USA, Massachusetts, Boston	Soil
14	*B. cereus* BAG2X1-2 | 1053180.3	IIb	II	7	976	59	68	192	48	42	128	26	no	no	no	absent	present	present	present	USA, Massachusetts, Boston	Soil
15	*B. cereus* BAG5X2-1 | 1053191.3	IIb	II	7	949	102	130	107	22	96	20	26	no	no	no	absent	present	present	present	USA, Massachusetts, Boston	Soil
16	*B. cereus* BAG6X1-1 | 1053194.3	IIb	II	7	733	59	68	192	48	42	101	26	no	no	no	absent	present	present	present	USA, Massachusetts, Boston	Soil
17	*B. cereus* MM3 | 526971.3	IIb	II	7	126	45	35	20	49	42	20	26	no	no	no	absent	present	present	present	USA (2003)	Food
18	*B. cereus* strain 14294-3 (M6) | 1396.441	IIb	II	7	630	102	35	168	22	96	20	78	no	no	no	absent	present	present	present	Germany(2004)	ice cream
19	*B. cereus* strain B4077 | 1396.428	IIb	II	7	Nd52	59	35	106	49	42	20	26	no	no	no	absent	present	present	present	Netherlands(2012)	chilled dessert
20	*B. cereus* strain B4147 | 1396.433	IIb	II	7	Nd61	102	34	111	48	52	20	26	no	no	no	absent	present	present	present	Netherlands(2012)	cereals, breads, pasta&pastries
21	*B. wiedmannii* strain FSL W8-0169 | 1396.500	IIb	II	7	Nd62	102	35	107	22	96	20	26	no	no	no	absent	present	present	present	USA: New York(2012)	Raw milk
22	*B. cereus* strain MHI 226 | 1396.452	IIb	II	Singleton	901	191	41	66	48	42	61	49	no	no	no	absent	present	present	present	Germany	Milk and milk products
23	*B. cereus* strain RIMV BC 126 | 1396.449	IIb	II	Singleton	Nd56	73	73	129	22	127	63	26	no	no	no	absent	present	present	present	unknown	Human, faeces/stools [diarrheal food poisoning]
24	*B. cereus* strain S612 | 1396.465	IIb	II	7	Nd72	174	35	75	22	42	20	26	no	no	no	absent	present	present	present	USA(2015)	groundwater
25	*B. cereus* AH1271 | 526992.3	IIc	II	Singleton	953	59	133	149	229	214	122	179	no	no	no	absent	present	present	present	Iceland, Reykjavik (2000)	Human, cervix; Reykjavik University Hospital [from the same hospital as isolates AH1271, AH1272, and AH1273]
26	*B. cereus* strain FSL K6-0069 | 1396.516	IIc	II	Singleton	Nd63	102	36	61	50	53	56	148	no	no	no	absent	present	present	present	USA: New York(2012)	Laboratory Heat Treated Raw Milk 2
27	*B. cereus* R309803 | 526968.3	IIo1	II	Singleton	74	42	27	37	41	40	42	32	no	no	no	absent	present	present	absent	UK, England (2003)	Human, blood [human septicaemia]
28	*B. thuringiensis* strain CTC | 1428.335	IIo1	II	Singleton	Nd64	102	5	6	3	113	154	12	no	no	no	present	present	present	present	China: Wuhan (1999)	soil
29	*B. cereus* AH1272 | 526993.3	IIo2	II	14	932	202	116	223	174	205	171	128	yes	no	no	absent	present	absent	present	Iceland, Reykjavik (2000)	Human, cervix; Reykjavik University Hospital [from the same hospital as isolates AH1271, AH1272, and AH1273]
30	*B. cereus* AH1273 | 526994.3	IIo2	II	14	932	202	116	223	174	205	171	128	yes	no	no	absent	present	absent	present	Iceland, Reykjavik (2000)	Human, cervix; Reykjavik University Hospital [from the same hospital as isolates AH1271, AH1272, and AH1273]
31	*B. cereus* BAG1O-1 | 1053167.3	IIo2	II	14	934	213	116	223	223	118	107	128	yes	no	no	absent	present	absent	absent	USA, Massachusetts, Boston	Soil
32	*B. cereus* BAG1X1-3 | 1053172.3	IIo2	II	14	934	213	116	223	223	118	107	128	yes	no	no	absent	present	absent	absent	USA, Massachusetts, Boston	Soil
33	*B. cereus* BAG3X2-1 | 1053183.3	IIo2	II	Singleton	Nd3	215	116	187	170	207	144	172	yes	no	yes (single copy)	absent	present	absent	absent	USA, Massachusetts, Boston	Soil
34	*B. cereus* VDM053 | 1053249.3	IIo2	II	14	Nd24	216	129	223	225	118	171	128	yes	no	yes (single copy)	absent	present	absent	absent	Belgium	Other, Water from a small pond site
35	*B. cereus* strain FS28-94 | 1396.440	IIo3	II	Singleton	Nd51	49	31	45	43	45	53	159	no	no	no	absent	present	present	present	UK (1994)	Chinese restaurant beef & chow mein & rice[food poisoning outbreak]
36	*B. cereus* 03BB87 | 1396.146	IIIa	III	9	78	24	22	33	37	34	38	5	no	no	no	absent	present	present	present	USA, Texas (2003)	Human, blood [human fatal pneumonia; carries pXO1 plasmid with anthrax virulence genes and produces a polysaccharide capsule]
37	*B. cereus* ATCC 4342 | 526977.3	IIIa	III	9	38	24	12	50	21	23	31	19	no	no	no	present	present	present	present	USA (1900)	Dairy products, milk [sensitive to gamma phage infecting B.anthracis; three complete genomes sequenced for this strain with identical MLST sequences]
38	*B. cereus* FM1 | 1396.147	IIIa	III	9	78	24	22	33	37	34	38	5	no	no	no	present	present	present	present	No data	No data
39	*B. cereus* strain BcFL2013 | 1396.419	IIIa	III	9	78	24	22	33	37	34	38	5	no	no	no	absent	present	present	present	USA(2013)	Human
40	*B. cereus* strain FSL K6-0067 | 1396.510	IIIa	III	9	Nd44	24	22	33	37	71	38	5	no	no	no	present	present	present	present	USA: New York(2012)	Laboratory Heat Treated Raw Milk
41	*B. cereus* strain FSL M8-0117 | 1396.513	IIIa	III	17	219	74	22	85	62	66	66	75	no	no	no	present	present	present	present	USA: New York(2012)	Laboratory Heat Treated Raw Milk
42	*B. cereus* strain FT9 | 1396.420	IIIa	III	9	Nd43	24	12	50	21	23	58	19	no	no	no	present	disrupted	present	present	Brazil, Caldas Novas, Goias	hot springs
43	*B. cereus* strain SDA KA 96 | 1396.457	IIIa	III	Singleton	Nd50	48	30	33	61	44	31	51	no	no	no	present	present	present	present	Sweden (1997)	Dairy products, raw milk
44	*B. thuringiensis* LM1212 | 1417985.4	IIIa	III	17	Nd29	74	22	118	115	66	66	52	no	no	no	present	present	present	present	Madagascar	Animal, Insect, beetle (Oryctes gigas) cadaver
45	*B. thuringiensis* ser. tochigiensis BGSC 4Y1 | 527024.3	IIIa	III	9	104	24	12	33	37	48	31	19	no	no	no	present	present	disrupted	present	Japan	Other, soil or litter of silkworm (Bombyx mori)
46	*B. thuringiensis* strain KB1 | 1428.341	IIIa	III	9	104	24	12	33	37	48	31	19	no	no	no	present	present	present	present	South Korea: Daejeon (2012)	Arabidopsis thaliana
47	*B. thuringiensis* XL6 | 1428.243	IIIa	III	9	104	24	12	33	37	48	31	19	no	no	no	disrupted	present	disrupted	present	China, Inner Mongolia	Soil
48	*B. anthracis* 2000031052 | 1392.63	IIIb1	III	2	933	65	1	1	1	1	1	1	no	no	no	present	present	present	present	USA, Wyoming (1956)	Animal, cattle
49	*B. anthracis* 2002013094 | 1392.77	IIIb1	III	2	933	65	1	1	1	1	1	1	no	no	no	present	present	present	present	USA (1956)	Soil. [pXO1+/pXO2+; two complete genomes sequenced for this strain with identical MLST sequences]
50	*B. anthracis* str. A1055 | 280355.4	IIIb1	III	2	933	65	1	1	1	1	1	1	no	no	no	present	present	present	present	USA, Louisiana	[belongs to the divergent C branch; pXO1-/pXO2+]
51	*B. anthracis* strain 2000031021 | 1392.231	IIIb1	III	2	933	65	1	1	1	1	1	1	no	no	no	present	present	present	present	USA, Louisiana	divergent C branch; pXO1-/pXO2+]
52	*B. anthracis* strain RIT375 | 1392.242	IIIb2	III	Singleton	76	34	15	28	38	30	29	33	no	no	no	present	present	present	present	Puerto Rico (2015)	Costus igneus
53	*B. cereus* | 1396.463	IIIb2	III	2	Nd54	65	1	1	1	1	37	42	no	no	no	present	disrupted	disrupted	present	Saudi Arabia	area around Jeddah
54	*B. cereus* 03BB102 | 572264.4	IIIb2	III	2	11	34	1	32	1	33	37	24	no	no	no	present	present	present	present	USA, Texas (2003)	Human, blood [human fatal pneumonia; carries pXO1 plasmid with anthrax virulence genes]
55	*B. cereus* 3a | 1396.148	IIIb2	III	2	145	65	1	52	1	1	37	24	no	no	no	present	present	present	present	USA, Wisconsin, Madison	Other, Food Research Institute, University of Wisconsin-Madison
56	*B. cereus* 95/8201 | 526979.3	IIIb2	III	2	145	65	1	52	1	1	37	24	no	no	no	present	present	present	present	UK, Wales, Merthyr Tydfil (1995)	Human, endocarditis; Prince Charles Hospital
57	*B. cereus* AH820 | 405535.8	IIIb2	III	2	460	65	1	56	1	1	53	24	no	no	no	present	present	present	present	Norway, Akershus (1995)	Human, periodontitis
58	*B. cereus* BGSC 6E1 | 526970.3	IIIb2	III	2	108	38	1	32	16	18	33	24	no	no	no	present	present	present	present	No data	[produces chitinase]
59	*B. cereus* biovar anthracis str. CI | 637380.6	IIIb2	III	2	935	34	1	83	1	18	29	5	no	no	no	present	present	present	present	Ivory Coast, Tai National Park (2001,2002)	Animal, great apes (chimpanzees) [previously named atypical B.anthracis because it carries pXO1 and pXO2 plasmids with anthrax virulence and polyglutamate capsule genes; produces a capsule]
60	*B. cereus* D17 | 1454382.3	IIIb2	III	2	Nd6	34	1	124	16	18	33	89	no	no	no	present	present	present	present	USA, Wisconsin, Madison	Other, Food Research Institute, University of Wisconsin-Madison
61	*B. cereus* E33L | 288681.15	IIIb2	III	Singleton	908	115	124	119	116	119	108	88	no	no	no	present	present	present	present	Namibia, Etosha National Park (1996)	Animal, Carcass of dead zebra [two complete genomes sequenced for this strain with identical MLST sequences]
[illegible]	[illegible]	[illegible]	[illegible]	[illegible]	[illegible]	[illegible]	[illegible]	[illegible]	[illegible]	[illegible]	[illegible]	[illegible]	[illegible]	[illegible]	[illegible]	[illegible]	[illegible]	[illegible]	[illegible]	[illegible]	[illegible]

Table S2. Temperature growth limits for the *B. cereus s.l.* phylogenetic groups (I-VII) by Guinebretière et al. [2008][20] and their values experimentally estimated in this work or available in literature for certain strains under the study.

Strain	Phylogenetic group	Temperature growth limits [°C]				R-cluster	ST[a]	References
		According to strain's affiliation to the phylogenetic group		Experimentally determined in other studies				
		Lower	Upper	Lower	Upper			
B. cereus F4430/73	IV	10	45	7	45	IV-M	142	43
B. cereus INRA A3	IV	10	45	>10	45	IV-M	Nd46	43
B. cereus INRA C3	IV	10	45	10	45	IV-M	395	43
B. cereus RIVM BC 934	IV	10	45	>10	48	IV-M	15	43
B. cereus ATCC 14579	IV	10	45	12	46	IV-M	4	60
B. cereus ATCC 14579	IV	10	45	>10	47	IV-M	4	this work [b]
B. thuringiensis IS5056	IV	10	45	>10	<45	IV-Ia1	10	this work [b]
B. thuringiensis 4Q7	IV	10	45	>10	<45	IV-Ia3	16	this work [b]
B. thuringiensis HD567	IV	10	45	>10	<45	IV-Ia3	16	this work [b]
B. cereus RIVM BC 964	IV	10	45	7	42	IV-Ia2	Nd69	43
B. anthracis str. Sterne	III	10	45	17	44	IIIb3	1	46
B. cereus F837/76	III	15	45	10	48	IIIb2	75	43
B. cereus SDA KA96	III	15	45	10	45	IIIa	Nd50	43
B. cereus ATCC 10987	III	15	45	12	47	IIIc	32	60
B. cereus NVH 0075-95	III	15	45	>10	48	IIIc	26	43
B. cereus RIVM BC 90	III	15	45	>10	48	IIIc	164	43
B. cereus NC7401 (emetic strain)	III	15	45	>10 (10)[c]	48	IIIc	26	43
B. toyonensis BCT-7112	V	8	40	10	45	V	111	5
B. weihenstephanensis WSBC 10204	VI	5	37	4	<40	VIa	196	4
B. mycoides ATCC 6462 (DSM 2048)	VI	5	37	7	<40	VIa	116	4
B. weihenstephanensis KBAB4	VI	5	37	8	38	VIb	958	60
B. wiedmannii FSL W8-0169 (other strains)	II	7	40	10 (5)	40 (43)	IIb	Nd62	12
B. cereus F528-94	II	7	40	7	45	IIo3	Nd51	43
B. cytotoxicus NVH 391-98	VII	20	50	17	53	VII	Nd26	60

[a], Nd, not described ST
[b], temperature growth limits were determined on LB agar plates after 48h of incubation at 7°C, 10°C, 45°C, 46°C, 47°C and 48°C
[c], 10°C was recognized as the lower limit for certain emetic strains

Table S3. The cold-shock protein (CSPs) variants in the *B. cereus* group.

CSP / Genbank Accession No.	CspA	CspA	CspA	CspA	CspA	CspA	CspA	CspA	CspA	CspA
Strain	WP_034637437.1	WP_000301518.1	WP_002011282.1	WP_000301519.1	WP_000301523.1	WP_033673878.1	WP_000301522.1	WP_000301520.1	WP_000218989.1	WP_061131525.1
B. cereus Al Hakam | 1396.149	0	0	0	0	0	0	0	0	0	0
B. mycoides strain 219298 | 1405.13	0	0	0	0	0	0	0	0	0	0
B. pseudomycoides DSM 12442 | 527000.3	0	0	0	0	0	0	0	0	0	0
B. mycoides Rock1-4 | 526998.3	0	0	0	0	0	0	0	0	0	0
B. mycoides Rock3-17 | 526999.3	0	0	0	0	0	0	0	0	0	0
B. mycoides Flugge 10206 | 1405.9	0	0	0	0	0	0	0	0	0	0
B. cereus VD136 | 1053234.3	0	0	0	0	0	0	0	0	0	0
B. cereus VDM006 | 1085379.3	0	0	0	0	0	0	0	0	0	0
B. cereus VDM021 | 1085386.3	0	0	0	0	0	0	0	0	0	0
B. gaemokensis strain JCM 15801 | 574375.6	0	0	0	0	0	1	0	0	0	0
B. gaemokensis strain KCTC 13318 | 574375.8	0	0	0	0	0	1	0	0	0	0
B. cereus Rock3-44 | 526986.3	0	0	0	0	0	0	1	0	0	0
B. bombysepticus str. Wang | 1330043.5	0	1	0	0	0	0	0	0	0	0
B. cereus strain F3175-03 | 1396.444	0	1	0	0	0	0	0	0	0	0
B. cereus strain LK9 | 1396.436	0	0	0	0	0	0	1	0	0	0
B. cereus VD166 | 1053240.3	0	0	0	0	0	0	1	0	0	0
B. cereus B4264 | 405532.5	0	1	0	0	0	0	0	0	0	0
B. cereus VD196 | 1053243.3	0	0	0	0	0	0	1	0	0	0
B. cereus BAG3O-2 | 1053182.3	0	1	0	0	0	0	0	0	0	0
B. cereus BAG4O-1 | 1053185.3	0	1	0	0	0	0	0	0	0	0
B. thuringiensis T01-328 | 1324966.3	0	1	0	0	0	0	0	0	0	0
B. cereus BAG4X12-1 | 1053186.3	0	1	0	0	0	0	0	0	0	0
B. cereus BAG5X12-1 | 1053190.3	0	1	0	0	0	0	0	0	0	0
B. cereus strain FSL W8-0640 | 1396.509	0	1	0	0	0	0	0	0	0	0
B. cereus F65185 | 526989.3	0	1	0	0	0	0	0	0	0	0
B. cereus BAG3X2-2 | 1053184.3	0	1	0	0	0	0	0	0	0	0
B. cereus VD133 | 1053233.3	0	1	0	0	0	0	0	0	0	0
B. cereus Rock4-2 | 526987.3	0	1	0	0	0	0	0	0	0	0
B. thuringiensis ser. galleriae HD-29 | 29338.3	0	1	0	0	0	0	0	0	0	0
B. thuringiensis strain BR37 | 1428.336	0	1	0	0	0	0	0	0	0	0
B. cereus strain 6/27/S | 1396.458	0	1	0	0	0	0	0	0	0	0
B. cereus strain B4158 | 1396.434	0	1	0	0	0	0	0	0	0	0
B. cereus strain RIVM BC 934 | 1396.456	0	1	0	0	0	0	0	0	0	0
B. thuringiensis ser. aizawai str. Hu4-2 | 1238885.4	0	1	0	0	0	0	0	0	0	0
B. thuringiensis ser. aizawai str. Leapi01 | 1232203.4	0	1	0	0	0	0	0	0	0	0
B. thuringiensis DB27 | 1431339.3	0	1	0	0	0	0	0	0	0	0
B. thuringiensis ser. huazhongensis BGSC 4BD1 | 527030.3	0	0	0	0	1	0	0	0	0	0
B. cereus ATCC 10876 | 526980.3	0	1	0	0	0	0	0	0	0	0
B. cereus strain FSL K6-0073 | 1396.511	0	1	0	0	0	0	0	0	0	0
B. cereus VD140 | 1053235.3	0	1	0	0	0	0	0	0	0	0
B. cereus VD156 | 1053239.3	0	0	0	0	1	0	0	0	0	0
B. thuringiensis BMB171 | 714359.3	0	0	0	0	0	0	1	0	0	0
B. cereus AH1134 | 405533.4	0	1	0	0	0	0	0	0	0	0
B. cereus strain FSL W8-0767 | 1396.506	0	1	0	0	0	0	0	0	0	0
B. cereus ISP2954 | 1053215.3	0	1	0	0	0	0	0	0	0	0
B. thuringiensis strain YWC2-8 | 1428.324	0	1	0	0	0	0	0	0	0	0
B. thuringiensis ser. kurstaki HD 1 | 29339.37	0	1	0	0	0	0	0	0	0	0
B. thuringiensis ser. kurstaki str. HD73 | 1279365.3	0	1	0	0	0	0	0	0	0	0
B. thuringiensis ser. kurstaki str. T03a001 | 527023.3	0	1	0	0	0	0	0	0	0	0
B. cereus BMG1.7 | 1053196.3	0	1	0	0	0	0	0	0	0	0
B. cereus HD73 | 1053200.3	0	1	0	0	0	0	0	0	0	0
B. cereus HuB13-1 | 1053208.3	0	1	0	0	0	0	0	0	0	0
B. thuringiensis AK47 | 1428.247	0	1	0	0	0	0	0	0	0	0
[illegible]										
B. cereus CER074 | 1053199.3	0	0	1	0	0	0	0	0	0	0
B. cereus VD078 | 1053227.3	0	0	1	0	0	0	0	0	0	0
B. cereus BDRD-ST196 | 526976.3	0	0	1	0	0	0	0	0	0	0
B. weihenstephanensis NBRC 101238 = DSM 11821 | 1220585.4	0	0	1	0	0	0	0	0	0	0
B. cereus AH621 | 526972.3	0	0	1	0	0	0	0	0	0	0
B. cereus VD048 | 1053226.3	0	0	1	0	0	0	0	0	0	0
B. cereus VD146 | 1053236.3	0	0	1	0	0	0	0	0	0	0
B. weihenstephanensis strain WSBC 10204 | 86662.17	0	0	1	0	0	0	0	0	0	0
B. cereus VDM034 | 1053248.3	0	0	1	0	0	0	0	0	0	0
B. cereus VDM062 | 1053250.3	0	0	1	0	0	0	0	0	0	0
B. weihenstephanensis strain JAS 83/3 | 86662.6	0	0	1	0	0	0	0	0	0	0
B. weihenstephanensis FSL H7-687 | 1227358.4	0	0	1	0	0	0	0	0	0	0
B. mycoides strain M2E_15 | 1405.18	0	0	1	0	0	0	0	0	0	0
B. mycoides strain WSBC 10969 | 1405.16	0	0	1	0	0	0	0	0	0	0
B. mycoides ATCC 6462 | 1405.10	0	0	1	0	0	0	0	0	0	0
B. cereus VDM022 | 1053247.3	0	0	1	0	0	0	0	0	0	0
B. cereus VDM019 | 1053246.3	0	0	1	0	0	0	0	0	0	0
B. cereus BAG5X1-1 | 1053189.3	0	0	0	0	0	0	0	0	1	0
B. cereus BAG6O-2 | 1053193.3	0	0	0	0	0	0	0	0	1	0
B. cereus VD107 | 1053229.3	0	0	0	0	0	0	1	0	0	0
B. cereus BAG6X1-2 | 1053195.3	0	0	0	0	0	0	0	0	1	0
B. cytotoxicus NVH 391-98 | 315749.8	0	0	0	0	0	0	0	0	0	0
B. cytotoxicus strain CVUAS 2833 | 580165.3	0	0	0	0	0	0	0	0	0	0
B. manliponensis strain JCM 15802 | 574376.4	1	0	0	0	0	0	0	0	0	0
B. bingmayongensis | 1150157.3	0	0	0	0	0	0	0	0	0	0
SUM	1	109	31	130	12	2	98	1	13	1

[a], Not detected or disrupted protein
[b], Accesion number from PATRIC database (https://www.patricbrc.org)

Table S4. In silico PCR results for the *B. cereus* group members with the primers specific for (i) the gene encoding L33-2 r-protein and (ii) strains from the r-cluster VIab. The in silico PCR was performed with Simulate_pcr v1.2 software[62]

		PCR specific for the gene encoding L33-2 r-protein [a] (Non-degenerate primers: 5'-GTTACTCGCTTTAATCGTGGAC-3' and 5'-ATCACCGCACTCTGTACAAG-3')							PCR specific for strains from the r-cluster VIab [a] (Non-degenerate primers: 5'-CAAAACCAAAAACCATTAGCAA-3' and 5'-GCAAGCAGCAAATGATGAGA-3')						
Strain	R-cluster	Amplicon length [bp]	Forward primer orientation	Sequence complementary to Forward primer	Foward primer mismatches	Reverse primer orientation	Sequence complementary to Reverse primer	Reverse primer mismatches	Amplicon length [bp]	Forward primer orientation	Sequence complementary to Forward primer	Foward primer mismatches	Reverse primer orientation	Sequence complementary to Reverse primer	Reverse primer mismatches
B. cereus VD136 | 1053234.3	Ia	106	F	GTTACTCGCTTTAATCGTGGAC	0	R	CTTGTACGGAGTGCGGTGAT	2	absent	-	-	-	-	-	-
B. cereus VDM006 | 1085379.3	Ia	106	F	GTTACTCGCTTTAATCGTGGAC	0	R	CTTGTACGGAGTGCGGTGAT	2	absent	-	-	-	-	-	-
B. cereus VDM021 | 1085386.3	Ia	106	F	GTTACTCGCTTTAATCGTGGAC	0	R	CTTGTACGGAGTGCGGTGAT	2	absent	-	-	-	-	-	-
B. mycoides strain 219298 | 1405.13	Ib	106	F	GTTACTCGCTTTAATCGTGGAC	0	R	CTTGTACGGAGTGCGGTGAT	2	absent	-	-	-	-	-	-
B. cereus Al Hakam | 1396.149	Ib	106	R	CTTGTACGGAGTGCGGTGAT	2	F	GTTACTCGCTTTAATCGTGGAC	0	absent	-	-	-	-	-	-
B. mycoides Flugge 10206 | 1405.9	Ib	106	R	CTTGTACGGAGTGCGGTGAT	2	F	GTTACTCGCTTTAATCGTGGAC	0	absent	-	-	-	-	-	-
B. mycoides Rock1-4 | 526998.3	Ib	106	R	CTTGTACGGAGTGCGGTGAT	2	F	GTTACTCGCTTTAATCGTGGAC	0	absent	-	-	-	-	-	-
B. mycoides Rock3-17 | 526999.3	Ib	106	F	GTTACTCGCTTTAATCGTGGAC	0	R	CTTGTACGGAGTGCGGTGAT	2	absent	-	-	-	-	-	-
B. pseudomycoides DSM 12442 | 527000.3	Ib	106	R	CTTGTACGGAGTGCGGTGAT	2	F	GTTACTCGCTTTAATCGTGGAC	0	absent	-	-	-	-	-	-
B. cereus Rock3-44 | 526986.3	Io	106	R	CTTGTACGGAGTGCGGTGAT	1	F	GTTACTCGCTTTAATCGTGGAC	1	absent	-	-	-	-	-	-
B. cereus BAG2O-3 | 1053178.3	IIa	106	R	CTTGTACGGAGTGCGGTGAT	1	F	GTTACTCGCTTTAATCGTGGAC	0	absent	-	-	-	-	-	-
B. cereus BAG3O-1 | 1085388.3	IIa	106	F	GTTACTCGCTTTAATCGTGGAC	0	R	CTTGTACGGAGTGCGGTGAT	1	absent	-	-	-	-	-	-
B. cereus B5-2 | 1085389.3	IIa	106	F	GTTACTCGCTTTAATCGTGGAC	0	R	CTTGTACGGAGTGCGGTGAT	1	absent	-	-	-	-	-	-
B. cereus BAG2X1-2 | 1053180.3	IIb	106	R	CTTGTACGGAGTGCGGTGAT	1	F	GTTACTCGCTTTAATCGTGGAC	0	absent	-	-	-	-	-	-
B. cereus BAG5X2-1 | 1053191.3	IIb	106	R	CTTGTACGGAGTGCGGTGAT	1	F	GTTACTCGCTTTAATCGTGGAC	0	absent	-	-	-	-	-	-
B. cereus BAG6X1-1 | 1053194.3	IIb	106	F	GTTACTCGCTTTAATCGTGGAC	0	R	CTTGTACGGAGTGCGGTGAT	1	absent	-	-	-	-	-	-
B. cereus strain 14294-3 (M6) | 1396.441	IIb	106	F	GTTACTCGCTTTAATCGTGGAC	0	R	CTTGTACGGAGTGCGGTGAT	1	absent	-	-	-	-	-	-
B. cereus strain MHI 226 | 1396.452	IIb	106	R	CTTGTACGGAGTGCGGTGAT	1	F	GTTACTCGCTTTAATCGTGGAC	0	absent	-	-	-	-	-	-
B. cereus strain RIMV BC 126 | 1396.449	IIb	106	R	CTTGTACGGAGTGCGGTGAT	1	F	GTTACTCGCTTTAATCGTGGAC	0	absent	-	-	-	-	-	-
B. cereus strain B4077 | 1396.428	IIb	106	R	CTTGTACGGAGTGCGGTGAT	0	F	GTTACTCGCTTTAATCGTGGAC	0	absent	-	-	-	-	-	-
B. cereus strain B4147 | 1396.433	IIb	106	F	GTTACTCGCTTTAATCGTGGAC	0	R	CTTGTACGGAGTGCGGTGAT	1	absent	-	-	-	-	-	-
B. cereus strain S612 | 1396.465	IIb	106	R	CTTGTACGGAGTGCGGTGAT	2	F	GTTACTCGCTTTAATCGTGGAC	0	absent	-	-	-	-	-	-
B. wiedmannii strain FSL W8-0169 | 1396.500	IIb	106	F	GTTACTCGCTTTAATCGTGGAC	0	R	CTTGTACGGAGTGCGGTGAT	1	absent	-	-	-	-	-	-
B. cereus MM3 | 526971.3	IIb	106	F	GTTACTCGCTTTAATCGTGGAC	0	R	CTTGTACGGAGTGCGGTGAT	0	absent	-	-	-	-	-	-
B. cereus strain FSL K6-0069 | 1396.516	IIc	106	R	CTTGTACGGAGTGCGGTGAT	0	F	GTTACTCGCTTTAATCGTGGAC	1	absent	-	-	-	-	-	-
B. cereus AH1271 | 526992.3	IIc	106	R	CTTGTACGGAGTGCGGTGAT	1	F	GTTACTCGCTTTAATCGTGGAC	0	absent	-	-	-	-	-	-
B. thuringiensis LM1212 | 1417985.4	IIIa	106	F	GTTACTCGCTTTAATCGTGGAC	0	R	CTTGTACGGAGTGCGGTGAT	1	absent	-	-	-	-	-	-
B. cereus FM1 | 1396.147	IIIa	106	R	CTTGTACGGAGTGCGGTGAT	2	F	GTTACTCGCTTTAATCGTGGAC	0	absent	-	-	-	-	-	-
B. cereus 03BB87 | 1396.146	IIIa	106	R	CTTGTACGGAGTGCGGTGAT	2	F	GTTACTCGCTTTAATCGTGGAC	0	absent	-	-	-	-	-	-
B. cereus strain BcFL2013 | 1396.419	IIIa	106	R	CTTGTACGGAGTGCGGTGAT	2	F	GTTACTCGCTTTAATCGTGGAC	0	absent	-	-	-	-	-	-
B. thuringiensis XL6 | 1428.243	IIIa	105	R	CTTGTACGGAGTGCGGTGAT	1	F	GTTACTCGCTTTAATCGTGGAC	0	absent	-	-	-	-	-	-
B. cereus strain SDA KA 96 | 1396.457	IIIa	106	F	GTTACTCGCTTTAATCGTGGAC	0	R	CTTGTACGGAGTGCGGTGAT	1	absent	-	-	-	-	-	-
B. cereus strain FSL K6-0067 | 1396.510	IIIa	106	R	CTTGTACGGAGTGCGGTGAT	2	F	GTTACTCGCTTTAATCGTGGAC	0	absent	-	-	-	-	-	-
B. cereus strain FSL M8-0117 | 1396.513	IIIa	106	R	CTTGTACGGAGTGCGGTGAT	1	F	GTTACTCGCTTTAATCGTGGAC	0	absent	-	-	-	-	-	-
B. thuringiensis strain KB1 | 1428.341	IIIa	106	F	GTTACTCGCTTTAATCGTGGAC	0	R	CTTGTACGGAGTGCGGTGAT	1	absent	-	-	-	-	-	-
B. cereus ATCC 4342 | 526977.3	IIIa	106	F	GTTACTCGCTTTAATCGTGGAC	0	R	CTTGTACGGAGTGCGGTGAT	1	absent	-	-	-	-	-	-
B. thuringiensis serovar tochigiensis BGSC 4Y1 | 527024.3	IIIa	105	F	GTTACTCGCTTTAATCGTGGAC	0	R	CTTGTACGGAGTGCGGTGAT	1	absent	-	-	-	-	-	-
B. cereus strain FT9 | 1396.420	IIIa	absent [b]	-	-	-	-	-	-	absent	-	-	-	-	-	-
B. anthracis strain 2000031021 | 1392.231	IIIb1	106	R	CTTGTACGGAGTGCGGTGAT	0	F	GTTACTCGCTTTAATCGTGGAC	0	absent	-	-	-	-	-	-
B. anthracis 2000031052 | 1392.63	IIIb1	106	R	CTTGTACGGAGTGCGGTGAT	0	F	GTTACTCGCTTTAATCGTGGAC	0	absent	-	-	-	-	-	-
B. anthracis 2002013094 | 1392.77	IIIb1	106	F	GTTACTCGCTTTAATCGTGGAC	0	R	CTTGTACGGAGTGCGGTGAT	0	absent	-	-	-	-	-	-
B. anthracis str. A1055 | 280355.4	IIIb1	106	F	GTTACTCGCTTTAATCGTGGAC	0	R	CTTGTACGGAGTGCGGTGAT	0	absent	-	-	-	-	-	-
B. cereus ISP3191 | 1053216.3	IIIb2	106	R	CTTGTACGGAGTGCGGTGAT	0	F	GTTACTCGCTTTAATCGTGGAC	0	absent	-	-	-	-	-	-
B. cereus F837/76 | 347495.3	IIIb2	106	F	GTTACTCGCTTTAATCGTGGAC	0	R	CTTGTACGGAGTGCGGTGAT	0	absent	-	-	-	-	-	-
B. cereus D17 | 1454382.3	IIIb2	106	R	CTTGTACGGAGTGCGGTGAT	0	F	GTTACTCGCTTTAATCGTGGAC	0	absent	-	-	-	-	-	-
B. thuringiensis HD1011 | 1428.249	IIIb2	106	F	GTTACTCGCTTTAATCGTGGAC	0	R	CTTGTACGGAGTGCGGTGAT	0	absent	-	-	-	-	-	-
B. cereus 3a | 1396.148	IIIb2	106	R	CTTGTACGGAGTGCGGTGAT	0	F	GTTACTCGCTTTAATCGTGGAC	0	absent	-	-	-	-	-	-
B. thuringiensis HD571 | 1428.250	IIIb2	106	F	GTTACTCGCTTTAATCGTGGAC	0	R	CTTGTACGGAGTGCGGTGAT	0	absent	-	-	-	-	-	-
B. cereus S2-8 | 1396.150	IIIb2	106	R	CTTGTACGGAGTGCGGTGAT	0	F	GTTACTCGCTTTAATCGTGGAC	0	absent	-	-	-	-	-	-
B. thuringiensis HD682 | 1428.251	IIIb2	106	R	CTTGTACGGAGTGCGGTGAT	0	F	GTTACTCGCTTTAATCGTGGAC	0	absent	-	-	-	-	-	-
B. cereus strain HWW 274-2 | 1396.442	IIIb2	106	R	CTTGTACGGAGTGCGGTGAT	0	F	GTTACTCGCTTTAATCGTGGAC	0	absent	-	-	-	-	-	-
B. cereus strain B4087 | 1396.432	IIIb2	106	R	CTTGTACGGAGTGCGGTGAT	0	F	GTTACTCGCTTTAATCGTGGAC	0	absent	-	-	-	-	-	-
B. anthracis strain RIT375 | 1392.242	IIIb2	106	R	CTTGTACGGAGTGCGGTGAT	0	F	GTTACTCGCTTTAATCGTGGAC	0	absent	-	-	-	-	-	-
B. cereus strain FSL W8-0275 | 1396.505	IIIb2	106	F	GTTACTCGCTTTAATCGTGGAC	0	R	CTTGTACGGAGTGCGGTGAT	0	absent	-	-	-	-	-	-
B. cereus strain FSL W8-0483 | 1396.520	IIIb2	106	R	CTTGTACGGAGTGCGGTGAT	0	F	GTTACTCGCTTTAATCGTGGAC	0	absent	-	-	-	-	-	-
B. cereus strain FSL W8-0932 | 1396.507	IIIb2	106	R	CTTGTACGGAGTGCGGTGAT	0	F	GTTACTCGCTTTAATCGTGGAC	0	absent	-	-	-	-	-	-
B. cereus strain PE8-121b | 1396.482	IIIb2	106	R	CTTGTACGGAGTGCGGTGAT	0	F	GTTACTCGCTTTAATCGTGGAC	0	absent	-	-	-	-	-	-
B. thuringiensis serovar konkukian str. 97-27 | 281309.8	IIIb2	106	F	GTTACTCGCTTTAATCGTGGAC	0	R	CTTGTACGGAGTGCGGTGAT	0	absent	-	-	-	-	-	-
B. cereus E33L | 288681.15	IIIb2	106	F	GTTACTCGCTTTAATCGTGGAC	0	R	CTTGTACGGAGTGCGGTGAT	0	absent	-	-	-	-	-	-
B. thuringiensis str. Al Hakam | 412694.7	IIIb2	106	F	GTTACTCGCTTTAATCGTGGAC	0	R	CTTGTACGGAGTGCGGTGAT	0	absent	-	-	-	-	-	-
B. cereus AH820 | 405535.8	IIIb2	106	F	GTTACTCGCTTTAATCGTGGAC	0	R	CTTGTACGGAGTGCGGTGAT	0	absent	-	-	-	-	-	-
B. cereus 03BB102 | 572264.4	IIIb2	106	F	GTTACTCGCTTTAATCGTGGAC	0	R	CTTGTACGGAGTGCGGTGAT	0	absent	-	-	-	-	-	-
B. cereus biovar anthracis str. CI | 637380.6	IIIb2	106	F	GTTACTCGCTTTAATCGTGGAC	0	R	CTTGTACGGAGTGCGGTGAT	0	absent	-	-	-	-	-	-
B. cereus SJ1 | 699184.4	IIIb2	105	R	CTTGTACGGAGTGCGGTGAT	0	F	GTTACTCGCTTTAATCGTGGAC	0	absent	-	-	-	-	-	-
B. cereus BGSC 6E1 | 526970.3	IIIb2	106	F	GTTACTCGCTTTAATCGTGGAC	0	R	CTTGTACGGAGTGCGGTGAT	0	absent	-	-	-	-	-	-
B. cereus 95/8201 | 526979.3	IIIb2	106	F	GTTACTCGCTTTAATCGTGGAC	0	R	CTTGTACGGAGTGCGGTGAT	0	absent	-	-	-	-	-	-
B. cereus Rock3-42 | 526985.3	IIIb2	106	R	CTTGTACGGAGTGCGGTGAT	0	F	GTTACTCGCTTTAATCGTGGAC	0	absent	-	-	-	-	-	-
B. thuringiensis serovar monterrey BGSC 4AJ1 | 527022.3	IIIb2	106	F	GTTACTCGCTTTAATCGTGGAC	0	R	CTTGTACGGAGTGCGGTGAT	0	absent	-	-	-	-	-	-
B. thuringiensis serovar andalousiensis BGSC 4AW1 | 527032.3	IIIb2	106	R	CTTGTACGGAGTGCGGTGAT	0	F	GTTACTCGCTTTAATCGTGGAC	0	absent	-	-	-	-	-	-
B. thuringiensis serovar pondicheriensis BGSC 4BA1 | 527029.3	IIIb2	106	F	GTTACTCGCTTTAATCGTGGAC	0	R	CTTGTACGGAGTGCGGTGAT	0	absent	-	-	-	-	-	-
B. thuringiensis serovar pulsiensis BGSC 4CC1 | 527028.3	IIIb2	106	R	CTTGTACGGAGTGCGGTGAT	0	F	GTTACTCGCTTTAATCGTGGAC	0	absent	-	-	-	-	-	-
B. cereus | 1396.463	IIIb2	disrupted gene	-	-	-	-	-	-	absent	-	-	-	-	-	-
B. anthracis str. Heroin Ba4599 | 1081943.3	IIIb3	106	F	GTTACTCGCTTTAATCGTGGAC	0	R	CTTGTACGGAGTGCGGTGAT	0	absent	-	-	-	-	-	-
B. anthracis str. UR-1 | 1211117.3	IIIb3	106	F	GTTACTCGCTTTAATCGTGGAC	0	R	CTTGTACGGAGTGCGGTGAT	0	absent	-	-	-	-	-	-
B. anthracis str. BF1 | 1213182.3	IIIb3	106	F	GTTACTCGCTTTAATCGTGGAC	0	R	CTTGTACGGAGTGCGGTGAT	0	absent	-	-	-	-	-	-
B. anthracis str. Carbosap | 1245029.3	IIIb3	106	F	GTTACTCGCTTTAATCGTGGAC	0	R	CTTGTACGGAGTGCGGTGAT	0	absent	-	-	-	-	-	-
B. anthracis str. 3154 | 1248431.3	IIIb3	106	F	GTTACTCGCTTTAATCGTGGAC	0	R	CTTGTACGGAGTGCGGTGAT	0	absent	-	-	-	-	-	-
B. anthracis str. 3166 | 1248436.3	IIIb3	106	F	GTTACTCGCTTTAATCGTGGAC	0	R	CTTGTACGGAGTGCGGTGAT	0	absent	-	-	-	-	-	-
B. anthracis V770-NP1-R | 1435357.3	IIIb3	106	F	GTTACTCGCTTTAATCGTGGAC	0	R	CTTGTACGGAGTGCGGTGAT	0	absent	-	-	-	-	-	-
B. anthracis CZC5 | 1439874.3	IIIb3	106	F	GTTACTCGCTTTAATCGTGGAC	0	R	CTTGTACGGAGTGCGGTGAT	0	absent	-	-	-	-	-	-
B. anthracis strain 44-NIAH | 1392.258	IIIb3	106	F	GTTACTCGCTTTAATCGTGGAC	0	R	CTTGTACGGAGTGCGGTGAT	0	absent	-	-	-	-	-	-
B. anthracis strain 52-40-NIAH | 1392.257	IIIb3	106	F	GTTACTCGCTTTAATCGTGGAC	0	R	CTTGTACGGAGTGCGGTGAT	0	absent	-	-	-	-	-	-
B. anthracis str. Sen2Col2 | 1297866.3	IIIb3	106	F	GTTACTCGCTTTAATCGTGGAC	0	R	CTTGTACGGAGTGCGGTGAT	0	absent	-	-	-	-	-	-
B. anthracis str. Sen3 | 1297867.3	IIIb3	106	F	GTTACTCGCTTTAATCGTGGAC	0	R	CTTGTACGGAGTGCGGTGAT	0	absent	-	-	-	-	-	-
B. anthracis str. Gmb1 | 1297868.3	IIIb3	106	F	GTTACTCGCTTTAATCGTGGAC	0	R	CTTGTACGGAGTGCGGTGAT	0	absent	-	-	-	-	-	-
B. anthracis 52-G | 1412844.4	IIIb3	106	F	GTTACTCGCTTTAATCGTGGAC	0	R	CTTGTACGGAGTGCGGTGAT	0	absent	-	-	-	-	-	-
B. anthracis 9080-G | 1412842.4	IIIb3	106	F	GTTACTCGCTTTAATCGTGGAC	0	R	CTTGTACGGAGTGCGGTGAT	0	absent	-	-	-	-	-	-
B. anthracis 8903-G | 1412843.4	IIIb3	106	F	GTTACTCGCTTTAATCGTGGAC	0	R	CTTGTACGGAGTGCGGTGAT	0	absent	-	-	-	-	-	-
B. anthracis strain Smith 1013 | 1392.239	IIIb3	106	F	GTTACTCGCTTTAATCGTGGAC	0	R	CTTGTACGGAGTGCGGTGAT	0	absent	-	-	-	-	-	-
B. anthracis str. A16 | 743835.4	IIIb3	106	F	GTTACTCGCTTTAATCGTGGAC	0	R	CTTGTACGGAGTGCGGTGAT	0	absent	-	-	-	-	-	-
B. anthracis str. A16R | 673518.9	IIIb3	106	F	GTTACTCGCTTTAATCGTGGAC	0	R	CTTGTACGGAGTGCGGTGAT	0	absent	-	-	-	-	-	-
B. anthracis str. H9401 | 768494.3	IIIb3	105	F	GTTACTCGCTTTAATCGTGGAC	0	R	CTTGTACGGAGTGCGGTGAT	0	absent	-	-	-	-	-	-
B. anthracis str. SVA11 | 1392837.3	IIIb3	106	F	GTTACTCGCTTTAATCGTGGAC	0	R	CTTGTACGGAGTGCGGTGAT	0	absent	-	-	-	-	-	-
B. anthracis strain BFV | 1392.233	IIIb3	106	F	GTTACTCGCTTTAATCGTGGAC	0	R	CTTGTACGGAGTGCGGTGAT	0	absent	-	-	-	-	-	-
B. anthracis strain delta Sterne | 1392.232	IIIb3	106	R	CTTGTACGGAGTGCGGTGAT	0	F	GTTACTCGCTTTAATCGTGGAC	0	absent	-	-	-	-	-	-
B. anthracis strain HYU01 | 1392.230	IIIb3	106	F	GTTACTCGCTTTAATCGTGGAC	0	R	CTTGTACGGAGTGCGGTGAT	0	absent	-	-	-	-	-	-
B. anthracis strain Cvac02 | 1392.236	IIIb3	107	F	GTTACTCGCTTTAATCGTGGAC	0	R	CTTGTACGGAGTGCGGTGAT	0	absent	-	-	-	-	-	-
B. anthracis strain Han | 1392.237	IIIb3	106	F	GTTACTCGCTTTAATCGTGGAC	0	R	CTTGTACGGAGTGCGGTGAT	0	absent	-	-	-	-	-	-
B. anthracis str. Turkey32 | 1452727.3	IIIb3	106	F	GTTACTCGCTTTAATCGTGGAC	0	R	CTTGTACGGAGTGCGGTGAT	0	absent	-	-	-	-	-	-
B. anthracis PAK-1 | 1392.85	IIIb3	106	R	CTTGTACGGAGTGCGGTGAT	0	F	GTTACTCGCTTTAATCGTGGAC	0	absent	-	-	-	-	-	-
B. anthracis Vollum 1B | 1392.86	IIIb3	106	F	GTTACTCGCTTTAATCGTGGAC	0	R	CTTGTACGGAGTGCGGTGAT	0	absent	-	-	-	-	-	-
B. anthracis K3 | 1392.87	IIIb3	106	R	CTTGTACGGAGTGCGGTGAT	0	F	GTTACTCGCTTTAATCGTGGAC	0	absent	-	-	-	-	-	-
B. anthracis Ohio ACB | 1392.88	IIIb3	106	F	GTTACTCGCTTTAATCGTGGAC	0	R	CTTGTACGGAGTGCGGTGAT	0	absent	-	-	-	-	-	-
B. anthracis SK-102 | 1392.89	IIIb3	106	R	CTTGTACGGAGTGCGGTGAT	0	F	GTTACTCGCTTTAATCGTGGAC	0	absent	-	-	-	-	-	-
B. anthracis Pasteur | 1392.90	IIIb3	106	R	CTTGTACGGAGTGCGGTGAT	0	F	GTTACTCGCTTTAATCGTGGAC	0	absent	-	-	-	-	-	-
B. anthracis BA1015 | 1392.91	IIIb3	106	R	CTTGTACGGAGTGCGGTGAT	0	F	GTTACTCGCTTTAATCGTGGAC	0	absent	-	-	-	-	-	-
B. anthracis str. V770-NP-1R | 1449979.3	IIIb3	106	R	CTTGTACGGAGTGCGGTGAT	0	F	GTTACTCGCTTTAATCGTGGAC	0	absent	-	-	-	-	-	-
B. anthracis RA3 | 1392.93	IIIb3	106	F	GTTACTCGCTTTAATCGTGGAC	0	R	CTTGTACGGAGTGCGGTGAT	0	absent	-	-	-	-	-	-
B. anthracis BA1035 | 1392.92	IIIb3	106	R	CTTGTACGGAGTGCGGTGAT	0	F	GTTACTCGCTTTAATCGTGGAC	0	absent	-	-	-	-	-	-
B. anthracis Ames_BA1004 | 1392.95	IIIb3	106	R	CTTGTACGGAGTGCGGTGAT	0	F	GTTACTCGCTTTAATCGTGGAC	0	absent	-	-	-	-	-	-
B. anthracis Canadian_bison | 1392.96	IIIb3	106	F	GTTACTCGCTTTAATCGTGGAC	0	R	CTTGTACGGAGTGCGGTGAT	0	absent	-	-	-	-	-	-
B. anthracis A0157 | 1392.82	IIIb3	106	F	GTTACTCGCTTTAATCGTGGAC	0	R	CTTGTACGGAGTGCGGTGAT	0	absent	-	-	-	-	-	-
B. anthracis Ames A0462 | 1392.83	IIIb3	106	F	GTTACTCGCTTTAATCGTGGAC	0	R	CTTGTACGGAGTGCGGTGAT	0	absent	-	-	-	-	-	-
B. anthracis Pollino | 1392.84	IIIb3	106	F	GTTACTCGCTTTAATCGTGGAC	0	R	CTTGTACGGAGTGCGGTGAT	0	absent	-	-	-	-	-	-
B. anthracis strain A1144 | 1392.238	IIIb3	106	F	GTTACTCGCTTTAATCGTGGAC	0	R	CTTGTACGGAGTGCGGTGAT	0	absent	-	-	-	-	-	-
B. anthracis strain Larissa | 1392.254	IIIb3	106	F	GTTACTCGCTTTAATCGTGGAC	0	R	CTTGTACGGAGTGCGGTGAT	0	absent	-	-	-	-	-	-
B. anthracis strain Stendal | 1392.269	IIIb3	106	F	GTTACTCGCTTTAATCGTGGAC	0	R	CTTGTACGGAGTGCGGTGAT	0	absent	-	-	-	-	-	-
B. anthracis str. 95014 | 1437442.3	IIIb3	106	F	GTTACTCGCTTTAATCGTGGAC	0	R	CTTGTACGGAGTGCGGTGAT	0	absent	-	-	-	-	-	-
B. anthracis strain ANSES_08-8_20 | 1392.227	IIIb3	106	F	GTTACTCGCTTTAATCGTGGAC	0	R	CTTGTACGGAGTGCGGTGAT	0	absent	-	-	-	-	-	-
B. anthracis strain ANSES_99-100 | 1392.228	IIIb3	106	F	GTTACTCGCTTTAATCGTGGAC	0	R	CTTGTACGGAGTGCGGTGAT	0	absent	-	-	-	-	-	-
B. anthracis strain ANSES_00-82 | 1392.229	IIIb3	106	F	GTTACTCGCTTTAATCGTGGAC	0	R	CTTGTACGGAGTGCGGTGAT	0	absent	-	-	-	-	-	-
B. anthracis 2000031031 | 1392.60	IIIb3	106	R	CTTGTACGGAGTGCGGTGAT	0	F	GTTACTCGCTTTAATCGTGGAC	0	absent	-	-	-	-	-	-
B. anthracis 2000031038 | 1392.61	IIIb3	105	R	CTTGTACGGAGTGCGGTGAT	0	F	GTTACTCGCTTTAATCGTGGAC	0	absent	-	-	-	-	-	-
B. anthracis 2000031039 | 1392.62	IIIb3	106	F	GTTACTCGCTTTAATCGTGGAC	0	R	CTTGTACGGAGTGCGGTGAT	0	absent	-	-	-	-	-	-
B. anthracis 2000031075 | 1392.64	IIIb3	106	R	CTTGTACGGAGTGCGGTGAT	0	F	GTTACTCGCTTTAATCGTGGAC	0	absent	-	-	-	-	-	-
B. anthracis 2000031709 | 1392.65	IIIb3	106	R	CTTGTACGGAGTGCGGTGAT	0	F	GTTACTCGCTTTAATCGTGGAC	0	absent	-	-	-	-	-	-
B. anthracis 2000031765 | 1392.66	IIIb3	106	R	CTTGTACGGAGTGCGGTGAT	0	F	GTTACTCGCTTTAATCGTGGAC	0	absent	-	-	-	-	-	-
B. anthracis 2000032819 | 1392.67	IIIb3	106	R	CTTGTACGGAGTGCGGTGAT	0	F	GTTACTCGCTTTAATCGTGGAC	0	absent	-	-	-	-	-	-
B. anthracis 2000032832 | 1392.68	IIIb3	106	R	CTTGTACGGAGTGCGGTGAT	0	F	GTTACTCGCTTTAATCGTGGAC	0	absent	-	-	-	-	-	-
B. anthracis 2000032879 | 1392.69	IIIb3	106	R	CTTGTACGGAGTGCGGTGAT	0	F	GTTACTCGCTTTAATCGTGGAC	0	absent	-	-	-	-	-	-
B. anthracis 2000032892 | 1392.70	IIIb3	106	F	GTTACTCGCTTTAATCGTGGAC	0	R	CTTGTACGGAGTGCGGTGAT	0	absent	-	-	-	-	-	-
B. anthracis 2000032951 | 1392.71	IIIb3	106	R	CTTGTACGGAGTGCGGTGAT	0	F	GTTACTCGCTTTAATCGTGGAC	0	absent	-	-	-	-	-	-
B. anthracis 2000032967 | 1392.72	IIIb3	106	F	GTTACTCGCTTTAATCGTGGAC	0	R	CTTGTACGGAGTGCGGTGAT	0	absent	-	-	-	-	-	-
B. anthracis 2000032968 | 1392.73	IIIb3	106	R	CTTGTACGGAGTGCGGTGAT	0	F	GTTACTCGCTTTAATCGTGGAC	0	absent	-	-	-	-	-	-
B. anthracis 2000032975 | 1392.74	IIIb3	106	F	GTTACTCGCTTTAATCGTGGAC	0	R	CTTGTACGGAGTGCGGTGAT	0	absent	-	-	-	-	-	-
B. anthracis 2000032979 | 1392.75	IIIb3	106	F	GTTACTCGCTTTAATCGTGGAC	0	R	CTTGTACGGAGTGCGGTGAT	0	absent	-	-	-	-	-	-
B. anthracis 2000032989 | 1392.76	IIIb3	106	F	GTTACTCGCTTTAATCGTGGAC	0	R	CTTGTACGGAGTGCGGTGAT	0	absent	-	-	-	-	-	-
B. anthracis 2000031008 | 1392.78	IIIb3	106	R	CTTGTACGGAGTGCGGTGAT	0	F	GTTACTCGCTTTAATCGTGGAC	0	absent	-	-	-	-	-	-
B. anthracis 2000031023 | 1392.79	IIIb3	106	R	CTTGTACGGAGTGCGGTGAT	0	F	GTTACTCGCTTTAATCGTGGAC	0	absent	-	-	-	-	-	-
B. anthracis 2000031027 | 1392.80	IIIb3	106	F	GTTACTCGCTTTAATCGTGGAC	0	R	CTTGTACGGAGTGCGGTGAT	0	absent	-	-	-	-	-	-
B. anthracis 2000031006 | 1392.81	IIIb3	106	F	GTTACTCGCTTTAATCGTGGAC	0	R	CTTGTACGGAGTGCGGTGAT	0	absent	-	-	-	-	-	-
B. anthracis strain Zimbabwe 89 | 1392.234	IIIb3	105	F	GTTACTCGCTTTAATCGTGGAC	0	R	CTTGTACGGAGTGCGGTGAT	0	absent	-	-	-	-	-	-
B. anthracis strain A.Br.003 | 1392.235	IIIb3	106	R	CTTGTACGGAGTGCGGTGAT	0	F	GTTACTCGCTTTAATCGTGGAC	0	absent	-	-	-	-	-	-
B. anthracis strain A1039 | 1392.240	IIIb3	106	R	CTTGTACGGAGTGCGGTGAT	0	F	GTTACTCGCTTTAATCGTGGAC	0	absent	-	-	-	-	-	-
B. anthracis strain A1075 | 1392.241	IIIb3	106	R	CTTGTACGGAGTGCGGTGAT	0	F	GTTACTCGCTTTAATCGTGGAC	0	absent	-	-	-	-	-	-
B. anthracis strain K1285 | 1392.243	IIIb3	106	R	CTTGTACGGAGTGCGGTGAT	0	F	GTTACTCGCTTTAATCGTGGAC	0	absent	-	-	-	-	-	-
B. anthracis strain K2129 | 1392.244	IIIb3	106	R	CTTGTACGGAGTGCGGTGAT	0	F	GTTACTCGCTTTAATCGTGGAC	0	absent	-	-	-	-	-	-
B. anthracis strain K2883 | 1392.245	IIIb3	106	R	CTTGTACGGAGTGCGGTGAT	0	F	GTTACTCGCTTTAATCGTGGAC	0	absent	-	-	-	-	-	-
B. anthracis strain K0021 | 1392.246	IIIb3	106	R	CTTGTACGGAGTGCGGTGAT	0	F	GTTACTCGCTTTAATCGTGGAC	0	absent	-	-	-	-	-	-
B. anthracis strain K4834 | 1392.247	IIIb3	106	F	GTTACTCGCTTTAATCGTGGAC	0	R	CTTGTACGGAGTGCGGTGAT	0	absent	-	-	-	-	-	-
B. anthracis strain PAK-1 | 1392.251	IIIb3	106	R	CTTGTACGGAGTGCGGTGAT	0	F	GTTACTCGCTTTAATCGTGGAC	0	absent	-	-	-	-	-	-
B. anthracis strain K3974 | 1392.248	IIIb3	106	F	GTTACTCGCTTTAATCGTGGAC	0	R	CTTGTACGGAGTGCGGTGAT	0	absent	-	-	-	-	-	-
B. anthracis strain K1129 | 1392.249	IIIb3	106	R	CTTGTACGGAGTGCGGTGAT	0	F	GTTACTCGCTTTAATCGTGGAC	0	absent	-	-	-	-	-	-
B. anthracis strain K8215 | 1392.250	IIIb3	106	F	GTTACTCGCTTTAATCGTGGAC	0	R	CTTGTACGGAGTGCGGTGAT	0	absent	-	-	-	-	-	-
B. anthracis strain A0021 | 1392.253	IIIb3	106	R	CTTGTACGGAGTGCGGTGAT	0	F	GTTACTCGCTTTAATCGTGGAC	0	absent	-	-	-	-	-	-
B. anthracis strain A0389 | 1392.252	IIIb3	106	R	CTTGTACGGAGTGCGGTGAT	0	F	GTTACTCGCTTTAATCGTGGAC	0	absent	-	-	-	-	-	-
B. anthracis str. Ames | 198094.11	IIIb3	106	F	GTTACTCGCTTTAATCGTGGAC	0	R	CTTGTACGGAGTGCGGTGAT	0	absent	-	-	-	-	-	-
B. anthracis str. Sterne | 260799.14	IIIb3	106	F	GTTACTCGCTTTAATCGTGGAC	0	R	CTTGTACGGAGTGCGGTGAT	0	absent	-	-	-	-	-	-
B. anthracis str. 'Ames Ancestor' | 261594.21	IIIb3	106	F	GTTACTCGCTTTAATCGTGGAC	0	R	CTTGTACGGAGTGCGGTGAT	0	absent	-	-	-	-	-	-
B. anthracis str. CDC 684 | 568206.3	IIIb3	106	F	GTTACTCGCTTTAATCGTGGAC	0	R	CTTGTACGGAGTGCGGTGAT	0	absent	-	-	-	-	-	-
B. anthracis str. A0248 | 592021.13	IIIb3	106	F	GTTACTCGCTTTAATCGTGGAC	0	R	CTTGTACGGAGTGCGGTGAT	0	absent	-	-	-	-	-	-
B. anthracis str. CNEVA-9066 | 280354.4	IIIb3	106	F	GTTACTCGCTTTAATCGTGGAC	0	R	CTTGTACGGAGTGCGGTGAT	0	absent	-	-	-	-	-	-
B. anthracis str. Vollum | 261591.4	IIIb3	106	F	GTTACTCGCTTTAATCGTGGAC	0	R	CTTGTACGGAGTGCGGTGAT	0	absent	-	-	-	-	-	-
B. anthracis str. Kruger B | 205919.4	IIIb3	106	F	GTTACTCGCTTTAATCGTGGAC	0	R	CTTGTACGGAGTGCGGTGAT	0	absent	-	-	-	-	-	-
B. anthracis str. Western North America USA6153 | 212045.4	IIIb3	106	F	GTTACTCGCTTTAATCGTGGAC	0	R	CTTGTACGGAGTGCGGTGAT	0	absent	-	-	-	-	-	-
B. cereus IS075 | 718220.3	IIIc	106	F	GTTACTCGCTTTAATCGTGGAC	0	R	CTTGTACGGAGTGCGGTGAT	1	absent	-	-	-	-	-	-
B. cereus AND1407 | 1053166.3	IIIc	106	R	CTTGTACGGAGTGCGGTGAT	1	F	GTTACTCGCTTTAATCGTGGAC	0	absent	-	-	-	-	-	-
B. cereus IS195 | 1053213.3	IIIc	106	F	GTTACTCGCTTTAATCGTGGAC	0	R	CTTGTACGGAGTGCGGTGAT	1	absent	-	-	-	-	-	-
B. cereus IS845/00 | 1053214.3	IIIc	106	F	GTTACTCGCTTTAATCGTGGAC	0	R	CTTGTACGGAGTGCGGTGAT	1	absent	-	-	-	-	-	-
B. cereus MSX-A12 | 1053221.3	IIIc	106	F	GTTACTCGCTTTAATCGTGGAC	0	R	CTTGTACGGAGTGCGGTGAT	1	absent	-	-	-	-	-	-
B. cereus MSX-D12 | 1053222.3	IIIc	106	R	CTTGTACGGAGTGCGGTGAT	0	F	GTTACTCGCTTTAATCGTGGAC	0	absent	-	-	-	-	-	-
B. cereus VD102 | 1053228.3	IIIc	106	R	CTTGTACGGAGTGCGGTGAT	0	F	GTTACTCGCTTTAATCGTGGAC	2	absent	-	-	-	-	-	-
B. cereus F | 1126681.3	IIIc	106	F	GTTACTCGCTTTAATCGTGGAC	0	R	CTTGTACGGAGTGCGGTGAT	1	absent	-	-	-	-	-	-
B. cereus LCT-BC244 | 1163722.3	IIIc	106	R	CTTGTACGGAGTGCGGTGAT	0	F	GTTACTCGCTTTAATCGTGGAC	0	absent	-	-	-	-	-	-
B. cereus LCT-BC25 | 1347791.3	IIIc	106	F	GTTACTCGCTTTAATCGTGGAC	0	R	CTTGTACGGAGTGCGGTGAT	0	absent	-	-	-	-	-	-
B. cereus LCT-BC235 | 1348059.3	IIIc	106	R	CTTGTACGGAGTGCGGTGAT	0	F	GTTACTCGCTTTAATCGTGGAC	0	absent	-	-	-	-	-	-
B. thuringiensis serovar finitimus YBT-020 | 930170.3	IIIc	106	F	GTTACTCGCTTTAATCGTGGAC	0	R	CTTGTACGGAGTGCGGTGAT	1	absent	-	-	-	-	-	-
B. cereus FRI-35 | 1217984.3	IIIc	106	R	CTTGTACGGAGTGCGGTGAT	1	F	GTTACTCGCTTTAATCGTGGAC	0	absent	-	-	-	-	-	-
B. cereus | 1396.464	IIIc	106	R	CTTGTACGGAGTGCGGTGAT	0	F	GTTACTCGCTTTAATCGTGGAC	0	absent	-	-	-	-	-	-
B. cereus strain 910_BCER | 1396.461	IIIc	106	R	CTTGTACGGAGTGCGGTGAT	1	F	GTTACTCGCTTTAATCGTGGAC	0	absent	-	-	-	-	-	-
B. cereus strain 896_BCER | 1396.460	IIIc	106	F	GTTACTCGCTTTAATCGTGGAC	0	R	CTTGTACGGAGTGCGGTGAT	1	absent	-	-	-	-	-	-
B. thuringiensis strain Lr3/2 | 1428.313	IIIc	106	R	CTTGTACGGAGTGCGGTGAT	1	F	GTTACTCGCTTTAATCGTGGAC	0	absent	-	-	-	-	-	-
B. thuringiensis strain Et10/1 | 1428.311	IIIc	106	R	CTTGTACGGAGTGCGGTGAT	1	F	GTTACTCGCTTTAATCGTGGAC	0	absent	-	-	-	-	-	-
B. cereus strain F4429-71 | 1396.443	IIIc	106	F	GTTACTCGCTTTAATCGTGGAC	0	R	CTTGTACGGAGTGCGGTGAT	0	absent	-	-	-	-	-	-
B. cereus strain F3162-04 | 1396.439	IIIc	106	F	GTTACTCGCTTTAATCGTGGAC	0	R	CTTGTACGGAGTGCGGTGAT	1	absent	-	-	-	-	-	-
B. cereus strain F1-15 | 1396.421	IIIc	106	R	CTTGTACGGAGTGCGGTGAT	1	F	GTTACTCGCTTTAATCGTGGAC	0	absent	-	-	-	-	-	-
B. cereus strain 13061 | 1396.422	IIIc	106	R	CTTGTACGGAGTGCGGTGAT	1	F	GTTACTCGCTTTAATCGTGGAC	0	absent	-	-	-	-	-	-
B. cereus strain MHI 86 | 1396.467	IIIc	106	F	GTTACTCGCTTTAATCGTGGAC	0	R	CTTGTACGGAGTGCGGTGAT	0	absent	-	-	-	-	-	-
B. cereus strain NVH 0075-95 | 1396.453	IIIc	106	F	GTTACTCGCTTTAATCGTGGAC	0	R	CTTGTACGGAGTGCGGTGAT	1	absent	-	-	-	-	-	-
B. cereus strain RIVM BC 90 | 1396.448	IIIc	106	F	GTTACTCGCTTTAATCGTGGAC	0	R	CTTGTACGGAGTGCGGTGAT	1	absent	-	-	-	-	-	-
B. cereus strain WSBC 10035 | 1396.455	IIIc	106	R	CTTGTACGGAGTGCGGTGAT	1	F	GTTACTCGCTTTAATCGTGGAC	0	absent	-	-	-	-	-	-
B. cereus strain 7/27/S | 1396.437	IIIc	106	R	CTTGTACGGAGTGCGGTGAT	0	F	GTTACTCGCTTTAATCGTGGAC	2	absent	-	-	-	-	-	-
B. cereus strain KS100Bc | 1396.427	IIIc	106	F	GTTACTCGCTTTAATCGTGGAC	0	R	CTTGTACGGAGTGCGGTGAT	1	absent	-	-	-	-	-	-
B. cereus strain B4078 | 1396.429	IIIc	106	R	CTTGTACGGAGTGCGGTGAT	1	F	GTTACTCGCTTTAATCGTGGAC	0	absent	-	-	-	-	-	-
B. cereus strain B4086 | 1396.430	IIIc	106	R	CTTGTACGGAGTGCGGTGAT	1	F	GTTACTCGCTTTAATCGTGGAC	0	absent	-	-	-	-	-	-
B. cereus strain B4153 | 1396.435	IIIc	106	F	GTTACTCGCTTTAATCGTGGAC	0	R	CTTGTACGGAGTGCGGTGAT	1	absent	-	-	-	-	-	-
B. cereus strain FSL W8-0003 | 1396.501	IIIc	106	F	GTTACTCGCTTTAATCGTGGAC	2	R	CTTGTACGGAGTGCGGTGAT	0	absent	-	-	-	-	-	-
B. cereus strain FSL W8-0050 | 1396.514	IIIc	106	R	CTTGTACGGAGTGCGGTGAT	1	F	GTTACTCGCTTTAATCGTGGAC	0	absent	-	-	-	-	-	-
B. cereus strain FSL W8-0523 | 1396.504	IIIc	106	R	CTTGTACGGAGTGCGGTGAT	1	F	GTTACTCGCTTTAATCGTGGAC	0	absent	-	-	-	-	-	-
B. cereus strain FSL W8-0520 | 1396.519	IIIc	106	F	GTTACTCGCTTTAATCGTGGAC	0	R	CTTGTACGGAGTGCGGTGAT	1	absent	-	-	-	-	-	-
B. cereus strain MB.17 | 1396.489	IIIc	106	F	GTTACTCGCTTTAATCGTGGAC	0	R	CTTGTACGGAGTGCGGTGAT	1	absent	-	-	-	-	-	-
B. cereus strain MB.15 | 1396.486	IIIc	106	F	GTTACTCGCTTTAATCGTGGAC	0	R	CTTGTACGGAGTGCGGTGAT	1	absent	-	-	-	-	-	-
B. cereus strain MB.1 | 1396.485	IIIc	106	R	CTTGTACGGAGTGCGGTGAT	1	F	GTTACTCGCTTTAATCGTGGAC	0	absent	-	-	-	-	-	-
B. cereus strain DSM 2302 | 1396.484	IIIc	106	F	GTTACTCGCTTTAATCGTGGAC	0	R	CTTGTACGGAGTGCGGTGAT	1	absent	-	-	-	-	-	-
B. cereus strain MB.21 | 1396.490	IIIc	106	R	CTTGTACGGAGTGCGGTGAT	1	F	GTTACTCGCTTTAATCGTGGAC	0	absent	-	-	-	-	-	-
B. cereus strain MB.8-1 | 1396.492	IIIc	106	R	CTTGTACGGAGTGCGGTGAT	1	F	GTTACTCGCTTTAATCGTGGAC	0	absent	-	-	-	-	-	-
B. cereus strain MB.8 | 1396.491	IIIc	106	R	CTTGTACGGAGTGCGGTGAT	1	F	GTTACTCGCTTTAATCGTGGAC	0	absent	-	-	-	-	-	-
B. cereus strain MB.16 | 1396.488	IIIc	106	F	GTTACTCGCTTTAATCGTGGAC	0	R	CTTGTACGGAGTGCGGTGAT	1	absent	-	-	-	-	-	-
B. cereus strain MB.18 | 1396.487	IIIc	106	F	GTTACTCGCTTTAATCGTGGAC	0	R	CTTGTACGGAGTGCGGTGAT	0	absent	-	-	-	-	-	-
B. cereus strain MB.20 | 1396.493	IIIc	106	F	GTTACTCGCTTTAATCGTGGAC	0	R	CTTGTACGGAGTGCGGTGAT	1	absent	-	-	-	-	-	-
B. cereus strain MB.22 | 1396.494	IIIc	106	R	CTTGTACGGAGTGCGGTGAT	1	F	GTTACTCGCTTTAATCGTGGAC	0	absent	-	-	-	-	-	-
B. cereus ATCC 10987 | 222523.6	IIIc	106	F	GTTACTCGCTTTAATCGTGGAC	0	R	CTTGTACGGAGTGCGGTGAT	1	absent	-	-	-	-	-	-
B. cereus AH187 | 405534.9	IIIc	106	F	GTTACTCGCTTTAATCGTGGAC	0	R	CTTGTACGGAGTGCGGTGAT	1	absent	-	-	-	-	-	-
B. cereus Q1 | 361100.6	IIIc	106	F	GTTACTCGCTTTAATCGTGGAC	0	R	CTTGTACGGAGTGCGGTGAT	1	absent	-	-	-	-	-	-
B. cereus NC7401 | 334406.4	IIIc	106	F	GTTACTCGCTTTAATCGTGGAC	0	R	CTTGTACGGAGTGCGGTGAT	1	absent	-	-	-	-	-	-
B. cereus m1293 | 526973.3	IIIc	106	R	CTTGTACGGAGTGCGGTGAT	0	F	GTTACTCGCTTTAATCGTGGAC	0	absent	-	-	-	-	-	-
B. cereus BDRD-ST26 | 526975.3	IIIc	106	R	CTTGTACGGAGTGCGGTGAT	1	F	GTTACTCGCTTTAATCGTGGAC	0	absent	-	-	-	-	-	-
B. thuringiensis strain CTC | 1428.335	IIo1	106	F	GTTACTCGCTTTAATCGTGGAC	0	R	CTTGTACGGAGTGCGGTGAT	1	absent	-	-	-	-	-	-
B. cereus R309803 | 526968.3	IIo1	106	R	CTTGTACGGAGTGCGGTGAT	1	F	GTTACTCGCTTTAATCGTGGAC	2	absent	-	-	-	-	-	-
B. cereus AH1272 | 526993.3	IIo2	absent	-	-	-	-	-	-	absent	-	-	-	-	-	-
B. cereus AH1273 | 526994.3	IIo2	absent	-	-	-	-	-	-	absent	-	-	-	-	-	-
B. cereus BAG1O-1 | 1053167.3	IIo2	absent	-	-	-	-	-	-	absent	-	-	-	-	-	-
B. cereus BAG1X1-3 | 1053172.3	IIo2	absent	-	-	-	-	-	-	absent	-	-	-	-	-	-
B. cereus BAG3X2-1 | 1053183.3	IIo2	absent	-	-	-	-	-	-	absent	-	-	-	-	-	-
B. cereus VDM053 | 1053249.3	IIo2	absent	-	-	-	-	-	-	absent	-	-	-	-	-	-
B. cereus strain F528-94 | 1396.440	IIo3	106	F	GTTACTCGCTTTAATCGTGGAC	0	R	CTTGTACGGAGTGCGGTGAT	1	absent	-	-	-	-	-	-
B. sp. 7_6_55CFAA_CT2 | 665957.3	IV-M	106	R	CTTGTACGGAGTGCGGTGAT	1	F	GTTACTCGCTTTAATCGTGGAC	0	absent	-	-	-	-	-	-
B. cereus BAG3O-2 | 1053182.3	IV-M	106	R	CTTGTACGGAGTGCGGTGAT	1	F	GTTACTCGCTTTAATCGTGGAC	0	absent	-	-	-	-	-	-
B. cereus BAG3X2-2 | 1053184.3	IV-M	106	F	GTTACTCGCTTTAATCGTGGAC	0	R	CTTGTACGGAGTGCGGTGAT	0	absent	-	-	-	-	-	-
B. cereus BAG4O-1 | 1053185.3	IV-M	106	F	GTTACTCGCTTTAATCGTGGAC	0	R	CTTGTACGGAGTGCGGTGAT	1	absent	-	-	-	-	-	-
B. cereus BAG4X12-1 | 1053186.3	IV-M	106	F	GTTACTCGCTTTAATCGTGGAC	0	R	CTTGTACGGAGTGCGGTGAT	0	absent	-	-	-	-	-	-
B. cereus BAG5X12-1 | 1053190.3	IV-M	106	R	CTTGTACGGAGTGCGGTGAT	0	F	GTTACTCGCTTTAATCGTGGAC	0	absent	-	-	-	-	-	-
B. cereus BMG1.7 | 1053196.3	IV-M	106	R	CTTGTACGGAGTGCGGTGAT	0	F	GTTACTCGCTTTAATCGTGGAC	0	absent	-	-	-	-	-	-
B. cereus HD73 | 1053200.3	IV-M	106	R	CTTGTACGGAGTGCGGTGAT	0	F	GTTACTCGCTTTAATCGTGGAC	0	absent	-	-	-	-	-	-
B. cereus HuB13-1 | 1053208.3	IV-M	106	F	GTTACTCGCTTTAATCGTGGAC	0	R	CTTGTACGGAGTGCGGTGAT	0	absent	-	-	-	-	-	-
B. cereus ISP2954 | 1053215.3	IV-M	106	F	GTTACTCGCTTTAATCGTGGAC	0	R	CTTGTACGGAGTGCGGTGAT	0	absent	-	-	-	-	-	-
B. cereus VD014 | 1053223.3	IV-M	106	F	GTTACTCGCTTTAATCGTGGAC	0	R	CTTGTACGGAGTGCGGTGAT	0	absent	-	-	-	-	-	-
B. cereus VD045 | 1053225.3	IV-M	106	R	CTTGTACGGAGTGCGGTGAT	1	F	GTTACTCGCTTTAATCGTGGAC	0	absent	-	-	-	-	-	-
B. cereus VD133 | 1053233.3	IV-M	105	F	GTTACTCGCTTTAATCGTGGAC	0	R	CTTGTACGGAGTGCGGTGAT	0	absent	-	-	-	-	-	-
B. cereus VD140 | 1053235.3	IV-M	106	F	GTTACTCGCTTTAATCGTGGAC	1	R	CTTGTACGGAGTGCGGTGAT	1	absent	-	-	-	-	-	-
B. cereus VD156 | 1053239.3	IV-M	106	R	CTTGTACGGAGTGCGGTGAT	0	F	GTTACTCGCTTTAATCGTGGAC	0	absent	-	-	-	-	-	-
B. cereus VD166 | 1053240.3	IV-M	106	R	CTTGTACGGAGTGCGGTGAT	1	F	GTTACTCGCTTTAATCGTGGAC	0	absent	-	-	-	-	-	-
B. cereus VD169 | 1053241.3	IV-M	106	F	GTTACTCGCTTTAATCGTGGAC	0	R	CTTGTACGGAGTGCGGTGAT	1	absent	-	-	-	-	-	-
B. cereus VD184 | 1053242.3	IV-M	106	F	GTTACTCGCTTTAATCGTGGAC	0	R	CTTGTACGGAGTGCGGTGAT	1	absent	-	-	-	-	-	-
B. cereus VD196 | 1053243.3	IV-M	106	R	CTTGTACGGAGTGCGGTGAT	1	F	GTTACTCGCTTTAATCGTGGAC	0	absent	-	-	-	-	-	-
B. cereus VD200 | 1053244.3	IV-M	106	F	GTTACTCGCTTTAATCGTGGAC	0	R	CTTGTACGGAGTGCGGTGAT	1	absent	-	-	-	-	-	-
B. thuringiensis serovar aizawai str. Leapi01 | 1232203.4	IV-M	106	F	GTTACTCGCTTTAATCGTGGAC	0	R	CTTGTACGGAGTGCGGTGAT	0	absent	-	-	-	-	-	-
B. thuringiensis serovar aizawai str. Hu4-2 | 1238885.4	IV-M	106	R	CTTGTACGGAGTGCGGTGAT	0	F	GTTACTCGCTTTAATCGTGGAC	0	absent	-	-	-	-	-	-
B. thuringiensis serovar tolworthi | 1442.3	IV-M	106	R	CTTGTACGGAGTGCGGTGAT	1	F	GTTACTCGCTTTAATCGTGGAC	0	absent	-	-	-	-	-	-
B. weihenstephanensis FSL R5-860 | 1227359.4	IV-M	106	R	CTTGTACGGAGTGCGGTGAT	2	F	GTTACTCGCTTTAATCGTGGAC	0	absent	-	-	-	-	-	-
B. thuringiensis DB27 | 1431339.3	IV-M	106	F	GTTACTCGCTTTAATCGTGGAC	1	R	CTTGTACGGAGTGCGGTGAT	0	absent	-	-	-	-	-	-
B. thuringiensis serovar kurstaki str. HD73 | 1279365.3	IV-M	106	F	GTTACTCGCTTTAATCGTGGAC	0	R	CTTGTACGGAGTGCGGTGAT	0	absent	-	-	-	-	-	-
B. bombysepticus str. Wang | 1330043.5	IV-M	106	F	GTTACTCGCTTTAATCGTGGAC	1	R	CTTGTACGGAGTGCGGTGAT	0	absent	-	-	-	-	-	-
B. cereus strain FORC_005 | 1396.426	IV-M	106	F	GTTACTCGCTTTAATCGTGGAC	0	R	CTTGTACGGAGTGCGGTGAT	2	absent	-	-	-	-	-	-
B. thuringiensis serovar kurstaki HD 1 | 29339.37	IV-M	106	R	CTTGTACGGAGTGCGGTGAT	0	F	GTTACTCGCTTTAATCGTGGAC	0	absent	-	-	-	-	-	-
B. thuringiensis serovar galleriae HD-29 | 29338.3	IV-M	106	F	GTTACTCGCTTTAATCGTGGAC	0	R	CTTGTACGGAGTGCGGTGAT	0	absent	-	-	-	-	-	-
B. thuringiensis serovar indiana strain HD521 | 180850.5	IV-M	106	F	GTTACTCGCTTTAATCGTGGAC	0	R	CTTGTACGGAGTGCGGTGAT	1	absent	-	-	-	-	-	-
B. cereus strain FORC_013 | 1396.474	IV-M	106	R	CTTGTACGGAGTGCGGTGAT	1	F	GTTACTCGCTTTAATCGTGGAC	0	absent	-	-	-	-	-	-
B. thuringiensis strain YC-10 | 1428.325	IV-M	106	R	CTTGTACGGAGTGCGGTGAT	0	F	GTTACTCGCTTTAATCGTGGAC	0	absent	-	-	-	-	-	-
B. thuringiensis strain YWC2-8 | 1428.324	IV-M	106	R	CTTGTACGGAGTGCGGTGAT	0	F	GTTACTCGCTTTAATCGTGGAC	0	absent	-	-	-	-	-	-
B. cereus strain A1 | 1396.418	IV-M	106	F	GTTACTCGCTTTAATCGTGGAC	0	R	CTTGTACGGAGTGCGGTGAT	1	absent	-	-	-	-	-	-
B. cereus tsu1 | 1396.141	IV-M	106	F	GTTACTCGCTTTAATCGTGGAC	0	R	CTTGTACGGAGTGCGGTGAT	0	absent	-	-	-	-	-	-
B. thuringiensis strain 823_BTHU | 1428.316	IV-M	106	F	GTTACTCGCTTTAATCGTGGAC	0	R	CTTGTACGGAGTGCGGTGAT	2	absent	-	-	-	-	-	-
B. cereus VD154 | 1053238.3	IV-M	106	F	GTTACTCGCTTTAATCGTGGAC	0	R	CTTGTACGGAGTGCGGCAAT	2	absent	-	-	-	-	-	-
B. cereus strain 822_BCER | 1396.466	IV-M	106	R	CTTGTACGGAGTGCGGTGAT	2	F	GTTACTCGCTTTAATCGTGGAC	0	absent	-	-	-	-	-	-
B. cereus strain 820_BCER | 1396.459	IV-M	106	F	GTTACTCGCTTTAATCGTGGAC	0	R	CTTGTACGGAGTGCGGTGAT	2	absent	-	-	-	-	-	-
B. thuringiensis serovar mexicanensis strain 27 | 180868.3	IV-M	106	F	GTTACTCGCTTTAATCGTGGAC	0	R	CTTGTACGGAGTGCGGTGAT	0	absent	-	-	-	-	-	-
B. thuringiensis AK47 | 1428.247	IV-M	106	R	CTTGTACGGAGTGCGGTGAT	0	F	GTTACTCGCTTTAATCGTGGAC	0	absent	-	-	-	-	-	-
B. cereus strain MIT0214 | 1396.423	IV-M	106	F	GTTACTCGCTTTAATCGTGGAC	0	R	CTTGTACGGAGTGCGGTGAT	1	absent	-	-	-	-	-	-
B. thuringiensis Sbt003 | 1235825.4	IV-M	106	F	GTTACTCGCTTTAATCGTGGAC	0	R	CTTGTACGGAGTGCGGCAAT	2	absent	-	-	-	-	-	-
B. thuringiensis serovar pakistani str. T13001 | 527027.3	IV-M	106	F	GTTACTCGCTTTAATCGTGGAC	0	R	CTTGTACGGAGTGCGGCAAT	2	absent	-	-	-	-	-	-
B. cereus strain Lr 4/2 | 1396.425	IV-M	106	R	CTTGTACGGAGTGCGGTGAT	1	F	GTTACTCGCTTTAATCGTGGAC	1	absent	-	-	-	-	-	-
B. cereus strain #17 | 1396.438	IV-M	106	F	GTTACTCGCTTTAATCGTGGAC	0	R	CTTGTACGGAGTGCGGTGAT	1	absent	-	-	-	-	-	-
B. cereus strain F3175-03 | 1396.444	IV-M	106	R	CTTGTACGGAGTGCGGTGAT	0	F	GTTACTCGCTTTAATCGTGGAC	1	absent	-	-	-	-	-	-
B. cereus strain F4430-73 | 1396.446	IV-M	106	F	GTTACTCGCTTTAATCGTGGAC	0	R	CTTGTACGGAGTGCGGTGAT	1	absent	-	-	-	-	-	-
B. thuringiensis strain NBIN-866 | 1428.310	IV-M	106	F	GTTACTCGCTTTAATCGTGGAC	0	R	CTTGTACGGAGTGCGGTGAT	0	absent	-	-	-	-	-	-
B. cereus strain INRA A3 | 1396.445	IV-M	106	R	CTTGTACGGAGTGCGGTGAT	2	F	GTTACTCGCTTTAATCGTGGAC	0	absent	-	-	-	-	-	-
B. cereus strain INRA C3 | 1396.447	IV-M	106	R	CTTGTACGGAGTGCGGTGAT	1	F	GTTACTCGCTTTAATCGTGGAC	0	absent	-	-	-	-	-	-
B. thuringiensis T01-328 | 1324966.3	IV-M	absent [c]	-	-	-	-	-	-	absent	-	-	-	-	-	-
B. cereus strain RIVM BC 934 | 1396.456	IV-M	106	R	CTTGTACGGAGTGCGGTGAT	0	F	GTTACTCGCTTTAATCGTGGAC	0	absent	-	-	-	-	-	-
B. cereus strain 6/27/S | 1396.458	IV-M	106	R	CTTGTACGGAGTGCGGTGAT	0	F	GTTACTCGCTTTAATCGTGGAC	0	absent	-	-	-	-	-	-
B. cereus strain B4080 | 1396.431	IV-M	106	F	GTTACTCGCTTTAATCGTGGAC	0	R	CTTGTACGGAGTGCGGTGAT	0	absent	-	-	-	-	-	-
B. cereus strain B4158 | 1396.434	IV-M	106	F	GTTACTCGCTTTAATCGTGGAC	0	R	CTTGTACGGAGTGCGGTGAT	0	absent	-	-	-	-	-	-
B. thuringiensis strain BMB3201 | 1428.320	IV-M	106	R	CTTGTACGGAGTGCGGTGAT	0	F	GTTACTCGCTTTAATCGTGGAC	0	absent	-	-	-	-	-	-
B. cereus strain LK9 | 1396.436	IV-M	106	F	GTTACTCGCTTTAATCGTGGAC	0	R	CTTGTACGGAGTGCGGTGAT	0	absent	-	-	-	-	-	-
B. thuringiensis strain BR37 | 1428.336	IV-M	106	F	GTTACTCGCTTTAATCGTGGAC	0	R	CTTGTACGGAGTGCGGTGAT	0	absent	-	-	-	-	-	-
B. cereus strain FSL K6-0073 | 1396.511	IV-M	106	F	GTTACTCGCTTTAATCGTGGAC	1	R	CTTGTACGGAGTGCGGTGAT	1	absent	-	-	-	-	-	-
B. cereus strain FSL W8-0268 | 1396.502	IV-M	106	F	GTTACTCGCTTTAATCGTGGAC	0	R	CTTGTACGGAGTGCGGTGAT	2	absent	-	-	-	-	-	-
B. cereus strain FSL W8-0640 | 1396.509	IV-M	106	F	GTTACTCGCTTTAATCGTGGAC	0	R	CTTGTACGGAGTGCGGTGAT	0	absent	-	-	-	-	-	-
B. cereus strain FSL W8-0824 | 1396.508	IV-M	106	F	GTTACTCGCTTTAATCGTGGAC	0	R	CTTGTACGGAGTGCGGTGAT	1	absent	-	-	-	-	-	-
B. cereus strain FSL W8-0767 | 1396.506	IV-M	106	R	CTTGTACGGAGTGCGGTGAT	0	F	GTTACTCGCTTTAATCGTGGAC	0	absent	-	-	-	-	-	-
B. cereus strain DSM 626 | 1396.495	IV-M	106	R	CTTGTACGGAGTGCGGTGAT	2	F	GTTACTCGCTTTAATCGTGGAC	0	absent	-	-	-	-	-	-
B. cereus strain 905 | 1396.498	IV-M	106	F	GTTACTCGCTTTAATCGTGGAC	0	R	CTTGTACGGAGTGCGGTGAT	1	absent	-	-	-	-	-	-
B. cereus ATCC 14579 | 226900.8	IV-M	106	F	GTTACTCGCTTTAATCGTGGAC	0	R	CTTGTACGGAGTGCGGTGAT	2	absent	-	-	-	-	-	-
B. cereus B4264 | 405532.5	IV-M	106	F	GTTACTCGCTTTAATCGTGGAC	0	R	CTTGTACGGAGTGCGGTGAT	2	absent	-	-	-	-	-	-
B. thuringiensis BMB171 | 714359.3	IV-M	106	F	GTTACTCGCTTTAATCGTGGAC	0	R	CTTGTACGGAGTGCGGTGAT	1	absent	-	-	-	-	-	-
B. cereus AH1134 | 405533.4	IV-M	106	F	GTTACTCGCTTTAATCGTGGAC	0	R	CTTGTACGGAGTGCGGTGAT	0	absent	-	-	-	-	-	-
B. cereus ATCC 10876 | 526980.3	IV-M	106	R	CTTGTACGGAGTGCGGTGAT	1	F	GTTACTCGCTTTAATCGTGGAC	1	absent	-	-	-	-	-	-
B. cereus 172560W | 526967.3	IV-M	106	R	CTTGTACGGAGTGCGGTGAT	0	F	GTTACTCGCTTTAATCGTGGAC	1	absent	-	-	-	-	-	-
B. cereus m1550 | 526969.3	IV-M	106	R	CTTGTACGGAGTGCGGTGAT	2	F	GTTACTCGCTTTAATCGTGGAC	0	absent	-	-	-	-	-	-
B. cereus BDRD-ST24 | 526974.3	IV-M	106	R	CTTGTACGGAGTGCGGTGAT	1	F	GTTACTCGCTTTAATCGTGGAC	0	absent	-	-	-	-	-	-
B. cereus BDRD-Cer4 | 526978.3	IV-M	106	R	CTTGTACGGAGTGCGGTGAT	2	F	GTTACTCGCTTTAATCGTGGAC	0	absent	-	-	-	-	-	-
B. cereus Rock1-15 | 526982.3	IV-M	106	F	GTTACTCGCTTTAATCGTGGAC	0	R	CTTGTACGGAGTGCGGTGAT	1	absent	-	-	-	-	-	-
B. cereus Rock4-2 | 526987.3	IV-M	106	R	CTTGTACGGAGTGCGGTGAT	0	F	GTTACTCGCTTTAATCGTGGAC	0	absent	-	-	-	-	-	-
B. cereus F65185 | 526989.3	IV-M	106	R	CTTGTACGGAGTGCGGTGAT	0	F	GTTACTCGCTTTAATCGTGGAC	0	absent	-	-	-	-	-	-
B. cereus AH676 | 526991.3	IV-M	106	R	CTTGTACGGAGTGCGGTGAT	1	F	GTTACTCGCTTTAATCGTGGAC	0	absent	-	-	-	-	-	-
B. thuringiensis serovar kurstaki str. T03a001 | 527023.3	IV-M	106	F	GTTACTCGCTTTAATCGTGGAC	0	R	CTTGTACGGAGTGCGGTGAT	0	absent	-	-	-	-	-	-
B. thuringiensis serovar huazhongensis BGSC 4BD1 | 527030.3	IV-M	106	R	CTTGTACGGAGTGCGGTGAT	0	F	GTTACTCGCTTTAATCGTGGAC	0	absent	-	-	-	-	-	-
B. cereus BAG1O-3 | 1053169.3	IV-Ia1	106	R	CTTGTACGGAGTGCGGTGAT	0	F	GTTACTCGCTTTAATCGTGGAC	0	absent	-	-	-	-	-	-
B. cereus BAG1X1-2 | 1053171.3	IV-Ia1	106	F	GTTACTCGCTTTAATCGTGGAC	0	R	CTTGTACGGAGTGCGGTGAT	0	absent	-	-	-	-	-	-
B. cereus HuB1-1 | 1053207.3	IV-Ia1	106	R	CTTGTACGGAGTGCGGTGAT	0	F	GTTACTCGCTTTAATCGTGGAC	0	absent	-	-	-	-	-	-
B. thuringiensis serovar tolworthi NA205-2 | 1306023.3	IV-Ia1	106	F	GTTACTCGCTTTAATCGTGGAC	0	R	CTTGTACGGAGTGCGGTGAT	0	absent	-	-	-	-	-	-
B. thuringiensis serovar chinensis CT-43 | 541229.3	IV-Ia1	106	F	GTTACTCGCTTTAATCGTGGAC	0	R	CTTGTACGGAGTGCGGTGAT	0	absent	-	-	-	-	-	-
B. thuringiensis serovar thuringiensis str. IS5056 | 1286404.3	IV-Ia1	106	F	GTTACTCGCTTTAATCGTGGAC	0	R	CTTGTACGGAGTGCGGTGAT	0	absent	-	-	-	-	-	-
B. thuringiensis YBT-1518 | 529122.4	IV-Ia1	106	F	GTTACTCGCTTTAATCGTGGAC	0	R	CTTGTACGGAGTGCGGTGAT	0	absent	-	-	-	-	-	-
B. cereus strain NJ-W | 1396.462	IV-Ia1	106	R	CTTGTACGGAGTGCGGTGAT	1	F	GTTACTCGCTTTAATCGTGGAC	0	absent	-	-	-	-	-	-
B. cereus strain FSL K6-0043 | 1396.499	IV-Ia1	106	F	GTTACTCGCTTTAATCGTGGAC	0	R	CTTGTACGGAGTGCGGTGAT	0	absent	-	-	-	-	-	-
B. thuringiensis Bt407 | 527021.3	IV-Ia1	106	R	CTTGTACGGAGTGCGGTGAT	0	F	GTTACTCGCTTTAATCGTGGAC	0	absent	-	-	-	-	-	-
B. thuringiensis serovar sotto str. T04001 | 527026.3	IV-Ia1	106	R	CTTGTACGGAGTGCGGTGAT	0	F	GTTACTCGCTTTAATCGTGGAC	0	absent	-	-	-	-	-	-
B. thuringiensis serovar berliner ATCC 10792 | 527031.3	IV-Ia1	106	F	GTTACTCGCTTTAATCGTGGAC	0	R	CTTGTACGGAGTGCGGTGAT	0	absent	-	-	-	-	-	-
B. cereus BAG1X1-1 | 1053170.3	IV-Ia2	106	R	CTTGTACGGAGTGCGGTGAT	0	F	GTTACTCGCTTTAATCGTGGAC	0	absent	-	-	-	-	-	-
B. cereus BAG1X2-1 | 1053173.3	IV-Ia2	106	F	GTTACTCGCTTTAATCGTGGAC	0	R	CTTGTACGGAGTGCGGTGAT	0	absent	-	-	-	-	-	-
B. cereus BAG1X2-2 | 1053174.3	IV-Ia2	106	R	CTTGTACGGAGTGCGGTGAT	0	F	GTTACTCGCTTTAATCGTGGAC	0	absent	-	-	-	-	-	-
B. cereus BAG1X2-3 | 1053175.3	IV-Ia2	106	F	GTTACTCGCTTTAATCGTGGAC	0	R	CTTGTACGGAGTGCGGTGAT	0	absent	-	-	-	-	-	-
B. cereus BAG2O-1 | 1053176.3	IV-Ia2	106	F	GTTACTCGCTTTAATCGTGGAC	0	R	CTTGTACGGAGTGCGGTGAT	0	absent	-	-	-	-	-	-
B. thuringiensis JM-Mgvxx-63 | 1423139.4	IV-Ia2	106	F	GTTACTCGCTTTAATCGTGGAC	0	R	CTTGTACGGAGTGCGGTGAT	0	absent	-	-	-	-	-	-
B. thuringiensis strain HS18-1 | 1428.317	IV-Ia2	106	F	GTTACTCGCTTTAATCGTGGAC	0	R	CTTGTACGGAGTGCGGTGAT	0	absent	-	-	-	-	-	-
B. cereus strain RIVM BC 964 | 1396.454	IV-Ia2	106	R	CTTGTACGGAGTGCGGTGAT	0	F	GTTACTCGCTTTAATCGTGGAC	0	absent	-	-	-	-	-	-
B. cereus str. Schrouff | 718221.3	IV-Ia3	106	R	CTTGTACGGAGTGCGGTGAT	0	F	GTTACTCGCTTTAATCGTGGAC	0	absent	-	-	-	-	-	-
B. cereus TIAC219 | 718222.3	IV-Ia3	106	F	GTTACTCGCTTTAATCGTGGAC	0	R	CTTGTACGGAGTGCGGTGAT	0	absent	-	-	-	-	-	-
B. cereus VD022 | 718223.3	IV-Ia3	106	R	CTTGTACGGAGTGCGGTGAT	0	F	GTTACTCGCTTTAATCGTGGAC	0	absent	-	-	-	-	-	-
B. cereus HuB4-4 | 1053211.3	IV-Ia3	106	F	GTTACTCGCTTTAATCGTGGAC	0	R	CTTGTACGGAGTGCGGTGAT	0	absent	-	-	-	-	-	-
B. cereus K-5975c | 1053217.3	IV-Ia3	106	R	CTTGTACGGAGTGCGGTGAT	0	F	GTTACTCGCTTTAATCGTGGAC	0	absent	-	-	-	-	-	-
B. cereus MSX-A1 | 1053220.3	IV-Ia3	106	R	CTTGTACGGAGTGCGGTGAT	0	F	GTTACTCGCTTTAATCGTGGAC	0	absent	-	-	-	-	-	-
B. thuringiensis DAR 81934 | 1257079.3	IV-Ia3	106	F	GTTACTCGCTTTAATCGTGGAC	0	R	CTTGTACGGAGTGCGGTGAT	0	absent	-	-	-	-	-	-
B. thuringiensis HD-771 | 1218175.3	IV-Ia3	106	R	CTTGTACGGAGTGCGGTGAT	0	F	GTTACTCGCTTTAATCGTGGAC	0	absent	-	-	-	-	-	-
B. thuringiensis HD-789 | 1217737.3	IV-Ia3	106	F	GTTACTCGCTTTAATCGTGGAC	0	R	CTTGTACGGAGTGCGGTGAT	0	absent	-	-	-	-	-	-
B. thuringiensis HD1002 | 1452729.3	IV-Ia3	106	R	CTTGTACGGAGTGCGGTGAT	0	F	GTTACTCGCTTTAATCGTGGAC	0	absent	-	-	-	-	-	-
B. thuringiensis serovar morrisoni strain BGSC 4AA1 | 1441.4	IV-Ia3	106	F	GTTACTCGCTTTAATCGTGGAC	0	R	CTTGTACGGAGTGCGGTGAT	0	absent	-	-	-	-	-	-
B. thuringiensis serovar israelensis strain 4Q7 | 1430.6	IV-Ia3	106	F	GTTACTCGCTTTAATCGTGGAC	0	R	CTTGTACGGAGTGCGGTGAT	0	absent	-	-	-	-	-	-
B. thuringiensis serovar morrisoni HD 600 | 1441.3	IV-Ia3	106	F	GTTACTCGCTTTAATCGTGGAC	0	R	CTTGTACGGAGTGCGGTGAT	0	absent	-	-	-	-	-	-
B. thuringiensis strain Lr7/2 | 1428.312	IV-Ia3	106	F	GTTACTCGCTTTAATCGTGGAC	0	R	CTTGTACGGAGTGCGGTGAT	0	absent	-	-	-	-	-	-
B. thuringiensis strain 147 | 1428.321	IV-Ia3	106	F	GTTACTCGCTTTAATCGTGGAC	0	R	CTTGTACGGAGTGCGGTGAT	0	absent	-	-	-	-	-	-
B. thuringiensis strain BR58 | 1428.322	IV-Ia3	106	R	CTTGTACGGAGTGCGGTGAT	0	F	GTTACTCGCTTTAATCGTGGAC	0	absent	-	-	-	-	-	-
B. cereus strain FSL K6-0040 | 1396.518	IV-Ia3	106	R	CTTGTACGGAGTGCGGTGAT	1	F	GTTACTCGCTTTAATCGTGGAC	0	absent	-	-	-	-	-	-
B. cereus G9842 | 405531.7	IV-Ia3	106	F	GTTACTCGCTTTAATCGTGGAC	0	R	CTTGTACGGAGTGCGGTGAT	0	absent	-	-	-	-	-	-
B. thuringiensis serovar thuringiensis str. T01001 | 527025.3	IV-Ia3	106	R	CTTGTACGGAGTGCGGTGAT	0	F	GTTACTCGCTTTAATCGTGGAC	0	absent	-	-	-	-	-	-
B. thuringiensis IBL 200 | 527019.3	IV-Ia3	106	R	CTTGTACGGAGTGCGGTGAT	1	F	GTTACTCGCTTTAATCGTGGAC	0	absent	-	-	-	-	-	-
B. thuringiensis IBL 4222 | 527020.3	IV-Ia3	106	R	CTTGTACGGAGTGCGGTGAT	0	F	GTTACTCGCTTTAATCGTGGAC	0	absent	-	-	-	-	-	-
B. bingmayongensis | 1150157.3	No affiliation	106	R	CTTGTACGGAGTGCGGTGAT	2	F	GTTACTCGCTTTAATCGTGGAC	0	absent	-	-	-	-	-	-
B. gaemokensis strain JCM 15801 | 574375.6	No affiliation	106	F	GTTACTCGCTTTAATCGTGGAC	2	R	CTTGTACGGAGTGCGGTGAT	3	absent	-	-	-	-	-	-
B. gaemokensis strain KCTC 13318 | 574375.8	No affiliation	106	F	GTTACTCGCTTTAATCGTGGAC	2	R	CTTGTACGGAGTGCGGTGAT	3	absent	-	-	-	-	-	-
B. manliponensis strain JCM 15802 | 574376.4	No affiliation	106	F	GTTACGCGCTTTAATCTTGGGC	3	R	CGTGCACAGAATGCGGTGAT	3	absent	-	-	-	-	-	-
B. cereus BAG1O-2 | 1053168.3	Va	106	R	CTTGTACGGAGTGCGGTGAT	0	F	GTTACTCGCTTTAATCGTGGAC	0	absent	-	-	-	-	-	-
B. cereus BAG2O-2 | 1053177.3	Va	106	F	GTTACTCGCTTTAATCGTGGAC	0	R	CTTGTACGGAGTGCGGTGAT	0	absent	-	-	-	-	-	-
B. cereus BAG4X2-1 | 1053187.3	Va	106	R	CTTGTACGGAGTGCGGTGAT	0	F	GTTACTCGCTTTAATCGTGGAC	0	absent	-	-	-	-	-	-
B. cereus BAG5O-1 | 1053188.3	Va	106	F	GTTACTCGCTTTAATCGTGGAC	0	R	CTTGTACGGAGTGCGGTGAT	0	absent	-	-	-	-	-	-
B. cereus BAG6O-1 | 1053192.3	Va	106	R	CTTGTACGGAGTGCGGTGAT	0	F	GTTACTCGCTTTAATCGTGGAC	0	absent	-	-	-	-	-	-
B. cereus HuA2-3 | 1053202.3	Va	106	F	GTTACTCGCTTTAATCGTGGAC	0	R	CTTGTACGGAGTGCGGTGAT	0	absent	-	-	-	-	-	-
B. cereus HuB2-9 | 1053209.3	Va	106	R	CTTGTACGGAGTGCGGTGAT	0	F	GTTACTCGCTTTAATCGTGGAC	0	absent	-	-	-	-	-	-
B. cereus HuB4-10 | 1053210.3	Va	106	F	GTTACTCGCTTTAATCGTGGAC	0	R	CTTGTACGGAGTGCGGTGAT	0	absent	-	-	-	-	-	-
B. cereus HuB5-5 | 1053212.3	Va	106	F	GTTACTCGCTTTAATCGTGGAC	0	R	CTTGTACGGAGTGCGGTGAT	0	absent	-	-	-	-	-	-
B. cereus VD115 | 1053230.3	Va	106	R	CTTGTACGGAGTGCGGTGAT	0	F	GTTACTCGCTTTAATCGTGGAC	0	absent	-	-	-	-	-	-
B. cereus VD131 | 1053232.3	Va	106	F	GTTACTCGCTTTAATCGTGGAC	0	R	CTTGTACGGAGTGCGGTGAT	0	absent	-	-	-	-	-	-
B. cereus VD148 | 1053237.3	Va	106	R	CTTGTACGGAGTGCGGTGAT	0	F	GTTACTCGCTTTAATCGTGGAC	0	absent	-	-	-	-	-	-
B. cereus VD214 | 1053245.3	Va	106	F	GTTACTCGCTTTAATCGTGGAC	0	R	CTTGTACGGAGTGCGGTGAT	0	absent	-	-	-	-	-	-
B. thuringiensis MC28 | 1195464.3	Va	106	F	GTTACTCGCTTTAATCGTGGAC	0	R	CTTGTACGGAGTGCGGTGAT	0	absent	-	-	-	-	-	-
B. toyonensis BCT-7112 | 1415784.4	Va	106	F	GTTACTCGCTTTAATCGTGGAC	0	R	CTTGTACGGAGTGCGGTGAT	0	absent	-	-	-	-	-	-
B. cereus strain 105Ne | 1396.424	Va	106	R	CTTGTACGGAGTGCGGTGAT	0	F	GTTACTCGCTTTAATCGTGGAC	0	absent	-	-	-	-	-	-
B. cereus strain IP5832 | 1396.450	Va	106	R	CTTGTACGGAGTGCGGTGAT	0	F	GTTACTCGCTTTAATCGTGGAC	0	absent	-	-	-	-	-	-
B. thuringiensis strain BAC3151 | 1428.319	Va	106	R	CTTGTACGGAGTGCGGTGAT	0	F	GTTACTCGCTTTAATCGTGGAC	0	absent	-	-	-	-	-	-
B. thuringiensis strain ZBG4 | 1428.318	Va	106	F	GTTACTCGCTTTAATCGTGGAC	0	R	CTTGTACGGAGTGCGGTGAT	0	absent	-	-	-	-	-	-
B. cereus strain FSL H8-0482 | 1396.515	Va	106	F	GTTACTCGCTTTAATCGTGGAC	0	R	CTTGTACGGAGTGCGGTGAT	0	absent	-	-	-	-	-	-
B. cereus strain FSL H8-0488 | 1396.503	Va	106	F	GTTACTCGCTTTAATCGTGGAC	0	R	CTTGTACGGAGTGCGGTGAT	0	absent	-	-	-	-	-	-
B. cereus Rock1-3 | 526981.3	Va	106	R	CTTGTACGGAGTGCGGTGAT	0	F	GTTACTCGCTTTAATCGTGGAC	0	absent	-	-	-	-	-	-
B. cereus Rock3-29 | 526984.3	Va	106	R	CTTGTACGGAGTGCGGTGAT	0	F	GTTACTCGCTTTAATCGTGGAC	0	absent	-	-	-	-	-	-
B. cereus Rock4-18 | 526988.3	Va	106	R	CTTGTACGGAGTGCGGTGAT	0	F	GTTACTCGCTTTAATCGTGGAC	0	absent	-	-	-	-	-	-
B. cereus BAG2X1-1 | 1053179.3	Vo	absent	-	-	-	-	-	-	absent	-	-	-	-	-	-
B. cereus BAG2X1-3 | 1053181.3	Vo	absent	-	-	-	-	-	-	absent	-	-	-	-	-	-
B. cereus VD142 | 718224.3	VIab	absent	-	-	-	-	-	-	198	F	CAAAACCAAAAACCATTAGCAA	1	R	GCAAGCAGCAAATGATGAGA	0
B. cereus BAG5X1-1 | 1053189.3	VIab	absent	-	-	-	-	-	-	198	R	GCAAGCAGCAAATGATGAGA	0	F	CAAAACCAAAAACCATTAGCAA	2
B. cereus BAG6O-2 | 1053193.3	VIab	absent	-	-	-	-	-	-	198	F	CAAAACCAAAAACCATTAGCAA	2	R	GCAAGCAGCAAATGATGAGA	0
B. cereus BtB2-4 | 1053197.3	VIab	absent	-	-	-	-	-	-	198	F	CAAAACCAAAAACCATTAGCAA	0	R	GCAAGCAGCAAATGATGAGA	0
B. cereus CER057 | 1053198.3	VIab	absent	-	-	-	-	-	-	198	F	CAAAACCAAAAACCATTAGCAA	0	R	GCAAGCAGCAAATGATGAGA	0
B. cereus CER074 | 1053199.3	VIab	absent	-	-	-	-	-	-	198	R	GCAAGCAGCAAATGATGAGA	0	F	CAAAACCAAAAACCATTAGCAA	0
B. cereus HuA2-1 | 1053201.3	VIab	absent	-	-	-	-	-	-	198	F	CAAAACCAAAAACCATTAGCAA	1	R	GCAAGCAGCAAATGATGAGA	0
B. cereus HuA2-4 | 1053203.3	VIab	absent	-	-	-	-	-	-	198	F	CAAAACCAAAAACCATTAGCAA	3	R	GCAAGCAGCAAATGATGAGA	0
B. cereus HuA2-9 | 1053204.3	VIab	absent	-	-	-	-	-	-	198	F	CAAAACCAAAAACCATTAGCAA	1	R	GCAAGCAGCAAATGATGAGA	0
B. cereus HuA3-9 | 1053205.3	VIab	absent	-	-	-	-	-	-	198	R	GCAAGCAGCAAATGATGAGA	0	F	CAAAACCAAAAACCATTAGCAA	2
B. cereus HuA4-10 | 1053206.3	VIab	absent	-	-	-	-	-	-	198	R	GCAAGCAGCAAATGATGAGA	0	F	CAAAACCAAAAACCATTAGCAA	3
B. cereus MC118 | 1053218.3	VIab	absent	-	-	-	-	-	-	198	F	CAAAACCAAAAACCATTAGCAA	2	R	GCAAGCAGCAAATGATGAGA	0
B. cereus MC67 | 1053219.3	VIab	absent	-	-	-	-	-	-	198	R	GCAAGCAGCAAATGATGAGA	0	F	CAAAACCAAAAACCATTAGCAA	2
B. cereus VD021 | 1053224.3	VIab	absent	-	-	-	-	-	-	198	R	GCAAGCAGCAAATGATGAGA	0	F	CAAAACCAAAAACCATTAGCAA	3
B. cereus VD048 | 1053226.3	VIab	absent	-	-	-	-	-	-	198	R	GCAAGCAGCAAATGATGAGA	0	F	CAAAACCAAAAACCATTAGCAA	0
B. cereus VD078 | 1053227.3	VIab	absent	-	-	-	-	-	-	198	R	GCAAGCAGCAAATGATGAGA	0	F	CAAAACCAAAAACCATTAGCAA	0
B. cereus VD118 | 1053231.3	VIab	absent	-	-	-	-	-	-	198	F	CAAAACCAAAAACCATTAGCAA	2	R	GCAAGCAGCAAATGATGAGA	0
B. cereus VD146 | 1053236.3	VIab	absent	-	-	-	-	-	-	198	F	CAAAACCAAAAACCATTAGCAA	0	R	GCAAGCAGCAAATGATGAGA	0
B. cereus VDM019 | 1053246.3	VIab	absent	-	-	-	-	-	-	198	F	CAAAACCAAAAACCATTAGCAA	0	R	GCAAGCAGCAAATGATGAGA	0
B. cereus VDM022 | 1053247.3	VIab	absent	-	-	-	-	-	-	198	R	GCAAGCAGCAAATGATGAGA	0	F	CAAAACCAAAAACCATTAGCAA	0
B. cereus VDM034 | 1053248.3	VIab	absent	-	-	-	-	-	-	198	F	CAAAACCAAAAACCATTAGCAA	2	R	GCAAGCAGCAAATGATGAGA	0
B. cereus VDM062 | 1053250.3	VIab	absent	-	-	-	-	-	-	198	R	GCAAGCAGCAAATGATGAGA	0	F	CAAAACCAAAAACCATTAGCAA	2
B. weihenstephanensis FSL H7-687 | 1227358.4	VIab	absent	-	-	-	-	-	-	198	F	CAAAACCAAAAACCATTAGCAA	0	R	GCAAGCAGCAAATGATGAGA	0
B. weihenstephanensis NBRC 101238 = DSM 11821 | 1220585.4	VIab	absent	-	-	-	-	-	-	198	F	CAAAACCAAAAACCATTAGCAA	1	R	GCAAGCAGCAAATGATGAGA	0
B. mycoides ATCC 6462 | 1405.10	VIab	absent	-	-	-	-	-	-	198	R	GCAAGCAGCAAATGATGAGA	0	F	CAAAACCAAAAACCATTAGCAA	0
B. weihenstephanensis strain WSBC 10204 | 86662.17	VIab	absent	-	-	-	-	-	-	198	R	GCAAGCAGCAAATGATGAGA	0	F	CAAAACCAAAAACCATTAGCAA	1
B. weihenstephanensis strain JAS 83/3 | 86662.6	VIab	absent	-	-	-	-	-	-	198	R	GCAAGCAGCAAATGATGAGA	0	F	CAAAACCAAAAACCATTAGCAA	0
B. mycoides strain B38V | 1405.14	VIab	absent	-	-	-	-	-	-	198	R	GCAAGCAGCAAATGATGAGA	0	F	CAAAACCAAAAACCATTAGCAA	2
B. mycoides strain WSBC 10969 | 1405.16	VIab	absent	-	-	-	-	-	-	198	R	GCAAGCAGCAAATGATGAGA	0	F	CAAAACCAAAAACCATTAGCAA	0
B. mycoides strain M2E_15 | 1405.18	VIab	absent	-	-	-	-	-	-	198	F	CAAAACCAAAAACCATTAGCAA	0	R	GCAAGCAGCAAATGATGAGA	0
B. cereus strain FSL H7-0926 | 1396.517	VIab	absent	-	-	-	-	-	-	198	R	GCAAGCAGCAAATGATGAGA	0	F	CAAAACCAAAAACCATTAGCAA	2
B. cereus strain FSL K6-0267 | 1396.512	VIab	absent	-	-	-	-	-	-	198	F	CAAAACCAAAAACCATTAGCAA	2	R	GCAAGCAGCAAATGATGAGA	0
B. mycoides strain PE8-15 | 1405.19	VIab	absent	-	-	-	-	-	-	198	F	CAAAACCAAAAACCATTAGCAA	2	R	GCAAGCAGCAAATGATGAGA	0
B. weihenstephanensis KBAB4 | 315730.11	VIab	absent	-	-	-	-	-	-	198	F	CAAAACCAAAAACCATTAGCAA	0	R	GCAAGCAGCAAATGATGAGA	0
B. cereus AH621 | 526972.3	VIab	absent	-	-	-	-	-	-	198	F	CAAAACCAAAAACCATTAGCAA	0	R	GCAAGCAGCAAATGATGAGA	0
B. cereus BDRD-ST196 | 526976.3	VIab	absent	-	-	-	-	-	-	198	F	CAAAACCAAAAACCATTAGCAA	1	R	GCAAGCAGCAAATGATGAGA	0
B. cereus AH603 | 526990.3	VIab	absent	-	-	-	-	-	-	198	R	GCAAGCAGCAAATGATGAGA	0	F	CAAAACCAAAAACCATTAGCAA	2
B. cereus BAG6X1-2 | 1053195.3	VIo1	absent	-	-	-	-	-	-	absent	-	-	-	-	-	-
B. cereus VD107 | 1053229.3	VIo1	106	F	GTCACTCGCTTTAATCGTGGAC	1	R	CTTGTACAGAATGTAGTGAT	3	absent	-	-	-	-	-	-
B. mycoides BHP | 1405.8	VIo2	106	F	GTTACTCGCTTTAATCGTGGAC	0	R	CTTGTACGGAGTGCGGTGAT	1	absent	-	-	-	-	-	-
B. cytotoxicus NVH 391-98 | 315749.8	VII	106	F	GTTACTCGCTTTAATCGTGGAC	0	R	CTTGTACGGAGTGCGGTGAT	3	absent	-	-	-	-	-	-
B. cytotoxicus strain CVUAS 2833 | 580165.3	VII	106	F	GTTACTCGCTTTAATCGTGGAC	0	R	CTTGTACAGAATGTGGAGAT	3	absent	-	-	-	-	-	-

[a], interpretation of the PCR results

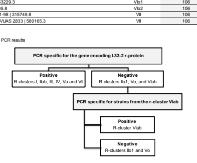

[b], confirmed deletion of the gene encoding for L33-2 r-protein
[c], uncertain status as the result of poor quality contigs (4447 contigs)

Table S5. In vitro PCR results for the *B. cereus* group members with the primers specific for (i) the gene encoding L33-2 r-protein and (ii) strains from the r-cluster VIab.

Strain [a]	MLST type	Phylogenetic group	Type of samples	Country	Year	References	PCR results	
							PCR specific for the gene encoding L33-2 r-protein	PCR specific for strains from the r-cluster VIab
B.c. 5958b [b]	26	III	Pasta salad	Belgium	2003	1	+	-
B.c. TIAC181 [b]	26	III	Fish and pastry	Belgium	2008	2	+	-
B.c. TIAC303 [b]	26	III	Pasta	Belgium	2008	3	+	-
B.c. A12 [b]	26	III	Hand of a woman	France	2004	2	+	-
B.c. BOD3/9 [b]	26	III	Fresh milk	Poland	2006	4	+	-
B.c. 2023 [b]	26	III	Pastry	Belgium	2006	2	+	-
B.c. I20-4 [b]	26	III	Ice cream	China	2006	5	+	-
B.c. 1757 [b]	26	III	Fried rice	Belgium	2011	2	+	-
B.c. CER072 [b]	26	III	Honey	Ivory Coast	2007	2	+	-
B.c. MHI1486 [b]	26	III	Food poisoning	Germany	2006	6	+	-
B.c. MHI1699 [b]	26	III	Food poisoning	Germany	2006	6	+	-
B.c. H308197 [b]	144	III	Rice	USA	1997	7	+	-
B.c. AND1407 [b]	164	III	Blackcurrant	Denmark	2009	8	+	-
B.c. F4810/72 [b]	26	III	Vomit	United Kingdom	1979	9	+	-
B.c. BB 01-3	295	II	Biebrza National Park	Poland	2013	10	+	-
B.c. BB 18-3	700	II	Biebrza National Park	Poland	2013	10	+	-
B.c. BB 19-4	632	II	Biebrza National Park	Poland	2013	10	+	-
B.c. BB 20-4	686	II	Biebrza National Park	Poland	2013	10	+	-
B.c. BB 25-1	551	II	Biebrza National Park	Poland	2013	10	+	-
B.c. BB 26-4	564	II	Biebrza National Park	Poland	2013	10	+	-
B.c. BB 50-5	632	II	Biebrza National Park	Poland	2013	10	+	-
B.c. BB 51-4	644	II	Biebrza National Park	Poland	2013	10	+	-
B.c. BB 54-3	295	II	Biebrza National Park	Poland	2013	10	+	-
B.c. BB 54-4	644	II	Biebrza National Park	Poland	2013	10	+	-
B.c. JAS 05-4	312	II	Jasienowka farm	Poland	2013	10	+	-
B.c. JAS 07-5	312	II	Jasienowka farm	Poland	2013	10	+	-
B.c. JAS 08-1	682	II	Jasienowka farm	Poland	2013	10	+	-
B.c. JAS 11-1	682	II	Jasienowka farm	Poland	2013	10	+	-
B.c. JAS 28-2	613	II	Jasienowka farm	Poland	2013	10	+	-
B.c. JAS 31-2	614	II	Jasienowka farm	Poland	2013	10	+	-
B.c. JAS 90-2	294	II	Jasienowka farm	Poland	2013	10	+	-
B.t. IS5056	10	IV	Biebrza National Park	Poland	2008	11	+	-
B.t. HD3	171	III	Bacillus Genetics Stock Center; www.bgsc.org				+	-
B.t. HD30	832	III	Bacillus Genetics Stock Center; www.bgsc.org				+	-
B.t. HD1	8	IV	Bacillus Genetics Stock Center; www.bgsc.org				+	-
B.t. HD73	8	IV	Bacillus Genetics Stock Center; www.bgsc.org				+	-
B.t. HD112	833	IV	Bacillus Genetics Stock Center; www.bgsc.org				+	-
B.t. HD125	840	IV	Bacillus Genetics Stock Center; www.bgsc.org				+	-
B.t. HD133	834	IV	Bacillus Genetics Stock Center; www.bgsc.org				+	-
B.t. HD146	56	IV	Bacillus Genetics Stock Center; www.bgsc.org				+	-
B.t. HD541	838	V	Bacillus Genetics Stock Center; www.bgsc.org				+	-
B.t. HD542	830	IV	Bacillus Genetics Stock Center; www.bgsc.org				+	-
ATCC 10987	32	III	American Type Culture Collection; www.atcc.or				+	-
ATCC 14579	4	IV	American Type Culture Collection; www.atcc.or				+	-
B.w. BB 22/2	339	VI	Biebrza National Park	Poland	2013	10	-	+
B.w. BB 29/2	635	VI	Biebrza National Park	Poland	2013	10	-	+
B.w. BB 33/2	409	VI	Biebrza National Park	Poland	2013	10	-	+
B.w. BB 52/3	421	VI	Biebrza National Park	Poland	2013	10	-	+
B.w. BB 53-3	728	VI	Biebrza National Park	Poland	2013	10	-	+
B.w. BPN 08/4	649	VI	Bialowieza National Park	Poland	2013	10	-	+
B.w. BPN 21/1	729	VI	Bialowieza National Park	Poland	2013	10	-	+
B.w. BPN 28/4	651	VI	Bialowieza National Park	Poland	2013	10	-	+
B.w. BPN 30/1	736	VI	Bialowieza National Park	Poland	2013	10	-	+
B.w. BPN 34/4	736	VI	Bialowieza National Park	Poland	2013	10	-	+
B.w. BPN 35/4	654	VI	Bialowieza National Park	Poland	2013	10	-	+
B.w. BPN 36/2	650	VI	Bialowieza National Park	Poland	2013	10	-	+
B.w. BPN 36/3	742	VI	Bialowieza National Park	Poland	2013	10	-	+
B.w. BPN 37/1	655	VI	Bialowieza National Park	Poland	2013	10	-	+
B.w. BPN 38/2	674	VI	Bialowieza National Park	Poland	2013	10	-	+
B.w. BPN 42/1	730	VI	Bialowieza National Park	Poland	2013	10	-	+
B.w. BPN 43/4	656	VI	Bialowieza National Park	Poland	2013	10	-	+
B.w. BPN 50/3	657	VI	Bialowieza National Park	Poland	2013	10	-	+
B.w. BPN 52/2	657	VI	Bialowieza National Park	Poland	2013	10	-	+
B.w. BPN 53/1	692	VI	Bialowieza National Park	Poland	2013	10	-	+
B.w. BPN 54/1	731	VI	Bialowieza National Park	Poland	2013	10	-	+
B.w. BPN 55/4	656	VI	Bialowieza National Park	Poland	2013	10	-	+
B.w. BPN 60/1	624	VI	Bialowieza National Park	Poland	2013	10	-	+
B.w. JAS 01/4	681	VI	Jasienowka farm	Poland	2013	10	-	+
B.w. JAS 06/1	222	VI	Jasienowka farm	Poland	2013	10	-	+
B.w. JAS 15/2	222	VI	Jasienowka farm	Poland	2013	10	-	+
B.w. JAS 19/1	566	VI	Jasienowka farm	Poland	2013	10	-	+
B.w. JAS 21/1	566	VI	Jasienowka farm	Poland	2013	10	-	+
B.w. JAS 23/1	727	VI	Jasienowka farm	Poland	2013	10	-	+
B.w. JAS 26/2	619	VI	Jasienowka farm	Poland	2013	10	-	+
B.w. JAS 30/3	735	VI	Jasienowka farm	Poland	2013	10	-	+
B.w. JAS 68/2	727	VI	Jasienowka farm	Poland	2013	10	-	+
B.w. JAS 74/3	617	VI	Jasienowka farm	Poland	2013	10	-	+
B.w. JAS 76/3	698	VI	Jasienowka farm	Poland	2013	10	-	+
B.w. JAS 78/2	734	VI	Jasienowka farm	Poland	2013	10	-	+
B.w. JAS 80/5	683	VI	Jasienowka farm	Poland	2013	10	-	+
B.w. JAS 82/3	719	VI	Jasienowka farm	Poland	2013	10	-	+
B.w. JAS 84/1	684	VI	Jasienowka farm	Poland	2013	10	-	+
B.w. JAS 84/4	650	VI	Jasienowka farm	Poland	2013	10	-	+
B.w. JAS 85/1	410	VI	Jasienowka farm	Poland	2013	10	-	+
B.w. JAS 94/5	726	VI	Jasienowka farm	Poland	2013	10	-	+
B.m. BPN 1	670	VI	Bialowieza National Park	Poland	2013	10	-	+
B.m. BPN 4	670	VI	Bialowieza National Park	Poland	2013	10	-	+
B.m. BPN 5	712	VI	Bialowieza National Park	Poland	2013	10	-	+
B.m. BPN 8	625	VI	Bialowieza National Park	Poland	2013	10	-	+
B.m. BPN 9	625	VI	Bialowieza National Park	Poland	2013	10	-	+
B.m. BPN 12	624	VI	Bialowieza National Park	Poland	2013	10	-	+
B.m. BPN 13	222	VI	Bialowieza National Park	Poland	2013	10	-	+
B.m. BPN 15	712	VI	Bialowieza National Park	Poland	2013	10	-	+
B.w. CER074	410	VI	Raw milk	Belgium	2007	12	-	+
B.w. MC118	1012	VI	Soil	Denmark	2006	13	-	+

[a], *B.c.*, *B. cereus*; *B.w.*, *B. weihenstephanensis*; *B.m.*, *B. mycoides*; *B.t.*, *B. thuringiensis.*

[b], Emetic *B. cereus* strains.

References

1. Dierick, K., Van Coillie, E., Swiecicka, I., Meyfroidt, G., Devlieger, H., Meulemans, A., Hoedemaekers, G., Fourie, L., Heyndrickx, M., Mahillon, J., *J. Clin. Microbiol*. 2005, *43*, 4277-4279.
2. Castiaux, V., N'guessan, E., Swiecicka, I., Delbrassinne, L., Dierick, K., Mahillon, J., *FEMS Microbiol. Lett.* 2014, *353*, 124-131.
3. Naranjo, M., Denayer, S., Botteldoorn, N., Delbrassinne, L., Veys, J., Waegenaere, J., Sirtaine, N., Driesen, R.B., Sipido, K.R., Mahillon, J., Dierick, K., *J. Clin. Microbiol.* 2011, *49*, 4379-4381.
4. Bartoszewicz, M., Kroten, M., Swiecicka, I., *Folia Microbiol.* 2013, *58*, 529-535.
5. Zhou, G., Zheng, D., Dou, L., Cai, Q., Yuan, Z., *Int. J. Food Microbiol.* 2010, *137*, 143-146.
6. Moravek, M., Dietrich, R., Buerk, C., Broussolle, V., Guinebretière, M.H., Granum, P.E., Nguyen-The, Ch., Märtlbauer, E., *FEMS Microbiol. Lett.* 2006, *257*, 293-298.
7. Hoffmaster, A.R., Novak, R.T., Marston, C.K., Gee, J.E., Helsel, L., Pruckler, J.M., Wilkins, P.P., *BMC Microbiol.*, 2008, *8*, 191.
8. Hoton, F.M., Fornelos, N., N'Guessan, E., Hu, X., Swiecicka, I., Dierick, K., Jääskeläinen, E., Salkinoja-Salonen, M., Mahillon, J., *Environ. Microbiol. Rep.* 2009, *1*, 177–183.
9. Turnbull, P.C.B., Kramert, J.M., Jørgensen, K., Gilbert, R.J., Melling, J., *Am. J. Clin. Nutr.* 1979, *32*, 219-228.
10. Drewnowska, J.M., Swiecicka, I., *PLoS One*, 2013, *8*, e80175.
11. Swiecicka, I., Bideshi, D.K., Federici, B.A., *Appl. Environ. Microbiol*. 2008, *74*, 923-930.
12. Castiaux, V., N'Guessan, E., Swiecicka, I., Delbrassinne, L., Dierick, K., Mahillon, J., *FEMS Microbiol. Lett.*, 2014, *353*, 124-131.
13. Thorsen, L., Hansen, *B.M.*, Nielsen, K.F., Hendriksen, N.B., Phipps, R.K., Budde, B.B., *Appl. Environ. Microbiol. 72*, 5118-5121.

Table S6. The r-protein variants in the *B. cereus* group.

[illegible]

Table S7. Distribution of amino acids in the 54 r-proteins (6808 amino acids) among the r-clusters of the *B. cereus* group thermotypes.

	R-cluster											
	Thermotolerant	Mesophilic						Intermediate		Psychrotolerant		
**Amino acid**	**VII** (n=2) [a]	**I** (n=8)	**IIIc** (n=50)	**IIIb** (n=108)	**IIIa** (n=9)	**IV-M** (n=77)	**IV-I** (n=37)	**Vo** (n=2)	**Va** (n=24)	**IIo2** (n=4)	**IIa-c** (n=16)	**VIab** (n=37)
Alanine (A)	597	597	596	594	596	592	591	594	593	585	590	584
Cysteine (C)	26	26	26	26	26	26	26	26	26	26	26	27
Aspartic Acid (D)	261	269	267	267	267	266	265	263	263	264	261	262
Glutamic Acid (E)	510	496	499	497	498	499	499	495	500	499	500	495
Phenylalanine (F)	175	178	176	176	176	175	175	175	175	175	175	174
Glycine (G)	544	546	549	549	548	548	547	549	548	549	548	548
Histidine (H)	124	122	124	123	123	123	123	123	123	123	122	123
Isoleucine (I)	399	396	395	396	399	399	402	402	403	402	404	405
Lysine (K)	734	728	727	730	730	730	730	729	732	728	729	726
Leucine (L)	483	479	484	484	484	485	483	481	482	482	480	480
Methionine (M)	180	179	178	177	177	178	179	177	179	178	178	179
Asparagine (N)	275	272	274	273	272	270	269	270	268	269	275	275
Proline (P)	238	236	235	234	235	237	237	236	236	236	236	236
Glutamine (Q)	204	208	210	211	211	211	209	210	208	210	209	211
Arginine (R)	559	560	561	560	560	561	562	562	561	561	562	562
Serine (S)	284	295	290	294	292	293	300	302	302	303	301	303
Threonine (T)	390	397	395	395	395	396	393	396	394	396	395	399
Valine (V)	649	650	648	648	646	644	643	644	639	646	642	643
Tryptophan (W)	29	29	29	29	29	29	29	29	29	29	29	29
Tyrosine (Y)	146	146	145	145	146	146	147	147	147	147	147	148

[a], the number of strains used to calculate the mean number of distribution of amino acids

Table S8. Prevalence of substitution of Isoleucine (I) for Valine (V) in r-proteins among the *B. cereus s.l.* r-clusters I, II, III, IV, V and VII in comparison to the most psychrotolerant r-cluster VI.

		**R-cluster**											
		**Thermotolerant**		**Mesophilic**				**Intermediate**			**Psychrotolerant**		
**R-protein**	**I→V position**	**VII** (n=2)[a]	**I** (n=8)	**IIIc** (n=50)	**IIIb** (n=108)	**IIIa** (n=9)	**IV-M** (n=77)	**IV-I** (n=37)	**Vo** (n=2)	**Va** (n=24)	**IIo2** (n=4)	**IIa-c** (n=16)	**VIab** (n=37)
L2	192	100%	100%	100%	100%	100%	-	-	-	-	-	-	-
L6	46	100%	-	-	-	-	-	-	-	-	-	-	-
L6	132	100%	-	-	-	-	-	-	-	-	-	-	-
L6	134	100%	-	-	-	-	-	-	-	-	-	-	-
L7/L12	93	-	62.5%										
L9	18	-	-	100%	100%	100%	100%	70.3%	100%	-	-	-	-
L13	63	100%	-	-	-	-	-	-	-	-	-	-	-
L15	111	100%	75%	-	-	-	100%	-	-	-	-	-	-
L16	27	100%	-	98%	-	-	-	-	-	-	-	-	-
L16	102	100%	-	-	-	-	-	-	-	-	-	-	-
L19	64	100%	-	-	-	-	-	-	-	-	-	-	-
L22	66	100%	-	-	-	-	-	-	-	-	-	-	-
L23	43	100%	62.5%	-	-	-	-	-	-	-	-	-	-
L30	55	100%	100%	100%	100%	100%	100%	-	-	-	-	-	-
L31	48	100%	100%	100%	100%	100%	100%	100%	-	100%	-	-	-
S1	72	100%	100%	90%	100%	100%	-	-	100%	-	-	-	-
S1	191	-	100%	100%	100%	100%	100%	100%	100%	-	100%	100%	11%
S1	271	-	100%	100%	99.1%	11.1%	-	-	-	-	-	-	-
S1	276	100%											
S2	163	100%	100%	100%	100%	100%	100%	100%	100%	100%	100%	88.9%	24%
S3	8	100%											
S3	14	100%	100%	100%	100%	100%	100%	100%	100%	100%	100%	100%	-
S6	72	100%	100%	-	-	-	-	-	-	-	-	-	-
S10	48	100%	100%	100%	100%	100%	100%	-	-	-	-	-	-
S11	20	100%	-	100%	100%	77.8%	-	-	-	-	-	-	-
S13	39	100%	100%	100%	-	-	-	-	-	-	-	-	-
S13	59	-	-	-	-	-	-	100%	100%	100%	-	-	-
S13	60	-	100%	-	-	-	-	-	-	-	-	-	-
S13	63	-	100%	-	-	-	-	-	-	-	-	-	-
S19	62	-	-	100%	100%	100%	-	-	-	-	-	-	-

[a], the number of strains used to obtain the consensus sequence

Table S9. Prevalence of substitution of Serine (S) for Adenine (A) in r-proteins among the *B. cereus s.l.* r-clusters I, II, III, IV, V and VII in comparison to the most psychrotolerant r-cluster VI.

		R-cluster											
		Thermotolerant		Mesophilic				Intermediate			Psychrotolerant		
**R-protein**	**S→A position**	**VII** (n=2) [a]	**I** (n=8)	**IIIc** (n=50)	**IIIb** (n=108)	**IIIa** (n=9)	**IV-M** (n=77)	**IV-I** (n=37)	**Vo** (n=2)	**Va** (n=24)	**IIo2** (n=4)	**IIa-c** (n=16)	**VIab** (n=37)
L1	19	- [b]	-	100%	100%	100%	100%	49%	100%	- [b]	100%	- [b]	-
L2	186	100%	-	-	-	-	-	-	-	-	-	-	-
L2	239	100%	-	-	-	-	-	-	-	-	-	-	-
L4	162	100%	62.5%	-	-	-	-	-	-	-	-	-	-
L9	105	- [b]	100%	-	-	-	-	-	-	-	-	-	-
L10	36	-	62.5%	-	-	-	-	-	-	-	-	-	-
L13	30	100%	100%	100%	100%	100%	100%	5.4%	100%	-	-	-	-
L15	80	100%	100%	-	-	-	-	-	-	-	-	-	-
L23	34	100%	-	-	-	-	-	-	-	-	-	-	-
L30	34	100%	-	-	-	-	-	-	-	-	-	-	-
S4	183	100%	100%	96%	100%	78%	18.2%	100%	-	-	-	-	-
S6	67	-	-	74%	100%	-	-	-	-	-	-	-	-
S6	69	- [c]	100%	100%	100%	100%	100%	100%	-	100%	-	100%	5.4%
S7	54	- [b]	100%	- [b]	- [b]	- [b]	- [b]	-	-	-	-	-	-
S9	84	100%	-	-	-	-	-	-	-	-	-	-	-
S11	71	100%	100%	100%	100%	100%	100%	100%	100%	100%	100%	100%	-
S18	19	100%	-	100%	-	100%	100%	100%	100%	-	-	18.7%	-
S20	19	100%	100%	100%	100%	100%	100%	100%	-	100%	100%	-	-

[a], the number of strains used to obtain the consensus sequence

[b], S→T substitution

[c], S→P substitution

Table S10. Prevalence of substitution of Serine (S) for Threonine (T) in r-proteins among the *B. cereus s.l.* r-clusters I, II, III, IV, V and VII in comparison to the most psychrotolerant r-cluster VI.

		R-cluster											
		Thermotolerant	Mesophilic					Psychrotolerant					
**R-protein**	**T→A position**	**VII** (n=2)[a]	**I** (n=8)	**IIIc** (n=50)	**IIIb** (n=108)	**IIIa** (n=9)	**IV-M** (n=77)	**IV-I** (n=37)	**Vo** (n=2)	**Va** (n=24)	**IIo2** (n=4)	**IIa-c** (n=16)	**VIab** (n=37)
L3	61	-	-	-	-	-	-	-	-	-	-	-	-
L3	75	100%	-	-	-	-	-	-	-	-	6.25%	-	-
L3	181	-[b]	100%	-	-	-	-	-	-	-	-	-	-
L4	147		-	-	-	-	-	-	-	-	-	-	-
L6	40	100%	100%	100%	100%	100%	100%	100%	100%	100%	100%	100%	-
L6	82	-	-	-[c]	-[c]	-[c]	-[c]	100%	-	-	100%	12.5%	-
L7/L12	98	-[d]	-[d]	100%	100%	100%	100%	48.6%	-[d]	-[d]	-[d]	-[d]	-
L15	75	100%	50%	100%	100%	100%	100%	-	-	-	-	12.5%	-
L15	81	100%	-	-	-	-	-	-	-	-	-	-	-
L16	25	-	100%	-	-	-	-	-	-	-	-	-	-
L19	6	100%	100%	100%	100%	100%	100%	100%	100%	100%	100%	100%	-
S1	345	100%	100%	98% [e]	100%	100%	100%	97% [e]	100%	100%	-	31.2%	-
S2	224	100%	100%	100%	100%	100%	100%	100%	100%	100%	100%	100%	5.4% [f]
S13	35	100%	100%	100%	100%	100%	100%	-	-	-	-	-	-
S20	37	100%	100%	100%	100%	100%	100%	100%	100%	100%	100%	100%	2.7%

[a], the number of strains used to obtain the consensus sequence
[b], T→V substitution
[c], T→E substitution
[d], T→S substitution
[e], as the result of T→V substitution in on strain
[f], in addition T→S substitution in seven strains

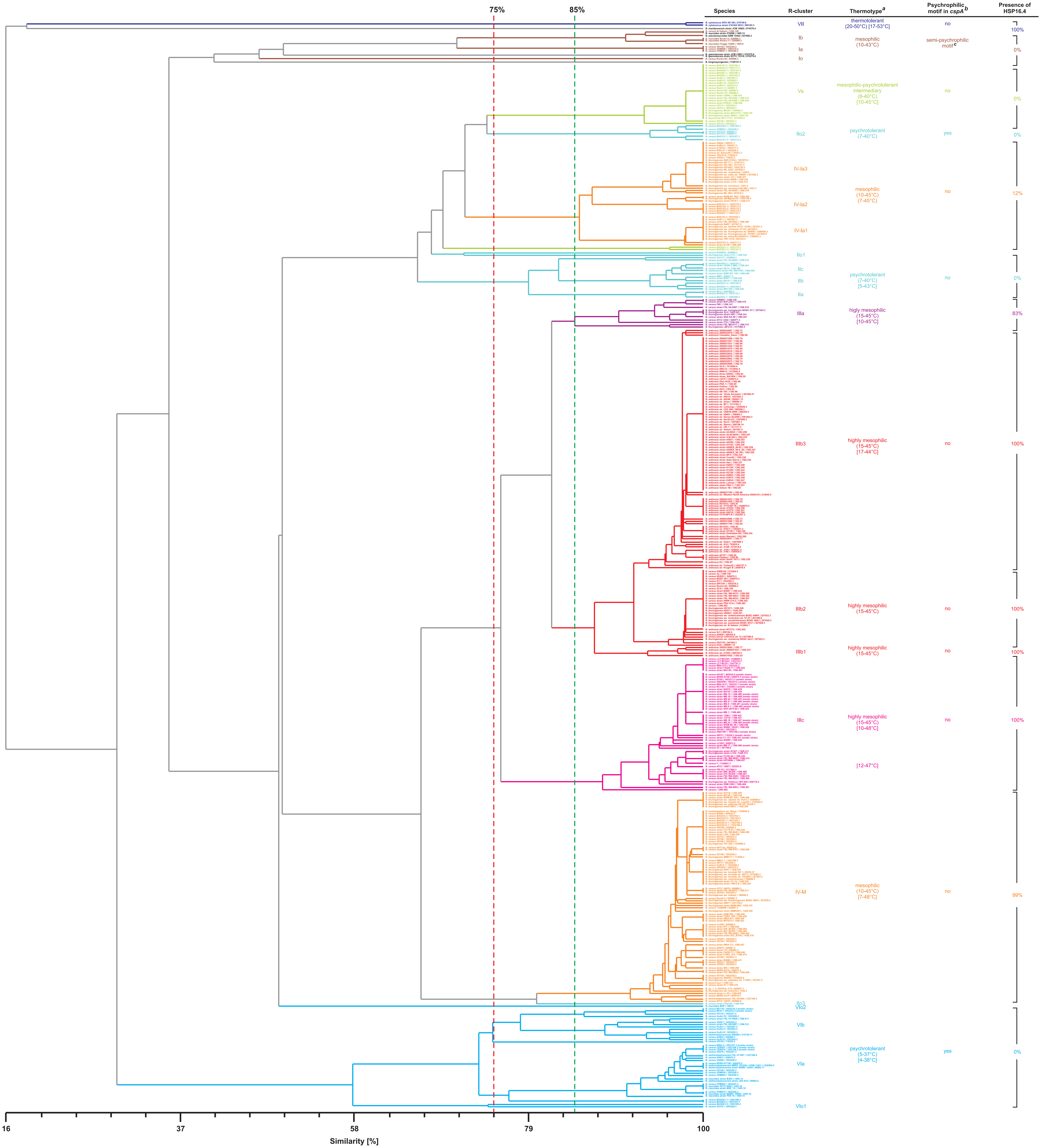

a, thermotypes and the thermal growth limits (round brackets) were assigned based on the strains' affiliation to phylogenetic group (I-VII) by Guinebretière et al. [2008][20]; temperatures in square brackets represent the limits recognized in this work and in other studies for certain strains under the study (see Supplementary Table S2)

b, psychrotolerant motif in *cspA* ([4]**A**CAGT**T**)

c, semi-psychrotolerant motif in *cspA* ([4]**A**CAGTA)

Figure S1. Phyloproteomic tree of the *B. cereus* group constructed based on the comparison of the 55 r-proteins using the Dice coefficient and UPGMA clustering with NTSYSpc 2.1 software. The analysis involved 421 strains.

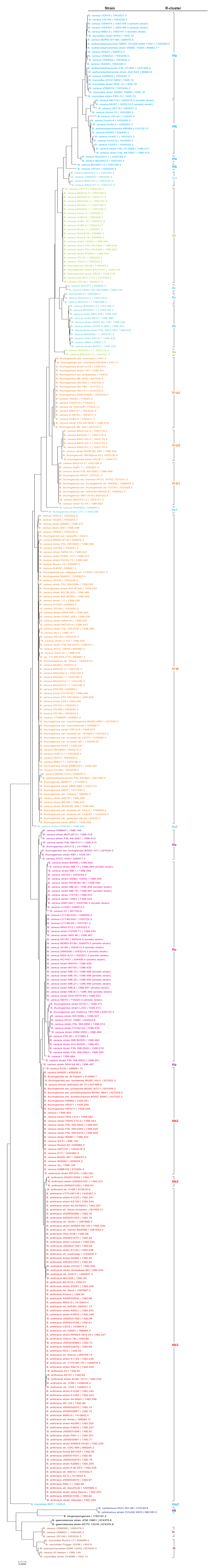

Figure S2. Phyloproteomic tree of the *B. cereus* group prepared based on concatenated sequences of the 54 r-proteins (6808 amino acids) using the Neighbor-Joining with MEGA7 software. The analysis involved 383 strains, i.e. all strains with the complete amino acid sequences for the 54 r-proteins. The evolutionary distances were computed using the Poisson correction method.

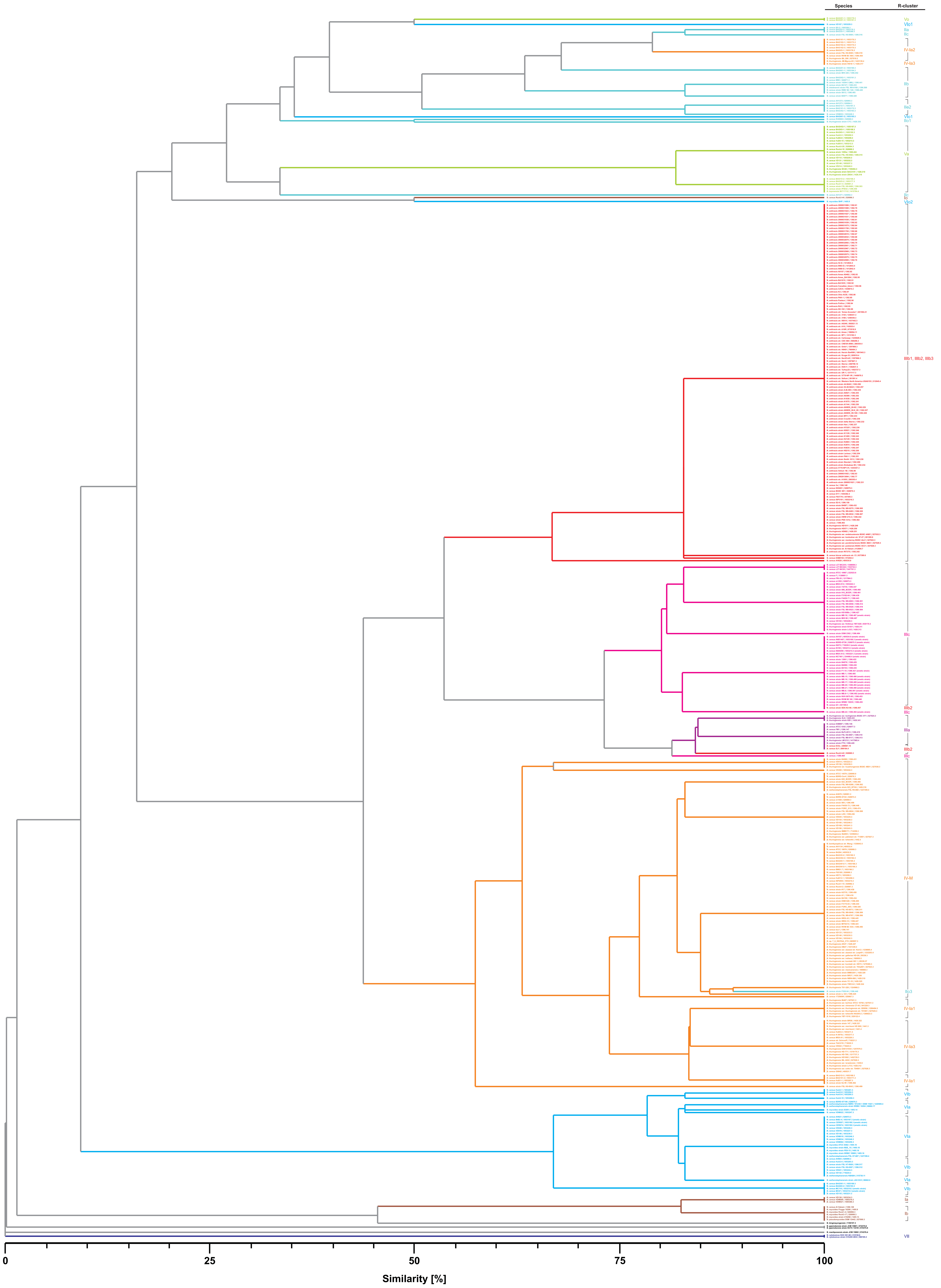

Figure. S3. Phyloproteomic tree of the *B. cereus* group built based on the comparison of the six CSPs using the Dice coefficient and UPGMA clustering with NTSYSpc 2.1 software. The analysis involved 421 strains.

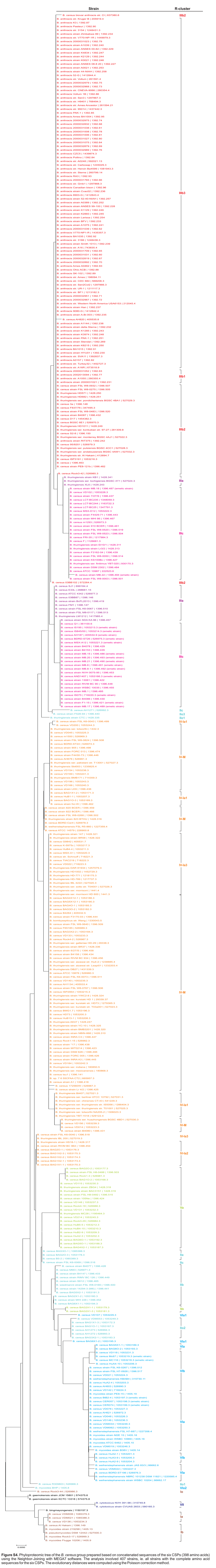

Figure S4. Phyloproteomic tree of the *B. cereus* group prepared based on concatenated sequences of the six CSPs (398 amino acids) using the Neighbor-Joining with MEGA7 software. The analysis involved 407 strains, ie. all strains with the complete amino acid sequences for the six CSPs. The evolutionary distances were computed using the Poisson correction method.

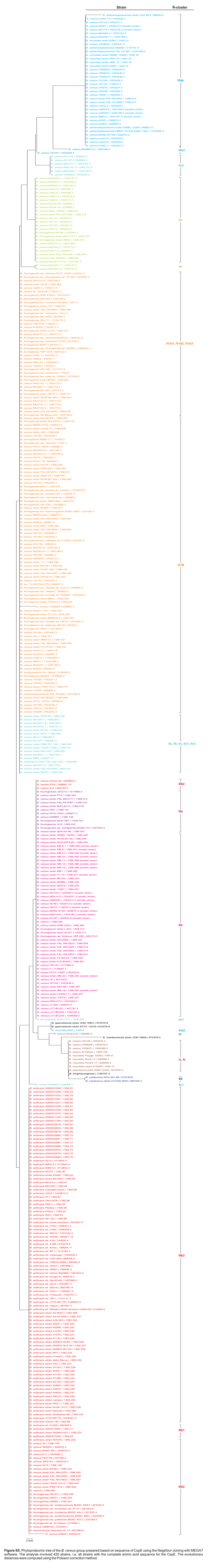

Figure S5. Phyloproteomic tree of the *B. cereus* group prepared based on sequence of CspE using the Neighbor-Joining with MEGA7 software. The analysis involved 420 strains, i.e. all strains with the complete amino acid sequence for the CspE. The evolutionary distances were computed using the Poisson correction method.

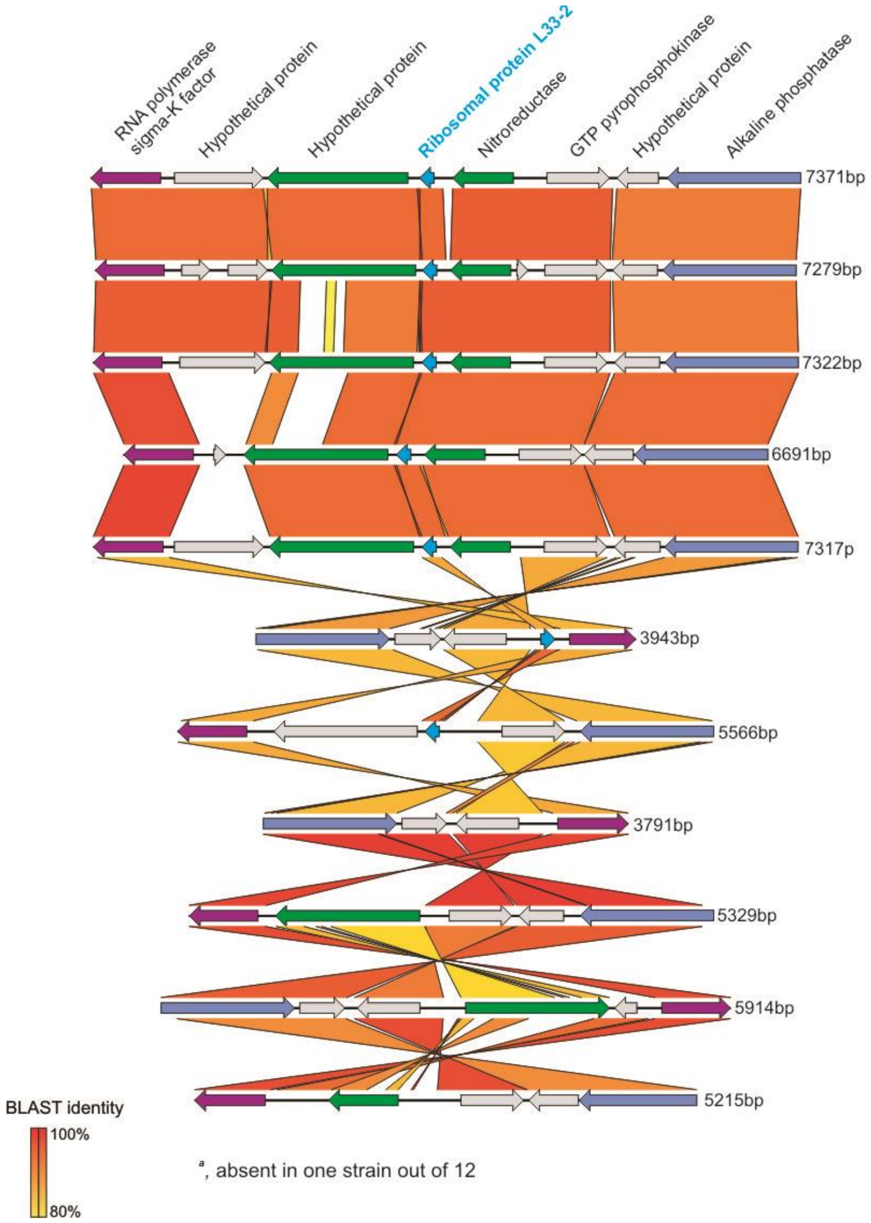

R-cluster	Presence of L33-2	Used strain
IV-M	100%	*B. cereus* ATCC 14579 | 226900.8
IIIb	100%	*B. anthracis* str. A0248 | 592021.13
IV-I	100%	*B. thuringiensis* ser. *thuringiensis* str. IS5056 | 1286404.3
Va	100%	*B. thuringiensis* MC28 | 1195464.3
IIb	100%	*B. cereus* MM3 | 526971.3
I	100%	*B. pseudomycoides* DSM 12442 | 527000.3
VII	100%	*B. cytotoxicus* NVH 391-98 | 315749.8
IIo2	0%	*B. cereus* AH1273 | 526994.3
VI	0%	*B. weihenstephanensis* KBAB4 | 315730.11
Vo	0%	*B. cereus* BAG2X1-1 | 1053179.3
IIIa[a]	92%	*B. cereus* strain FT9 | 1396.420

[a], absent in one strain out of 12

Figure S6. Genomic environment and prevalence of gene encoding for L33-2 r-protein among the r-clusters of the *B. cereus* group. The figure was prepared using Easyfig software[61].

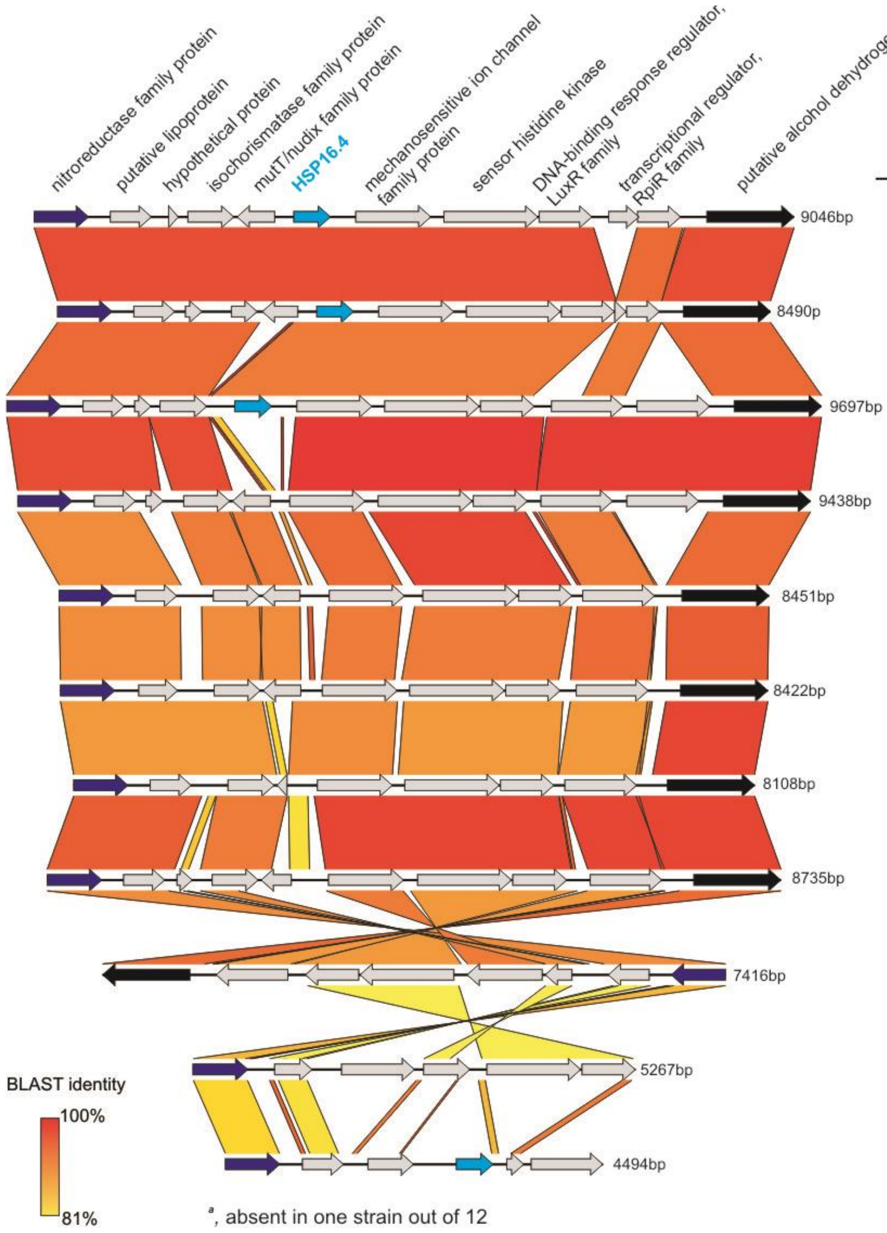

R-cluster	Presence of HSP16.4	Used strain
IIIc	100%	*B. cereus* AH187 (emetic strain) | 405534.9
IIIb	100%	*B. anthracis* str. A0248 | 592021.13
IV-M	100%	*B. cereus* ATCC 14579 | 226900.8
IV-I	12%	*B. thuringiensis* ser. *thuringiensis* str. IS5056 | 1286404.3
Va	0%	*B. thuringiensis* MC28 | 1195464.3
IIb	0%	*B. cereus MM3* | 526971.3
IIo2	0%	*B. cereus* AH1273 | 526994.3
VI	0%	*B. weihenstephanensis* KBAB4 | 315730.11
IIIa[a]	88%	*B. cereus strain* 03BB87 | 315730.11
I	0%	*B. pseudomycoides* DSM 12442 | 527000.3
VII	100%	*B. cytotoxicus* NVH 391-98 | 315749.8

Figure S7. Genomic environment and prevalence of gene encoding for HSP16.4 protein among the r-clusters of the *B. cereus* group. The figure was prepared using Easyfig software[61].

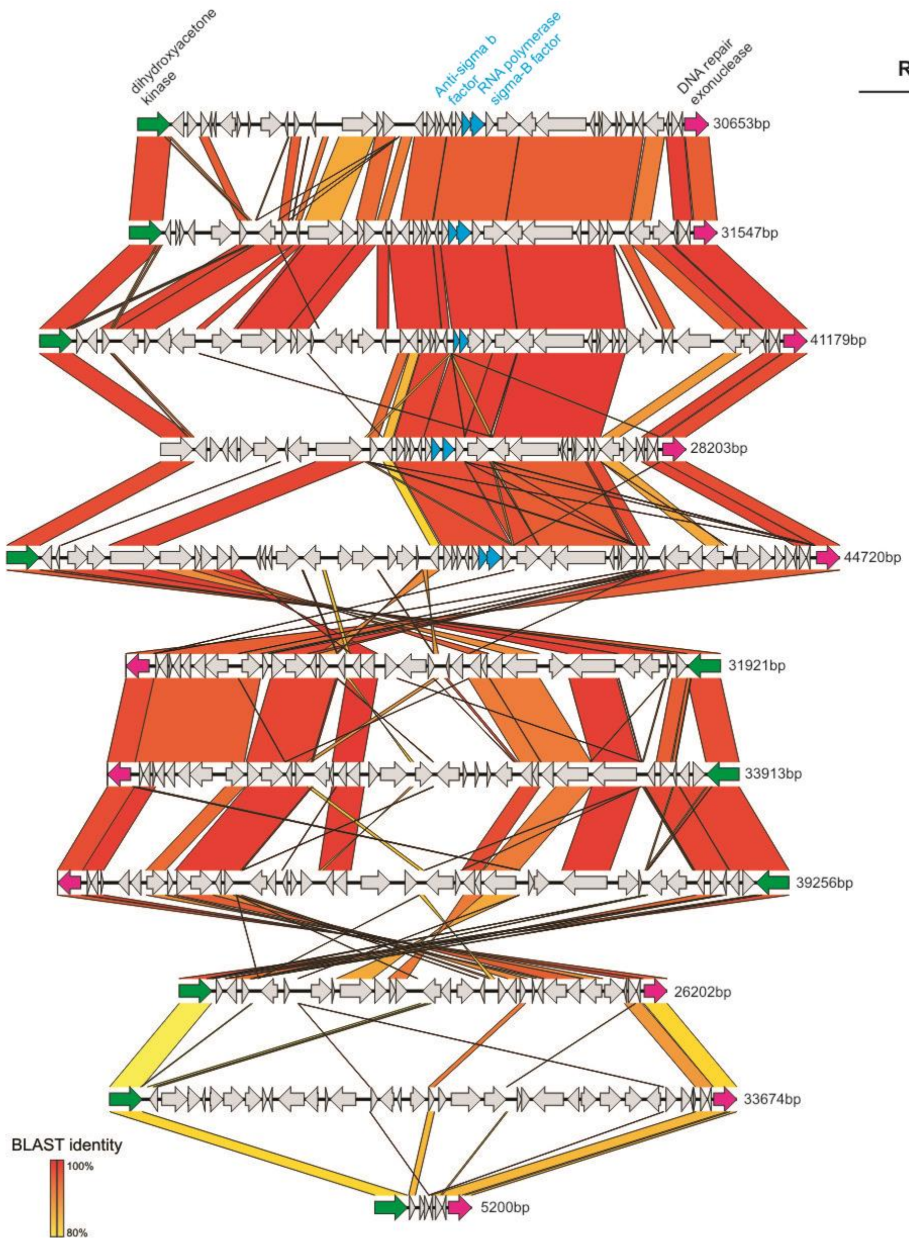

R-cluster	Presence of Anti-sigma b & RNA polymerase sigma-B factors	Used strain
IIIb	100%	*B. anthracis* str. A0248 | 592021.13
IV-M	100%	*B. cereus* ATCC 14579 | 226900.8
IV-Ia1	100%	*B. thuringiensis* ser. *thuringiensis* str. IS5056 | 1286404.3
Va	96%	*B. thuringiensis* MC28 | 1195464.3
VIb	29%	*B. weihenstephanensis* KBAB4 | 315730.11
VIb		*B. cereus* HuA4-10 | 1053206.3]
VIa	75%	*B. cereus* CER074 | 1053199.3]
IIo2	33%	*B. cereus* BAG1X1-3 | 1053172.3]
IV-Ia2	37%	*B. cereus* BAG2O-1 | 1053176.3]
I	0%	*B. pseudomycoides* DSM 12442 | 527000.3
VII	0%	*B. cytotoxicus* NVH 391-98 | 315749.8

Figure S8. Genomic environment and prevalence of the operon encoding for RNA polymerase sigma-B and anti-sigma-B factors among the r-clusters of the *B. cereus* group. The figure was prepared using Easyfig software[61].

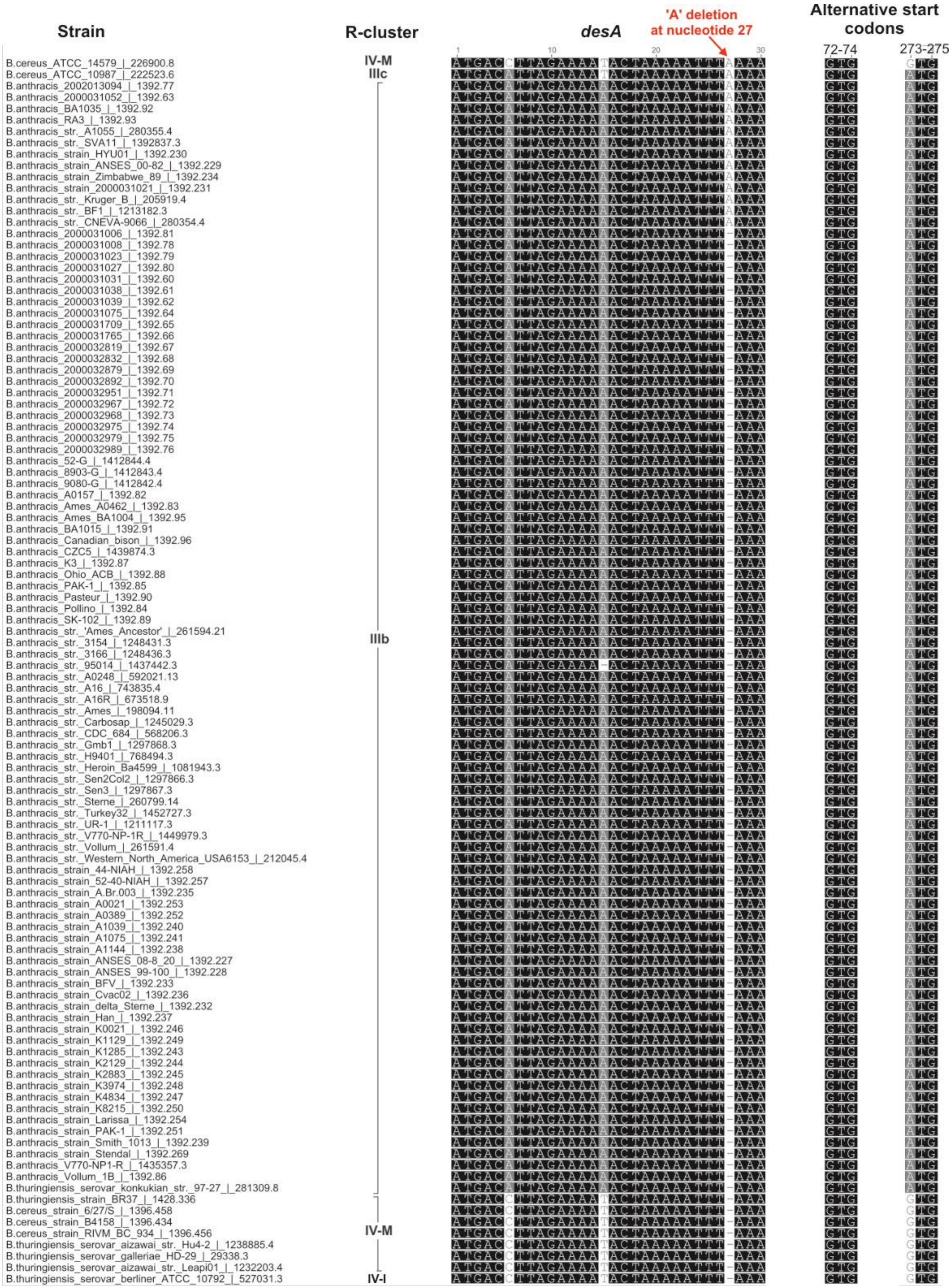

Figure S9. Frameshift mutation in *desA* among *B. anthracis* strains and the remaining species of the *B. cereus* group.

A

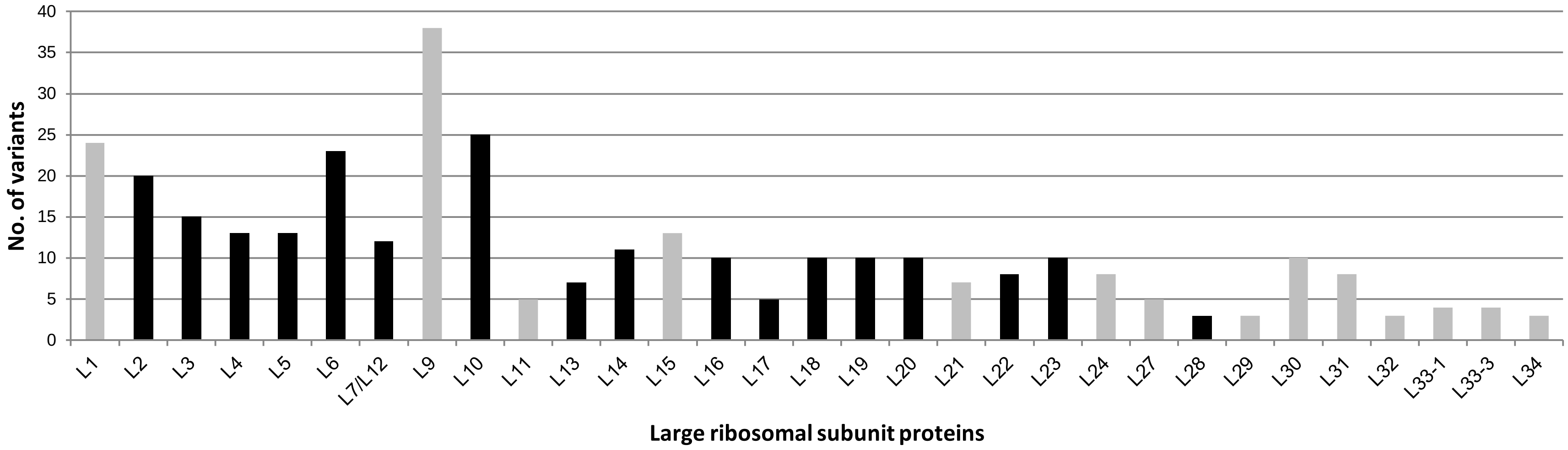

B

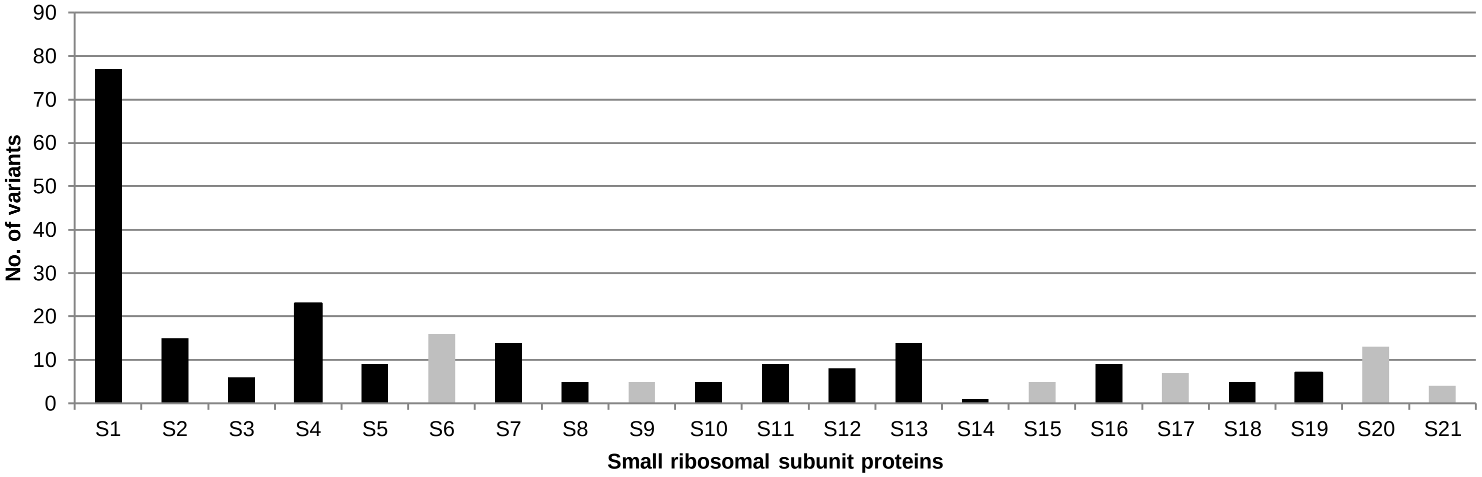

Figure S10. Number of large (A) and small (B) subunit r-protein variants among the *B. cereus* group. Black and gray colours represent, respectively, essential and non-essential r-proteins, as designated by Akanuma et al.(2012)[34]

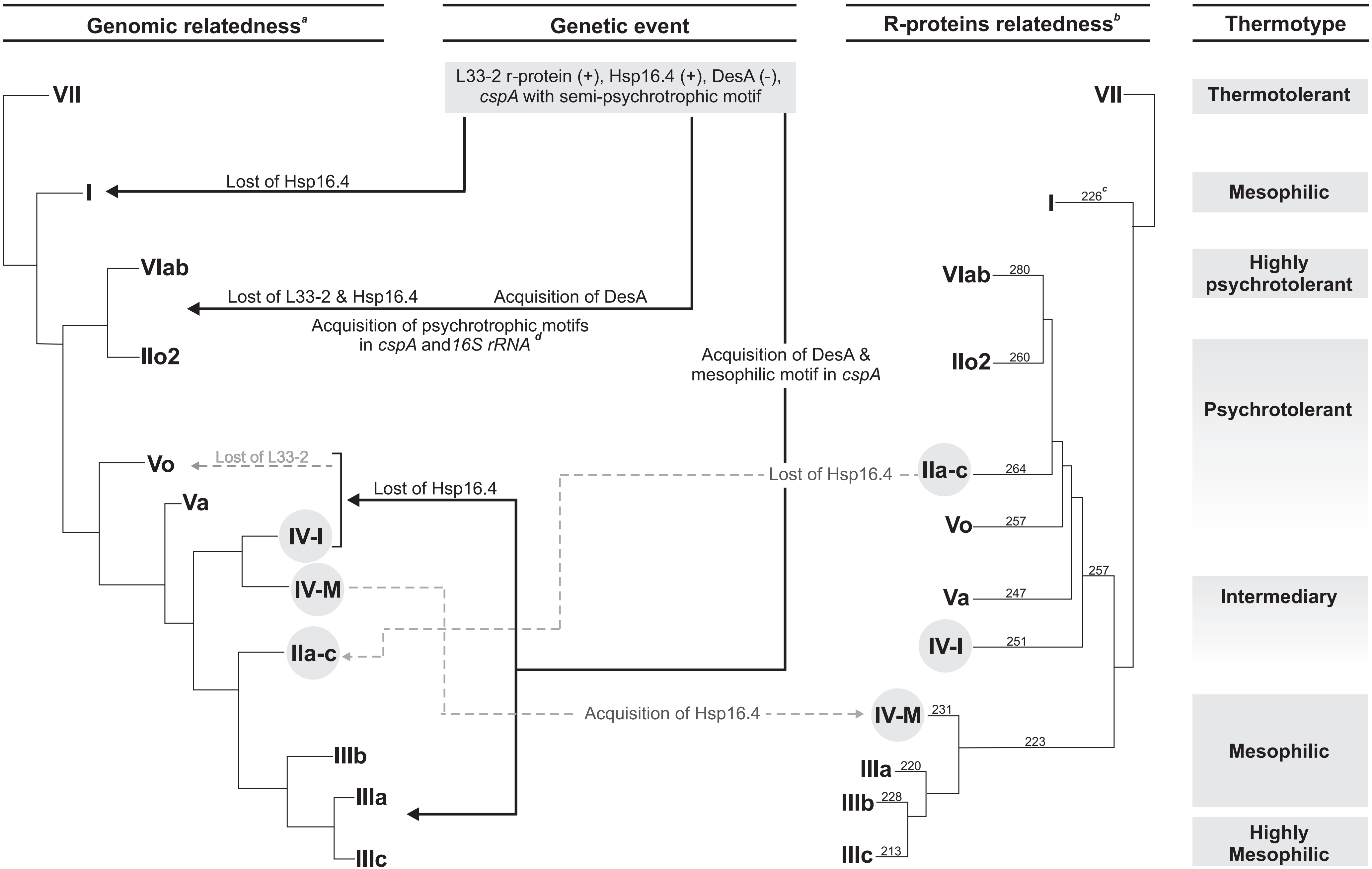

[a], genomic relatedness was adapted from the paper by Liu et al.[42]
[b], based on sequences identity of the 54 r-proteins (Fig. S2)
[c], the overall number of amino acids differences in the 54 r-proteins regarding the r-cluster VII as the reference
[d], the psychrotrophic motif in *16S rRNA* (one copy) was present only in two strains from the r-cluster IIo2

Figure S11. Comparison of genomic and r-proteins relatedness between the *B. cereus* group thermotypes and plausible genetic events in their evolution. The r-clusters with inconsistent genomic and r-proteins relatedness are highlighted in gray circles.

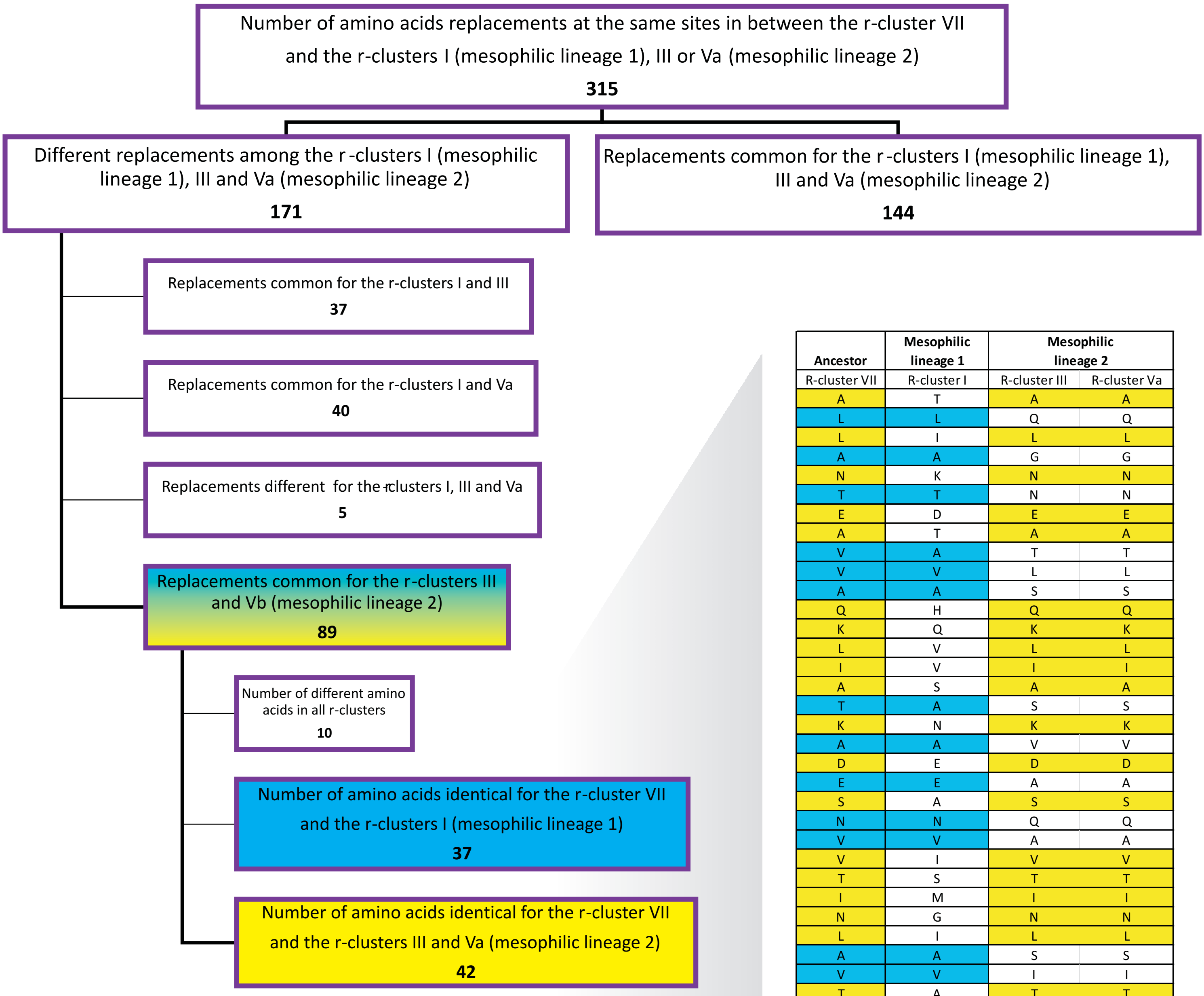

Ancestor	Mesophilic lineage 1	Mesophilic lineage 2	
R-cluster VII	R-cluster I	R-cluster III	R-cluster Va
A	T	A	A
L	L	Q	Q
L	I	L	L
A	A	G	G
N	K	N	N
T	T	N	N
E	D	E	E
A	T	A	A
V	A	T	T
V	V	L	L
A	A	S	S
Q	H	Q	Q
K	Q	K	K
L	V	L	L
I	V	I	I
A	S	A	A
T	A	S	S
K	N	K	K
A	A	V	V
D	E	D	D
E	E	A	A
S	A	S	S
N	N	Q	Q
V	V	A	A
V	I	V	V
T	S	T	T
I	M	I	I
N	G	N	N
L	I	L	L
A	A	S	S
V	V	I	I
T	A	T	T
A	S	A	A
L	L	M	M
M	I	M	M
L	V	N	N
L	L	I	I
E	E	A	A
V	A	V	V
V	I	V	V
K	T	K	K
V	V	L	L
E	N	K	K
E	D	E	E
N	N	S	S
P	V	I	I
A	T	A	A
L	L	I	I
V	V	M	M
V	A	E	E
E	K	E	E
T	N	T	T
E	D	E	E
E	E	H	H
V	M	V	V
Q	Q	L	L
D	E	D	D
G	D	G	G

Figure S12. Number of amino acids replacements in the 54 r-proteins of the *B. cereus* group between the r-cluster VII (denoted as ancestor) and the r-clusters I (denoted as mesophilic lineage 1) as well as the r-clusters III (representing mesophilic branch) and Va (representing intermediary mesophilic-psychrotolerant branch) and collectively denoted as mesophilic lineage 2. Replacements were identified based on an alignment of concatenated consensus sequences of 54 r-proteins (6808 amino acids) from the r-clusters.

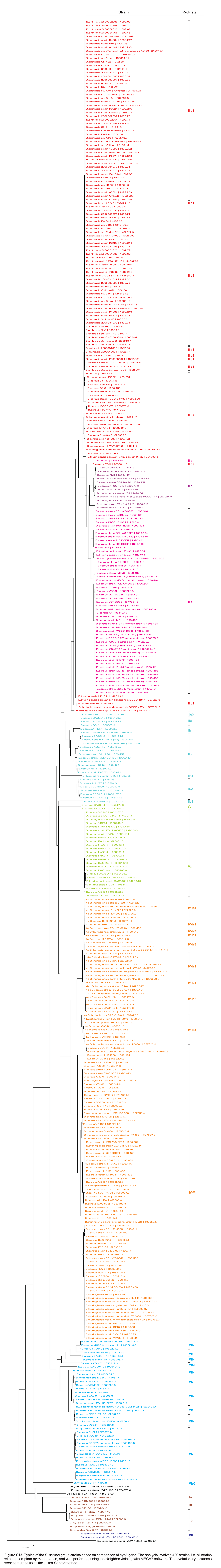

Figure S13. Typing of the *B. cereus* group strains based on comparison of *pycA* gene. The analysis involved 420 strains, i.e. all strains with the complete *pycA* sequence, and was performed using the Neighbor-Joining with MEGA7 software. The evolutionary distances were computed using the Jukes-Cantor method.
